# A comprehensive atlas of nuclear sequences of mitochondrial origin (NUMT) inserted into the pig genome

**DOI:** 10.1186/s12711-024-00930-6

**Published:** 2024-09-16

**Authors:** Matteo Bolner, Samuele Bovo, Mohamad Ballan, Giuseppina Schiavo, Valeria Taurisano, Anisa Ribani, Francesca Bertolini, Luca Fontanesi

**Affiliations:** Animal and Food Genomics Group, Division of Animal Sciences, Department of Agricultural and Food Sciences, University of Bologna, Viale Giuseppe Fanin 46, 40127 Bologna, Italy

## Abstract

**Background:**

The integration of nuclear mitochondrial DNA (mtDNA) into the mammalian genomes is an ongoing, yet rare evolutionary process that produces nuclear sequences of mitochondrial origin (NUMT). In this study, we identified and analysed NUMT inserted into the pig (*Sus scrofa*) genome and in the genomes of a few other Suinae species. First, we constructed a comparative distribution map of NUMT in the Sscrofa11.1 reference genome and in 22 other assembled *S. scrofa* genomes (from Asian and European pig breeds and populations), as well as the assembled genomes of the Visayan warty pig (*Sus cebifrons*) and warthog (*Phacochoerus africanus*). We then analysed a total of 485 whole genome sequencing datasets, from different breeds, populations, or *Sus* species, to discover polymorphic NUMT (inserted/deleted in the pig genome). The insertion age was inferred based on the presence or absence of orthologous NUMT in the genomes of different species, taking into account their evolutionary divergence. Additionally, the age of the NUMT was calculated based on sequence degradation compared to the authentic mtDNA sequence. We also validated a selected set of representative NUMT via PCR amplification.

**Results:**

We have constructed an atlas of 418 NUMT regions, 70 of which were not present in any assembled genomes. We identified ancient NUMT regions (older than 55 million years ago, Mya) and NUMT that appeared at different time points along the Suinae evolutionary lineage. We identified very recent polymorphic NUMT (private to *S. scrofa,* with < 1 Mya), and more ancient polymorphic NUMT (3.5–10 Mya) present in various *Sus* species. These latest polymorphic NUMT regions, which segregate in European and Asian pig breeds and populations, are likely the results of interspecies admixture within the *Sus* genus.

**Conclusions:**

This study provided a first comprehensive analysis of NUMT present in the *Sus scrofa* genome, comparing them to NUMT found in other species within the order Cetartiodactyla. The NUMT-based evolutionary window that we reconstructed from NUMT integration ages could be useful to better understand the micro-evolutionary events that shaped the modern pig genome and enriched the genetic diversity of this species.

**Supplementary Information:**

The online version contains supplementary material available at 10.1186/s12711-024-00930-6.

## Background

The complex evolutionary history of mammalian genomes has been punctuated by numerous events that determined the integration of new sequences into the nuclear chromosomes, which largely influenced their architecture, composition and function [1]. Among the sources of new nuclear sequences, the horizontal transfer of mitochondrial DNA (mtDNA) fragments into the nuclear chromosome DNA has produced, over the evolutionary ages, nuclear DNA sequences of mitochondrial origin, known as NUMT [2–4]. The accumulation of mtDNA derived fragments over time determined a total NUMT contribution on the whole mammalian nuclear genome sequence content that varies substaintially across species ranging from approximately 0.001 to 0.1% [2, 3]. The transfer of mtDNA sequences into the nuclear genome likely occurred through non-homologous end joining at double-stranded breaks in the nuclear genome. This process may have been facilitated by events such as transient breaches in the mitochondrial membrane, autophagy leading to the release of DNA in the cytoplasm, misuse of nucleic acid or protein import machinery, or fusion between the heterotypic membranes of the mitochondrion and nucleus [5–10].

Ancient NUMT integrations produced molecular fossil sequences that contain interesting evolutionary information [11–16]: (i) after their insertion into the nuclear genome, these sequences are no-longer under the evolutionary constrains of the mitochondrial genome and accumulate mutations at the rate of the nuclear genome, proportionally degrading the sequence in relation to the integration age; (ii) orthologous chromosome regions in phylogenetically related species, subspecies or lines may or may not contain the NUMT, depending on the timing of the taxonomic group radiation that occurred after or before the integration age of the mitochondrial derived DNA sequence.

As extensively demonstrated in humans, the integration of NUMT into the nuclear genome is still an ongoing, albeit rare, process, with significant implications. De novo NUMT integration in the human germ line has been estimated to occur once in every 10^4^ births, whereas it appears to be a much more frequent event in cancers (once every 10^3^ cancers [17]). Germ line integration can have harmful effects when it disrupts protein-coding genes [18–23]. Some NUMT can also function as regulatory elements or contribute to the creation of novel exons [24].

In many studies, NUMT have been discovered inadvertently during the analysis of the authentic mitochondrial genome. If not recognized, they can cause several problems and biases in the interpretation of results related to mtDNA pathogenic variants, mtDNA heteroplasmy and mtDNA phylogenetic reconstructions [25–30]. Some problems have also been reported in livestock species. For example, it is well known what happened in goat where NUMT were mistakenly included in the reference mtDNA sequence, leading to distorted phylogenetic and domestication pathways until the authentic mtDNA was reconstructed [31].

In general, what is at present known on the NUMT included in the genomes of livestock species is far less than what has been reported for the human genome. The available reference nuclear genomes of several livestock species have been mined to gain initial insights into the presence and distribution of NUMT. For instance, Hazkani-Covo et al. [3], using BLASTN, searched all reference genomes available in 2010, including the first versions of cattle, chicken, rabbit, and horse genomes. Pereira and Baker [32] mined the reference chicken draft genome, while Schiavo et al. [33] analysed the reference turkey genome. All studies on avian genomes consistently reported a relatively lower number of NUMT in birds compared to mammals. Some researchers have mined multiple versions of the cattle reference genome and compared the distribution of NUMT across cattle genome assemblies [34–36]. Other studies have recently mined the available reference genomes for sheep, goat, alpaca and rabbit using similar descriptive approaches [37–40]. The horse genome was investigated by Nergadze et al. [41] who identified polymorphic NUMT (in terms of presence or absence of the insertion) segregating within the species. Polymorphic NUMT have been also identified in the pig genome by Schiavo et al. [14]. Surprisingly, the estimated age of some of these porcine polymorphic NUMT predated the speciation time of the *Sus scrofa* species, supporting the hypothesis that these polymorphisms might have been originated from interspecies admixture [14]. At the same time, the study provided an initial overview of NUMT in pig by mining the Sscrofa10.2 genome version [14].

Another reference genome version is now available for the pig (Sscrofa11.1; [42]) in addition to several other breed-derived whole genome assemblies [2, 42, 43]. Many other projects have resequenced the genome of tens or hundreds of pigs from different breeds and the derived genomic information has been deposited in public repositories (e.g. [44–46]).

In this study, we mined all recently available pig assembled genome versions, including the assembled genomes of *Sus cebifrons* and *Phacochoerus africanus*, along with hundreds of publicly available and newly produced whole genome sequencing (WGS) datasets generated from many different pig breeds and populations. Our goal was to identify NUMT inserted into the pig genome. Through this analysis, we created a comprehensive catalog of porcine NUMT, annotated with various phylogenetic features. This catalog can be valuable in understanding the evolutionary paths of the modern *Sus scrofa* genome, compared to other species within the Suinae subfamily.

## Methods

### Mined assembled genomes and whole genome sequencing datasets

The following assembled genomes were downloaded from the National Center for Biotechnology Information (NCBI) Assembly Database ([47], accessed on the 1st of July 2023): the latest *Sus scrofa* reference genome versions Sscrofa11.1 and Sscrofa10.2 (both constructed based on a Duroc pig: TJ Tabasco, Duroc 2–14); the drafted genome assemblies for 20 domestic pig breeds and populations [ten European-derived breeds/lines or populations: Berkshire, Duroc, Hampshire, Landrace, Large White, Nero Siciliano, Pietrain, composite breed (Duroc/Landrace/Yorkshire crossbred barrow), Kenyan pig, Ossabaw Island hog; nine Asian (Chinese) breeds or populations: Bama miniature pig, Bamei, Jinhua, Meishan, Ningxiang, Ronchang, Tibetan and Wuzhishan; one Asian/European composite breed: Göttingen Minipig] and for the European wild boar; one drafted genome assembly for the Visayan warty pig (*Sus cebifrons*) and two drafted genome assemblies for the common warthog (*Phacochoerus africanus*), which are the only other species of the family Suidae, subfamily Suinae, in addition to *Sus scrofa*, for which genome assemblies were available; the latest *Bos taurus* reference genome (ARS-UCD1.3) and the latest *Capra hircus* reference genome (ARS1.2), used for comparative and evolutionary analyses. Additional file 1: Table S1 lists detailed information on these assembled genome versions.

A total of 485 short read whole genome sequencing (WGS) datasets were investigated: 302 WGS datasets from individual pigs, with a sequencing depth of at least 10 × , belonging to 35 pig breeds/populations (18 European and 13 Asian breeds or derived breeds for a total of 177 and 68 datasets, respectively, and other 13 datasets from four lines/crossbreds of mixed Asian and European origin), European, Near East and Asian wild boars (for a total of 28 WGS datasets) and four other *Sus* species (*Sus barbatus*, *S. cebifrons, S. celebensis* and *S. verrucosus*, for a total of 16 WGS datasets), were retrieved from the European Nucleotide Archive (ENA [48]); 160 WGS datasets, with a sequencing depth of about 20 × , were from other de novo sequenced Italian pig breeds (35 Italian Landrace pigs, 90 Italian Large White pigs and 35 Italian Duroc pigs); 22 WGS datasets from pooled DNA samples of 21 European pig breeds (average sequencing depth of about 40 ×) and one from European wild boars (at about 10 ×), each derived from 30 to 35 animals [45]. Detailed information on all these WGS datasets is reported in Additional file 1: Table S2.

### Detection of NUMT in the reference and drafted genomes

The pipeline used to identify NUMT in the assembled genomes followed the method described by Schiavo et al. [14]. Initially, a total of 11 mtDNA genomes from Asian pig breeds (Jeuma, KP223728.1; Longlin, KM433673.1; Rongchang, KM044239.1; Sandu-black, KM094194.1; Wuji-Black, KM259826.1), European pig breeds (Reference mtDNA sequence, NC_000845.1; Duroc, AY337045.1; Hampshire, AY574046.1; Large White, KC250275.1; Mangalitsa, KJ746666.1) and Asian wild boar (FJ236998.1) were retrieved from NCBI and aligned using MAFFT v7.505 [49]. Subsequently, a consensus *Sus scrofa* mtDNA sequence was generated from this alignment using EMBOSS CONS [50]. This consensus sequence was then used to search for NUMT in both the *Sus scrofa* reference and assembled nuclear genomes. Additionally, the reference mitochondrial genomes of *S. cebifrons* (NC_023541.1), *P. africanus*, (NC_008830.1), *B. taurus* (NC_006853.1) and *C. hircus* (NC_005044.2) were downloaded from NCBI and used to search for NUMT in their respective nuclear genome assemblies. Detailed information regarding these mtDNA sequences can be found in Additional file 1: Table S3.

The *Sus scrofa* consensus mtDNA sequence and the mtDNA sequences of the other species were then circularized by concatenating two copies of the complete mtDNA, in order to capture matches extending over the boundaries of the linear sequence [14]. LAST software version 1256 [51] was used to query the listed assembled genomes by aligning the corresponding circularized mitochondrial genome. LAST software parameters can be adjusted to capture low identity that might be expected when aligning old NUMT to modern mtDNA sequences making it more appropriate than BLASTN in NUMT detection studies [14, 34, 36, 51, 52]. Following Tsuji et al. [52], LAST parameters used for the alignments were those considered suitable for detecting distant homology: + 1 for matches, − 1 for mismatches, 7 for gap-open penalty and 1 for gap-extension penalty. LAST software computes a score based on the expected number of alignments of a random sequence with the same length as the query and a random sequence with the same length as the database. A method to identify a score threshold that minimizes the identification of false positive matches, described by Tsuji et al. [52], uses the reversed mtDNA genome, obtained by reversal without complementation, which is then aligned to the reference nuclear genome. Matches obtained from this alignment can be considered spurious, as DNA sequences do not evolve through simple reversal. After applying this method we obtained alignments with scores between 32 and 53. A score of 32 corresponds to 0.016 expected alignments with greater or equal score between a random sequence with the same length as the query sequence, and random sequences with the same length as the database. As our aim was also to capture ancient integrated NUMT over the evolutionary lineage of the order Cetartiodactyla that then diverged into the families Bovidae and Suidae, to reduce the probability of filtering out very old NUMT useful for this aim, we therefore applied a score threshold of 30, which would correspond to about 0.02 expected alignments. LAST has a multiplicity parameter that aims to prevent repetitive matches between query and subject sequence; however, since in our case the mitochondrial query sequence is expected to have multiple matches with the nuclear sequence due to the multiple NUMT insertion events that occurred during genome evolution, we set the multiplicity parameter to 150,000, in order to capture all possible matches; we then filtered the results using last-postmask, to remove alignments originating from simple sequence or low complexity regions. Putative NUMT identified within 1000 bp of chromosome or contig extremities were excluded from the dataset due to uncertainty about the assembly quality of the region, and the inability to validate their orthology in other assembled genomes.

All LAST detected NUMT, including overlapping sequences and consecutive matches on the same chromosome regions (i.e. NUMT that were < 20 kb apart), were visually inspected using dot plots designed with the Seaborn Python library [53], including the mtDNA consensus sequence on the y axis and the nuclear genome sequence of the x axis.

Consecutive NUMT were considered part of the same NUMT region derived from a single putative insertional event that, after subsequent mutations (including insertions/deletions) in the nuclear genome, was rearranged and divided into its current structure with multiple NUMT matches (defining more NUMT sequences determining the same NUMT region). Additional events (i.e., duplications, rearrangements, or non-unique insertions) were identified when more complex patterns in consecutive nuclear sequences were observed when compared to the mtDNA sequence and were considered complex events.

### Detection of NUMT in short read whole genome sequencing datasets

Sequencing reads from the WGS datasets were aligned to the Sscrofa11.1 reference genome of *Sus scrofa* using the BWA alignment tool [54]. NUMT not included in the reference genome were identified following an adapted procedure based on Ju et al. [55] and Wei et al. [56]. First, breakpoints supported by at least 3 soft-clipped reads were identified in the aligned sequencing reads. All soft-clipped reads overlapping with each breakpoint were extracted and aligned with the reference mtDNA sequence of *S scrofa.* Alignments were filtered by keeping only those with sequence identity higher than 75% and an E-value lower than 0.0001. The filtered sequence coordinates were then concatenated to obtain the putative NUMT sequence for each breakpoint. Next, the breakpoint coordinates were overlapped with the coordinates of the NUMT regions detected with LAST and for which a position in the reference genome could be defined. This helped to identify which putative NUMT were novel, i.e., not present in the assembled genomes, or already present in the assembled genomes.

Putative novel NUMT were additionally filtered as follows. First, to filter out copy number variations and complex genomic regions, we discarded breakpoints where the average sequencing depth around the breakpoint was lower than six or higher than MD = d + 4 $\surd$ d [57], with *d* representing the average read depth of the sample. Breakpoints within 100 bp of each other across different samples were then grouped together and considered part of the same NUMT insertion. We then defined three other filters for each putative NUMT:

(i) R1, defined as the number of mapped reads supporting the breakpoints that were soft clipped for at least seven nucleotides and aligned with the mitochondrial genome, divided by the total number of mapped reads soft clipped for at least seven nucleotides (including soft-clipped portions not aligning to mtDNA). Putative NUMT were discarded if all breakpoints had R1 < 0.75 across all samples.

(ii) R2, defined as the number of reads soft-clipped at the breakpoint position divided by the total number of reads overlapping with the breakpoint. R2 was used to define the expected genotype of the NUMT in the analysed WGS dataset. NUMT were classified as homozygous for the non-reference NUMT insertion if R2 was above 0.80, and heterozygous for values between 0.30 and 0.80. Values below 0.30 indicated homozygosity for the reference allele, indicating the absence of NUMT insertions. Putative NUMT with breakpoints having R2 < 0.30 across all samples were therefore excluded. This filter was not applied to putative NUMT obtained from pooled samples, as they contained DNA from 30 to 35 different animals, each with potentially different genotypes.

(iii) Putative NUMT insertions with a sequence length shorter than 30 bp were discarded, as 30 bp is the smallest length of a NUMT insertion identified in the assembled reference genome using LAST.

Putative NUMT passing all filtering criteria and found in at least two WGS datasets (to avoid false positive identifications of germline inserted NUMT) were then considered as novel NUMT (i.e. NUMT not included in the Sscrofa11.1 pig reference genome and/or other assembled genomes, but present in other pig genomes derived from the WGS datasets). NUMT sequences belonging to the same NUMT regions were defined as described above.

The frequency of the NUMT carriers was calculated based on the WGS datasets that either had or did not have the considered NUMT, providing information on within species polymorphic NUMT (i.e., presence/absence of the insertion), both for novel NUMT and NUMT already present in the reference genomes.

We then assembled the sequence of the NUMT selected for in vitro validation, following the method of Dayama et al. [58]: (i) soft-clipped and discordant read pairs were extracted from each NUMT across all samples in which the NUMT was found; (ii) CAP3 software ([59], version date 02/10/15) was used for the assembly of the reads into a contig; and (iii) the resulting contigs were annotated by aligning them with the *Sus scrofa* reference mitochondrial genome sequence.

Preliminary information on the porcine NUMT regions detected by mining assembled pig genomes and WGS datasets has been previously reported by us in proceedings of a conference [60].

### Detection of orthologous NUMT across pig breeds and across species

To confirm the presence of orthologous NUMT across the assembled pig genomes, the whole genome sequence of each assembly was aligned with the reference genome Sscrofa11.1 and vice versa using LAST [61], following the guidelines for closely related genomes provided by the authors [62]. Initially, a LAST database was created for each assembly using the NEAR seeding scheme, which is effective for identifying similarities with many gaps, such as polymorphic NUMT insertions. After having determined the optimal parameters for each alignment with the *last-train* program, the alignment was performed with an EG2 threshold of 0.05, selecting only significant alignments that would be expected to occur by chance at a rate ≤ 0.05 times per pair of random sequences of length 1 billion each. Subsequently, *last-split* was used to establish one-to-one alignments between the two genomes to exclude suboptimal secondary alignments from the resulting dataset. For all candidate alignments of query sequences, *last-split* cuts a unique best alignment for each part of each query. In this way, each part of the two genomes aligns to at most one part of the other genome. Alignments with a *mismap* probability > 10^–5^ were discarded. Lastly, *last-postmask* was used to discard alignments caused by simple sequence or low complexity regions.

After aligning the two genomes, the coordinates for each NUMT region were extracted from the alignment file; if the aligned portion corresponding to the NUMT region overlapped with a NUMT region in the other genome, the two regions were considered orthologous. If the NUMT region aligned fully and without gaps to a sequence which was not identified as NUMT in the other genome, we considered the other sequence as a NUMT and added its information in the dataset. This approach helped correct false negative results that can occur in across-assembled genome alignments using alignment tools. If the NUMT region corresponded to gaps only, it was deemed absent in the corresponding assembled genome and could be potentially considered polymorphic within the *Sus scrofa* species, with the insertion present or absence in some of the assembled genomes. Confirmation of polymorphic NUMT insertion within a species was then obtained using WGS datasets (see above).

This approach was also applied to identify orthologous NUMT across Suinae species (*P. africanus* and *S. cebifrons* vs. *S. scrofa*), considering the possibility of identifying sequence matches across these closely related species. Alignments corresponding to a gapped sequence on the NUMT coordinates were annotated as recent NUMT, indicating their insertion occurred after the divergence between the species.

An alternative approach was used to detect orthologous NUMT across more distantly related species (i.e. *B. taurus* and *C. hircus vs. S. scrofa*). First, the set of orthologous genes and syntenic chromosome regions between species was obtained from Ensembl Compara [43]. This information was used to identify whether NUMT inserted in the two genomes were located in syntenic regions, by checking the annotated five upstream closest genes and five downstream closest genes in both pairwise compared species. To avoid annotation biases across genomes, stringent criteria were used to declare orthologous NUMT: (i) at least 50% of the genes resulted syntenic in the two species; and (ii) there was a complete or partial (more than 40%) sequence alignment overlap on the same original mtDNA genome region of the two respective NUMT sequences or of the two respective NUMT regions. Two genomes of distantly related species to *S. scrofa* (*B. taurus* and *C. hircus*) were chosen in order to avoid biases preventing the identification of orthologous NUMT in *S. scrofa* potentially due to: (i) loss of the orthologous NUMT in one or the other genome over evolutionary time; (ii) mis-assembly of the NUMT region in one or the other genome. The cattle and goat genome versions used for comparison were those with the highest N50 among the assembled genomes of the species of the order Cetartiodactyla.

### Phylogenetic analyses

NUMT sequences larger than 150 bp were included in the phylogenetic trees obtained using Bayesian analysis with Markov Chain Monte Carlo (MCMC) from the BEAST program [63]. The following mitochondrial genome sequences were used to build the trees and for all other phylogenetic analyses:

(i) Species of the family Suidae: *Pecari tajacu* (NC_012103.1), *Babyrousa babyrussa* (OK539643.1)*, Phacochoerus africanus* (NC_008830.1), *Porcula salvania* (NC_043879.1), *Potamochoerus porcus* (NC_020737.1), *Sus barbatus* (NC_026992.1), *Sus cebifrons* (NC_023541.1), *Sus celebensis* (NC_024860.1), *Sus scrofa* (NC_000845.1) and *Sus verrucosus* (NC_023536.1).

(ii) Outgroup species: *Bos taurus* (NC_006853.1).

All of these sequences were aligned with each NUMT sequence using LAST to extract the portion of mitochondrial sequences of interest, which were then aligned using MAFFT v7.505 [49]. The resulting alignment was used as input for BEAST [63] divided into two groups: one group containing only the NUMT sequence, and the other containing all other sequences. A local fixed molecular clock was selected to allow different mutation rates between the two groups that could account for the different mutation rate between nuclear and mitochondrial genomes. Default settings were used including the Hasegawa-Kishino-Yano (HKY) substitution model with equal base frequencies [64], and the Yule process model for speciation as tree prior model [65]. A default MCMC chain length of 10^6^ was selected, followed by a 10^5^ burn-in (10% of the chain length) to obtain the maximum clade credibility (MCC) tree.

Based on the assumption that the presence of the same NUMT region in two genomes indicates that the insertion event took place in a common ancestor, each NUMT region was analysed and listed in one of the following categories based on the divergence times between species:

- Very recent insertion events, if the same NUMT regions were found only in the *S. scrofa* genomes and not in *S. cebifrons* and *P. africanus*, or in the *S. cebifrons* genome only, indicating an insertion time point more recent than the divergence between the two *Sus* species, ~ 3.5 millions of years ago (Mya), mid Pliocene, based on a classical phylogenetic interpretation that does not consider reticulate evolutionary pathways [66] (defining the age class of 0 < Mya ≤ 3.5; indicated as < 3.5);
- Recent insertion events, if the same NUMT regions were found in both the *S. scrofa* and *S. cebifrons* genomes, but not in the *P. africanus* genome, indicating that the insertions occurred after the divergence from *P. africanus* (estimated to have happened about 10 Mya, late Miocene) and before the divergence from *S. cebifrons* (~ 3.5 Mya) [66]; alternatively, if the NUMT region was found only in *P. africanus* (and not in *Sus* species) it was considered an insertion event more recent than 10 Mya, occurring in the *Phacochoerous* evolutionary lineage after the divergence with the *Sus* lineage (defining the age class of 3.5 < Mya ≤ 10; indicated as 10–3.5);
- Intermediate insertion events (which might have occurred between ~ 55 to 10 Mya), if the same NUMT regions (orthologous regions) were found in both *S. scrofa* and *P. africanus* but not in *B. taurus* and *C. hircus* (defining the age class of 10 < Mya ≤ 55; indicated as 55–10);
- Ancient insertion events, if the same NUMT regions (orthologous regions) were found in either *B. taurus* or *C. hircus* and in the *S. scrofa* genome, indicating that the insertions occurred before the divergence of Suidae and Ruminantia, that is estimated to have occurred ~ 55 Mya, during the early Eocene [67, 68] (defining the age class of Mya > 55; indicated as > 55);

Based on this classification, two methods were applied to obtain a more precise estimation of the evolutionary insertion time within the identified time ranges (NUMT derived from the mtDNA D-loop regions were not used for age estimation due to the uncertainty in predicting ancestral D-loop sequences).

The first method, described by Dayama et al. [12], was applied to both recently inserted NUMT regions (≤ 10 Mya) and very recently inserted NUMT regions (≤ 3.5 Mya), identified as defined above, where the summed size of their related NUMT sequences was > 150 bp and the average sequence identity was over 80%. This identity threshold was chosen to avoid classifying as recent those NUMT regions that were not found in *P. africanus* but may have been inserted before the two species diverged, as the low sequence identity suggests. These NUMT may have not been identified in the *P. africanus* genome version due to excessive sequence degradation or issues with the assembly of this genome.

For recent NUMT, an ancestral mtDNA sequence for the Suinae lineage was inferred using the following mitochondrial genomes: *Phacochoerus africanus*, *Porcula salvania*, *Potamochoerus porcus*, *S. barbatus*, *S. cebifrons, S. celebensis*, *S. scrofa* and *S. verrucosus*. The same was done for very recent NUMTs, by reconstructing the ancestral mtDNA sequence of the *Sus* lineage, using only the mitochondrial genomes of *Sus* species from the dataset described above. These sequences were rotated to synchronize their starting points and aligned using MAFFT software version 7.505 [49]. The ancestral mtDNA sequence was then reconstructed using IQTREE 2.0.3 [69]. For the modern sequence, the consensus *S. scrofa* mtDNA sequence reported above was used for *S. scrofa* NUMT, the *S. cebifrons* reference sequence was used for *S. cebifrons* NUMT and the *P. africanus* reference sequence was used for *P. africanus* NUMT. All these mtDNA sequences were circularized to allow full alignments of the NUMT sequences. Each NUMT sequence was then aligned with both ancestral and modern sequences using LAST [51]. A multiple sequence alignment was then obtained with MAFFT [49] including the NUMT sequence, the ancestral mtDNA and the modern mtDNA. The alignment was inspected to identify positions where modern and ancestral sequences differed. Of these positions ($ancestral\_vs\_modern$), those where the NUMT sequence matches the modern sequence were counted ($numt\_vs\_modern$). From these values, the allele matching ratio ($amr$) was calculated:

1 $$\frac{numt\_vs\_modern}{ancestral\_vs\_modern},$$

Considering the divergence time ($T$) between Suinae species of 10 Mya and between *Sus* species of 3.5 Mya [65], the insertion time along the lineage was computed as following:

2 $$age=\left(1-amr\right)\times T,$$

where age is expressed in Mya. For NUMT regions containing multiple fragments the age was computed by summing the total number of sites of all corresponding NUMT sequences to obtain the allele matching ratio ($amr$), as described by Dayama et al. [12].

The second method used to estimate the insertion age was applied to NUMTs inserted > 10 Mya (i.e., intermediate and ancient NUMT, as defined above), and to regions with an average sequence identity lower than 80%. The applied method was based on Fukuda et al. [70] who used the Kimura distance [71] between sequences and their mutation rate.

This method was applied only on NUMT regions containing sequences longer than 150 bp and not overlapping with the D-loop region of the mitochondrial genome; in regions containing multiple sequences, the region age was computed on the longest NUMT sequence. For this analysis, each NUMT sequence was individually aligned using LAST with all the mitochondrial genomes of Suinae plus *B. taurus;* since these NUMT insertions are estimated to have occurred before the divergence between Suinae and other mammalian groups, the inclusion of mitochondrial genomes belonging only to Suinae could provide a biased estimation; therefore, the mitochondrial genomes of *Pecari tajacu* (family Tayassuidae) and *B. taurus* (suborder Ruminantia, family Bovidae) were also included. A multiple sequence alignment was then performed between all mitochondrial sequences obtained from the alignments and the NUMT sequence using MAFFT [49] with 1000 cycles of iterative refinement. A distance matrix was then computed using the Kimura distance (K80) with EMBOSS *distmat* [50]. The smallest distance between NUMT and mtDNA sequences was considered the approximate point of insertion along the lineage. The average distance between the NUMT and all species with a lower distance than the insertion point identified above was computed ($K$) and used to calculate the NUMT insertion age ($t$) with the following formula:

3 $$t=\frac{K}{{V}_{mt}+{V}_{numt}},$$

where ${V}_{mt}$ is the mitochondrial mutation rate and ${V}_{numt}$ the evolutionary rate of the NUMT sequence. For *V*_*numt*_, we tested three mutations rates: (i) the average mammalian nuclear mutation rate (2.2E–9 mutations per site/year), defined by Kumar and Subramanian [72]; (ii) the mean rate of 2.48E–9 mutations per site per year phylogenetically estimated across wild ruminants [73] adopted to analyse warthog evolution by Garcia-Erill et al. [74]; (iii) the mutation rate of 1.2E–9 per site per year estimated in domestic pigs by Zhang et al. [75] using pedigree information. When a calculated age was needed for subsequent analyses, we used the mutation rate defined by Kumar and Subramanian [72] for the two age classes of insertion age estimated to occur from 10 to 55 Mya (intermediate insertion age) and > 55 Mya (ancient insertion age). The other two mutation rates were used for comparative evaluations of estimated insertion age.

Since the mitochondrial mutation rate is known to vary significantly between regions of the mitochondrial genome [76], a specific ${V}_{mt}$ was computed for every NUMT mitochondrial sequence, using the *average* of the divergence time between species ($T$) and the average of the evolutionary distances ($K$) obtained from the distance matrix, using the following formula:

4 $${V}_{mt}=avg\left(\frac{K}{2}T\right).$$

The divergence times used for each species pair were obtained from Frantz et al. [66].

Information derived from the NUMT spread throughout the Suinae nuclear genomes and retrieved from WGS datasets (as mentioned earlier) was used to cluster different pig breeds together with *S. barbatus*, *S. cebifrons*, *S. celebensis*, and *S. verrucosus* and verify the already known phylogenetic relationships between the considered taxa [66]. Only pig breeds with at least 10 WGS datasets were included in this analysis. Cluster analysis was conducted using the Seaborn Python library [53], using the UPGMA algorithm (unweighted pair group method with arithmetic mean) and the Euclidean distance metric.

### Annotation of NUMT

The upstream and downstream genomic sequences flanking the detected NUMT regions were analysed to identify: (i) repetitive elements within 1 kb from the inserted NUMT regions using NCBI RefSeq RepeatMasker data; (ii) GC content within 1 kb from the inserted NUMT regions; (iii) the position of the insertion relative to gene features (coding exons, introns, 5' and 3' untranslated regions or intergenic regions; and (iv) the closest gene from the annotated features of the NCBI RefSeq Sscrofa11.1 genome version.

### In vitro validation of NUMT

Ten primer pairs were designed to confirm the presence of seven NUMTs found in the *S. scrofa* assembled genomes and three novel NUMT found in the WGS datasets only. These NUMTs were chosen to represent a subset of different phylogenetic categories dating back less than 10 Mya. All detailed information on these NUMT, including PCR primers and PCR conditions is reported in Additional file 2: Table S4. DNA from pigs of three cosmopolitan breeds (30 Italian Large White, 30 Italian Landrace, 30 Italian Duroc), 48 pigs from six Italian autochthonous pig breeds (8 Apulo Calabrese, 8 Casertana, 8 Cinta Senese, 8 Mora Romagnola and 8 Sarda) and 8 Italian wild boars was used to confirm the presence/absence of the inserted NUMT. Amplified fragments were separated by electrophoresis in 2.5% agarose gels and visualized with GelRed Nucleic Acid Gel Stain (Biotium Inc., Hayward, CA, USA). Amplicons obtained from all primer pairs for at least six different animals were subjected to for Sanger sequencing.

## Results

### Detection of NUMT in the assembled genomes: general statistics

**Fig. 1:**
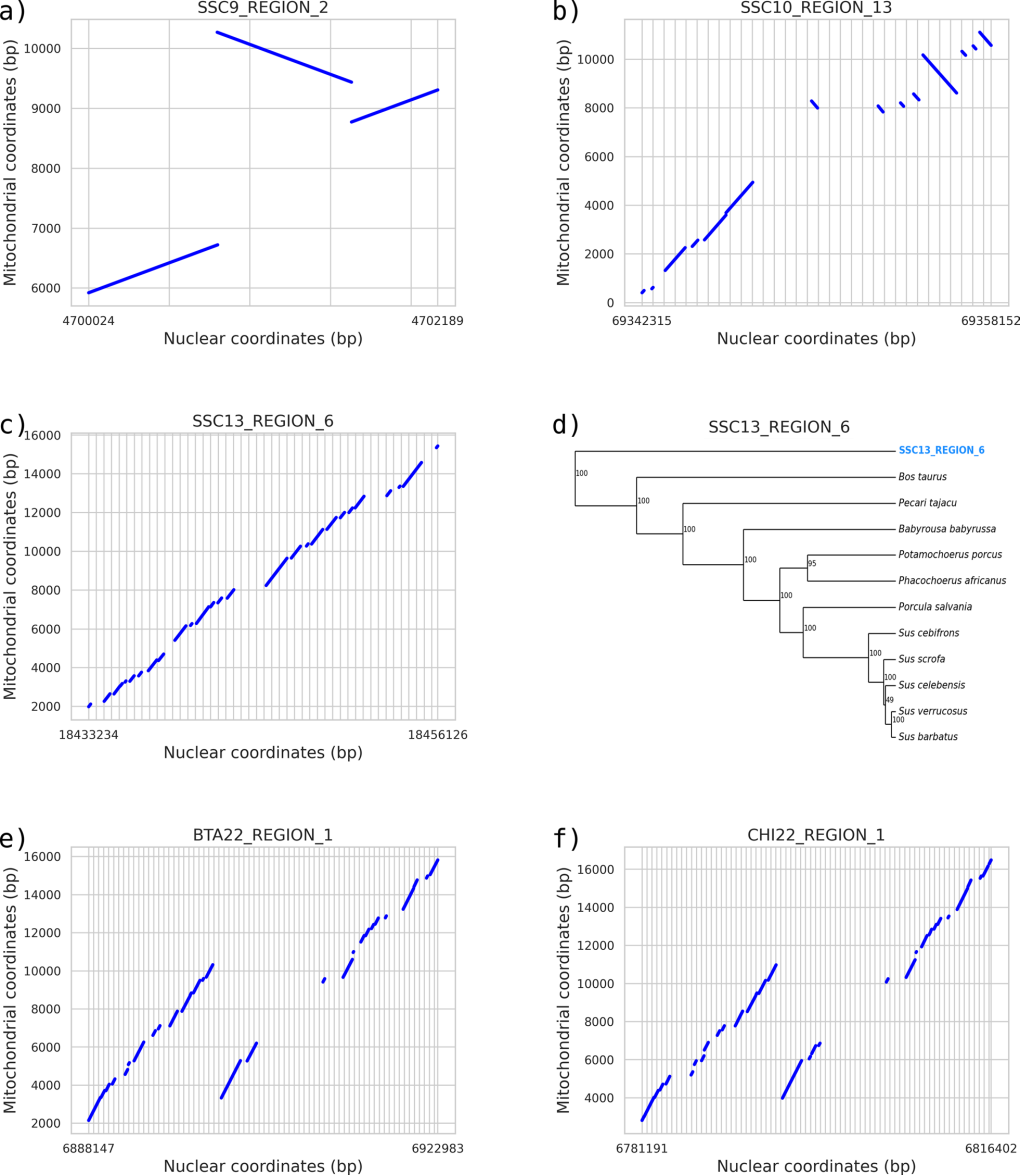
Examples of NUMT regions identified in the reference pig genome, including orthologous information for one of them. The lines in each subplot represent NUMT sequence fragments, with their coordinates in the nuclear genome on the X axis (nucleotide positions), and their coordinates on the mitochondrial genome on the Y axis (nucleotide positions). These NUMT were selected because they represent different examples of NUMT regions. **a** SSC9_REGION_2 is constituted by an inverted NUMT sequence in the middle part of the NUMT region. **b** SSC10_REGION_13 shows a highly fragmented NUMT region which underwent an inversion in the middle of the original integrated sequence. **c** SSC13_REGION_6 shows a NUMT region covering nearly the entire mitochondrial genome. **d** A phylogenetic tree for NUMT SSC13_REGION_6. In this tree, the posterior probability computed by BEAST is shown alongside each node, the NUMT nuclear sequence is colored in blue and the other mitochondrial sequences are colored in black. **e** An orthologous region for this NUMT was identified in the cattle genome (*B. taurus* chromosome 22). **f** An orthologous region for this NUMT was also identified in the goat genomes (*C. hircus* chromosome 22)

By mining the 23 *S. scrofa* assembled genomes (including the Sscrofa11.1 and Sscrofa10.2 genome versions), we identified between 310 and 340 NUMT regions, comprising a total of 573 to 917 NUMT sequences (Table 1). NUMT regions were defined as consecutive and fragmented NUMT sequences, putatively assumed to have originated from a single insertional event that subsequently underwent fragmentation due to various mutational events over time. Figure 1 shows some of the NUMT regions identified in the Sscrofa11.1 reference genome (including a NUMT region that covered nearly the entire mitochondrial genome). Among the detected NUMT regions, 13 were classified as complex regions, due to the presence of sequence duplications and/or inversions and fragmented mtDNA insertions. Detailed information on the NUMT found in the assembled genomes can be found in Additional file 2: Table S6.

**Table 1:** Number of NUMT sequences and regions identified in the *Sus scrofa* and other Suinae assembled genomes

Genome^a^	Breed/population or species^b^	Total number of NUMT sequences	Number of NUMT regions with multiple fragments	Number of singleton NUMT regions	Total number of NUMT regions
Sscrofa11.1 (REF11)	Duroc*	903	91	247	338
Sscrofa10.2 (REF10)	Duroc*	679	77	252	329
Berkshire_pig_v1 (BE)	Berkshire*	684	73	241	314
Ninghe_Sus_1(DU)	Duroc*	830	85	244	329
Hampshire_pig_v1 (HS)	Hampshire*	709	75	246	321
CAU-K (KE)	Kenyan domestic pig*	802	83	251	334
Landrace_pig_v1 (LA)	Landrace*	715	76	245	321
Large_White_v1 (LW)	Large White*	716	77	243	320
NSME_pig_1.2 (NS)	Nero Siciliano*	781	82	242	324
Pietrain_pig_v1 (PT)	Pietrain*	741	76	246	322
USMARCv1.0 (UM)	USMARC Crossbreed*	917	99	239	338
ASM2165605v1 (WB)	Wild Boar*	741	76	249	325
ASM2471841v1 (OB)	Ossabaw Island hog*	573	66	245	311
SscrofaMinipig (MP)	Ellegaard Gottingen Minipig**	699	76	243	319
Bamei_pig_v1 (BA)	Bamei***	864	84	246	330
ASM764409v1 (BM)	Bama Miniature Pig***	743	80	248	328
Jinhua_pig_v1 (JI)	Jinhua***	701	77	243	320
Meishan_pig_v1 (ME_1)	Meishan***	795	81	242	323
ASM1795798v1 (ME_2)	Meishan***	844	88	240	328
ASM2056790v1 (NX)	Ningxiang***	697	75	247	322
Rongchang_pig_v1 (RC)	Rongchang***	611	73	237	310
Tibetan_Pig_v2 (TI)	Tibetan***	702	78	252	330
minipig_v1.0 (WU)	Wuzhisan***	832	92	248	340
Sus_cebifrons.v1 (SC)	Visayan warty pig (*S. cebifrons*)	819	83	239	322
ROS_Pafr_v1 (PA1)	Common warthog (*P. africanus*)	886	81	237	318
CAU-W (PA2)	Common warthog (*P. africanus*)	722	75	236	311

^a^ Name of the genome assembly and its abbreviation (within the bracket). More information of these assembled genomes is reported in Additional file 1: Table S1

^b^ Breed/population or species of the animal from which the DNA was used for the genome assembly (all genomes except Sus_cebifrons.v1, ROS_Pafr_v1 and CAU-W are from *Sus scrofa*: “*” indicates European-derived breeds/lines or populations; “**” indicates the Asian/European composite breed; “***” indicates the Chinese breeds or populations)

**Fig. 2:**
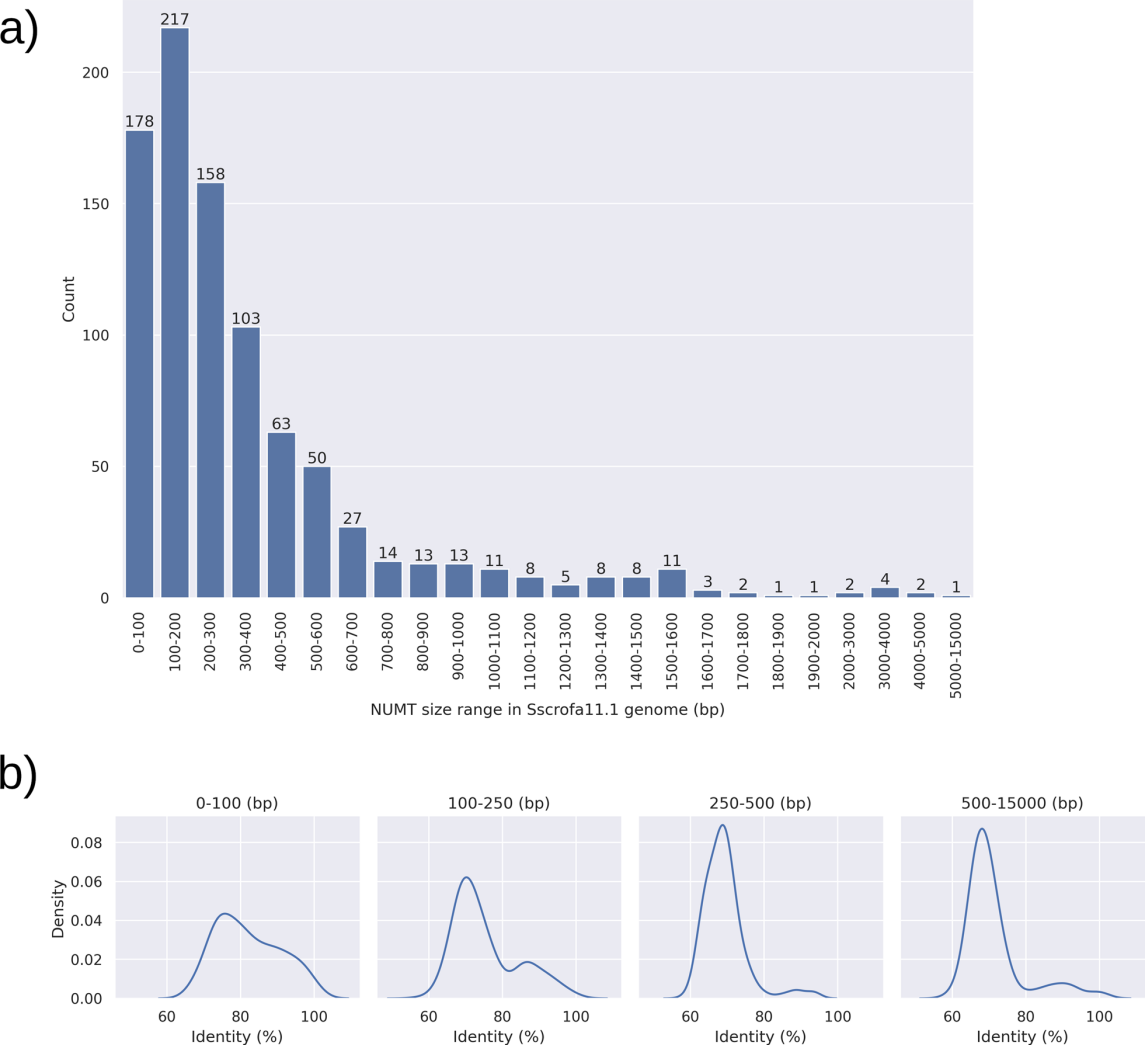
Distribution of NUMT identified in Sscrofa11.1 by length classes and by sequence identity with the authentic mtDNA for different NUMT length ranges. **a** Distribution of the size of the NUMT sequences identified in the reference pig genome (length classes are reported in bp). **b** Plots showing the Kernel density estimate of NUMT sequence identity with the authentic porcine mtDNA grouped by sequence size

Approximately 70% to 79% of the NUMT regions (detected in the different assembled genomes) consisted of a single NUMT sequence, while only 21% to 29% of NUMT regions were composed of more than one NUMT sequence. The length of the NUMT sequences ranged from 30 to 11,205 bp and the total summed length per assembled genome (from 203.14 to 352.64 kb) covered about 0.01% of the total pig nuclear genome size. Most of these sequences were shorter than 500 bp (median size of 196 to 237 bp; average size and standard deviation ranging from about 349 ± 468 bp to 400 ± 737 bp; Fig. 2a). Very similar results were found when mining the assembled genomes available for the other two Suidae species, *S. cebifrons* and *P. africanus*. Summary statistics of the detected NUMT in the mined genomes are reported in Additional file 2: Table S5. Sequence identities with the true mitochondrial DNA ranged from 56 to 100%, with the highest identity concentrated in the shortest NUMT (Fig. 2b).

### Comparative analysis of NUMT regions across assembled genome versions

**Fig. 3:**
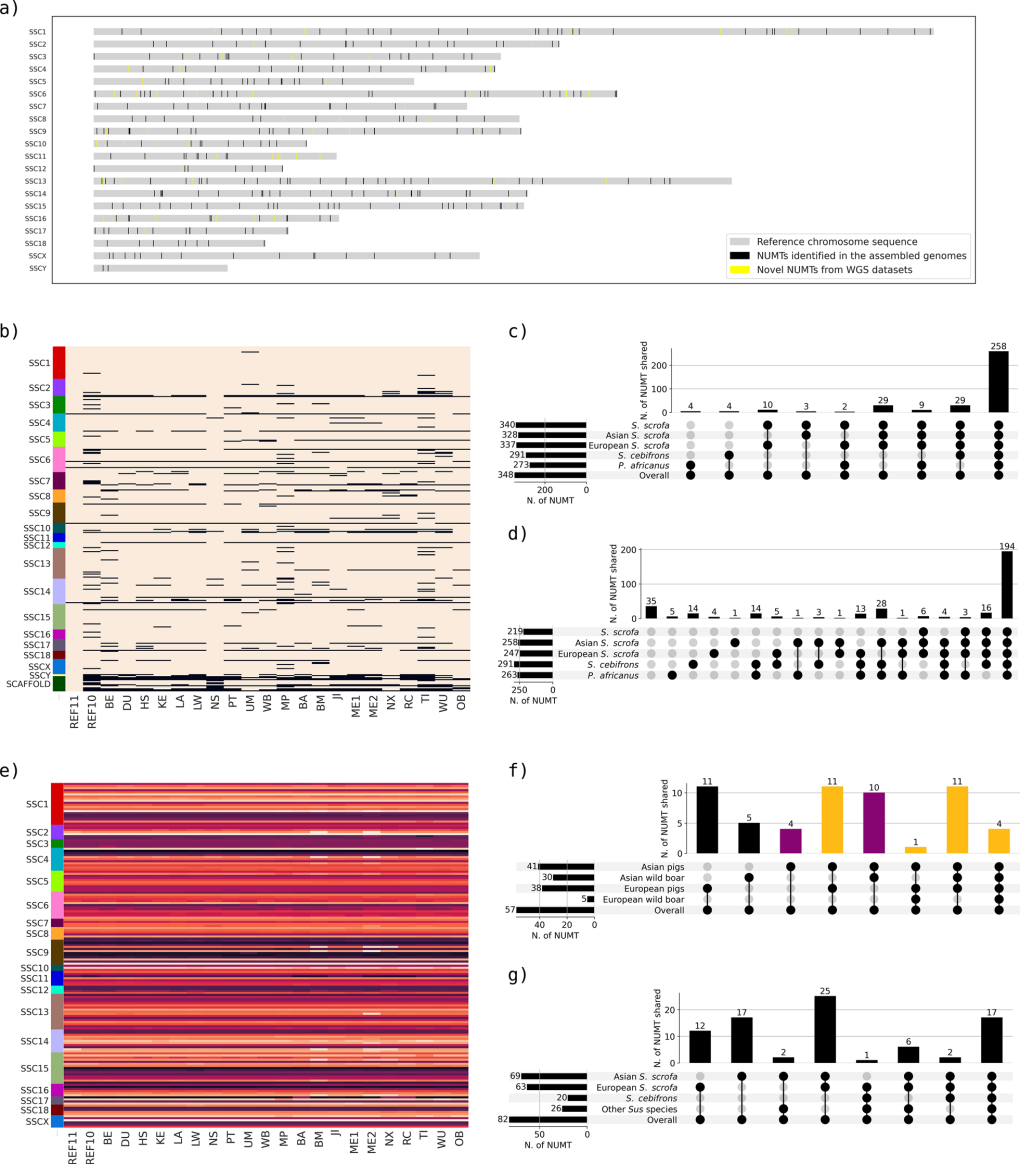
Comparative information on NUMT. **a** Map distribution of all NUMT identified in the assembled genomes and whole genome sequencing (WGS) datasets. All NUMT positions are reported based on the reference pig genome (Sscrofa11.1). On the X axis, the length of the chromosome sequence is shown in units of 100 million of bp. On the Y axis, the number of the *Sus scrofa* chromosome is reported. Grey bars represent the size and scale of the chromosomes in the reference genome; black lines represent NUMT identified in at least one of the assembled genomes analysed, and yellow lines represent novel NUMT identified in the WGS datasets. NUMT present on unmapped scaffolds are not shown. **b** Distribution of all non-redundant NUMT based on the Sscrofa11.1 chromosomes across all assembled genomes (represented by the vertical columns), including information on the presence (light brown) or absence (black) of each NUMT region (represented by horizontal rows). The first coloured column shows the reference genome chromosomes on which the NUMT were mapped. **c** UpSet plot showing all 348 NUMT regions identified in the assembled genomes grouped by species and origin (X axis) and their presence in the other species/breeds (Y axis). **d** UpSet plot showing NUMT identified in all of the assembled genomes for each group (i.e. fixed), grouped by species and origin (X axis) and their presence in the other species/breeds (Y axis). **e** Mean identity between authentic mtDNA and nuclear sequence fragments for each NUMT region shared by all assembled genomes. The colour indicates the identity percentage, ranging from the lowest (black) to the highest (light brown), increasing in brightness with the identity. The first coloured column shows the reference genome chromosomes on which the NUMT were mapped. **f** UpSet plot showing the number of all 57 novel NUMT identified in the WGS datasets of *S. scrofa* grouped by European/Asian origin and domestic breed/wild boar (X axis), with their presence in the other groups (Y axis). The yellow highlighting indicates the 27 NUMT identified in both Asian and European pigs; the purple highlighting indicates the 14 NUMT identified in Asian pigs and not in European pigs or wild boars. **g** UpSet plot showing on the X axis all 82 polymorphic NUMT in Asian or European pigs and wild boar assembled genomes or WGS datasets, *S. cebifrons* assembled genome or WGS datasets, other *Sus* species WGS datasets. *S. cebifrons* was analysed separately from the other *Sus* species as it was the only species among the considered ones with an assembled genome. The Y axis shows their presence in different species/breeds (Y axis)

Figure 3a shows the chromosome distribution of all NUMT regions detected in the assembled genomes and reported on the Sscrofa11.1 genome version. Based on Sscrofa11.1, the average distance between NUMT regions was ~ 7 Mb, with a median of ~ 5 Mb. An overview of the NUMT distributed on the pig chromosomes and detected in the various genome versions is reported in Additional file 3: Fig. S1.

#### Comparison between Sscrofa11.1 and Sscrofa10.2 pig genome versions

A comparison between the two latest versions of the *S. scrofa* reference genome revealed that Sscrofa11.1 has more NUMT sequences and NUMT regions, as well as a larger total size of NUMT sequences compared to Sscrofa10.2 (903 *vs* 679; 338 *vs* 329; 342.232 kb *vs* 258.238 kb; for the three parameters, respectively). A detailed chromosome by chromosome comparison of these parameters between these two *S. scrofa* genome versions is reported in Additional file 2: Table S7. There is a strong correlation between the number of NUMT insertions per chromosome and the chromosome size (Pearson correlation, r = 0.97, p < 0.0001). This correlation holds true for all other assembled genomes as well (data not shown).

Out of the 338 and 329 NUMT regions identified in the Sscrofa11.1 and Sscrofa10.2 genome versions, 285 (84% and 87%, respectively) were found in both genome versions, as determined by whole genome alignment data. For the remaining 53 NUMT regions only found in Sscrofa11.1 and for 44 only found in Sscrofa10.2, there was no overlap between the two genome versions.

#### Overview of NUMT detected in all assembled genomes of *Suinae* species

An overview of the distribution of NUMT regions identified in one or more assembled genomes and shared in other assembled genomes is provided in Additional file 3: Fig. S2. The overall orthologous matrix, which reports information on the number of corresponding NUMT regions identified in all mined *S. scrofa*, *S. cebifrons* and *P. africanus* assembled genomes is reported in Additional file 2: Table S8. The number of NUMT regions belonging to portions of the genomes for which no alignment with Sscrofa11.1 could be found ranged from six (Nero Siciliano assembled genome) to 50 (one of the two *P. africanus* assembled genomes) in the different assembled genomes (the detailed numbers can be found in the diagonal of Additional file 2: Table S8); three of the NUMT regions found in Sscrofa11.1 are located in scaffolds belonging to portions of the genome that could not be aligned to any of the other assembled genomes. Most of the NUMT regions identified in each assembled genome (Additional file 2: Table S5) were also detected in Sscrofa11.1: when considering only the *S. scrofa* assembled genomes, the percentage ranged from 87 to 98%; for the assembled genomes of the other Suinae species, 93% of the *S. cebifrons* regions and 87% and 89% for the two *P. africanus* genomes were orthologous to NUMT regions of Sscrofa11.1. When considering all non-redundant NUMT regions in portions of the genome that could be aligned with Sscrofa11.1, a total of 340 unique NUMT insertion events (98% also included in Sscrofa11.1) were identified from all pig assembled genomes. If *S. cebifrons* and *P. africanus* are considered in addition to *S. scrofa* genomes, a total of 348 NUMT regions have been detected in the Suinae lineage (Fig. 3b and c). Overall, of the 348 NUMT regions identified across all these assembled genomes that could be mapped on Sscrofa11.1, 219 (62%) were shared by all *S. scrofa* assembled genomes and 194 (56%) were shared by all Suinae assembled genomes (including *S. cebifrons* and *P. africanus* genomes) (Fig. 3d).

#### Putative polymorphic NUMT identified by mining assembled genomes

Five of these NUMT regions were not inserted in the Duroc pig nuclear DNA from which the Sscrofa11.1 reference genome was constructed. This is evident from the alignments with other assembled genomes, which showed gaps in Sscrofa11.1 for the entire length of the corresponding NUMT region. Therefore, they can be considered polymorphic insertion sites within this species, indicating the presence or absence of the insertion (NUMT insertions/deletions—indels). The variations could be directly detected by comparing Sscrofa11.1 with other assembled *S. scrofa* genomes. Four NUMT regions were found only in the *S. cebifrons* genome, while another four were found only in *P. africanus* genome. Therefore, their insertions likely occurred after their evolutionary divergence from a common ancestor with *S. scrofa.* Additional information on the orthologous NUMT comparison between the different assembled genomes is reported in Additional file 2: Table S9. Figure 3b summarises all NUMT regions identified in the Suinae genomes providing information on their presence or absence across assembled versions based on the corresponding chromosome positions. The information on the number of NUMT shared by the different assembled genomes is summarized in Fig. 3c. The number of NUMT found in groups of assembled genomes and their presence in other groups categorized by breed/species origin is summarized in Fig. 3d. These additional figures offer an overview of the NUMT present or absent in various versions of the assembled pig genomes, potentially indicating polymorphic NUMT (NUMT indels), segregating in different pig breeds and populations.

#### Potential biases and artifacts

Considering the information provided above, cautions should be taken declaring polymorphic NUMT. This is particularly important when the presence or absence of an insertion was observed in one assembled genome as this potential insertion or deletion (which ranges from three to 50 in the assembled genomes) could be the result of artifacts generated during the assembly processes. This is evident from the fact that not all assembled genomes contained the 22 orthologous NUMT that were also identified in *B. taurus* and *C. hircus*. These NUMT are considered ancestral, meaning they were inserted in a common ancestor of these species and should be found in all assembled genomes. However, since this is not the case (with an average presence of 95.8% in the *S. scrofa* genomes), and sequence inspection of these regions might exclude very rare mutation events within the *S. scrofa* species, it is likely that genome assemblies contain some inaccuracies leading to false positive or false negative NUMT detection.

To further evaluate the potential impact of the quality of the assembled reference genome on the detection of NUMT using the applied methods, we calculated the correlations between the contig N50 value of the pig assembled genomes that were mined (ranging from 22 kb to 51 Mb) and the number of NUMT regions, NUMT sequences and the total size of NUMT sequences detected (Additional file 2: Table S5). We found some correlation between the contig N50 values and the number of NUMT regions (Spearman’s correlation, ρ = 0.60, p = 0.002) and a highly significant correlation between the contig N50 and the number of NUMT sequences, as well as the total size of NUMT sequences (Spearman’s correlations: ρ = 0.88, p = 2.3E−8; and ρ = 0.9, p = 5.47E−9, respectively). These results suggest that the overall low quality of the assembled genome may have a negative impact on the identification of NUMT regions using the applied methods, with a particularly relevant negative effect on the number of NUMT sequences detected within the NUMT regions.

Figure 3e shows NUMT regions shared by all genomes along with the mean sequence identity of each region with the consensus mitochondrial sequence. As expected, most shared insertions tend to have the same identities across all assembled genomes (clearly indicating their common ancestral origin, making it possible to identify true orthologous regions), with the exception of some regions in the Meishan (ME2), Bama miniature (BM) and Ningxiang (NX) assembled genomes. These regions show significantly higher identities compared to the orthologous (or putative orthologous) NUMT in other genome versions. For example, the NUMT region SSC8_REGION_5, consisting of a 1537 bp sequence in the Sscrofa11.1 reference genome, had an identity of 91% in all genomes except for the ME2, BM, and NX genome versions, where the identity was 98%. The reason for this phenomenon could be either a different evolutionary rate in the Asian pig genomes after the split from the European pig (~ 1 Mya), or artifacts generated by the genome assembly procedures resulting in the integration/correction of the assembly from the authentic mitochondrial DNA sequence instead of the correct NUMT sequence. Since in other Asian pig nuclear genomes the identity is not different from that observed in the European pig nuclear genomes, around 91%, the second hypothesis seems more likely. The same can be said for the largest NUMT sequence (SSC2_REGION_14, 11,160 bp in Sscrofa11.1), which had a sequence identity of ~ 90%, except in the controversial genomes, where the identity with the true mtDNA was ~ 98%.

### Whole genome sequencing datasets provide additional information

A total of 485 whole genome sequencing (WGS) datasets from various breeds/populations and *Sus* species were mined (i) to identify additional NUMT not present in the analysed pig reference and drafted genomes, (ii) to confirm the presence of reported NUMT identified in one or more pig assembled genomes but not in all pig assembled genomes, (iii) to provide preliminary information on the frequency of polymorphic NUMT sites in certain pig populations and (iv) to gather additional information on NUMT present in the genome of other Suinae species.

#### Additional NUMT not included in the assembled genomes

After filtering the reads of the 485 analysed WGS datasets, a total of 1517 breakpoints indicating the presence of additional NUMT regions (most of them mapping to the same genomic positions), other than the 348 non-redundant NUMT regions previously described in the assembled genomes, were identified from a total of 449 WGS datasets (92%; 266 out of 302 ENA datasets; 160 out of 160 Italian pig breed datasets; and 23 out of 23 DNA pooled datasets). From these datasets, 70 unique additional NUMT regions were identified on 17 chromosomes (and thereafter referred to as novel NUMT regions). Of these, 50 of them (71%) were found only in the *S. scrofa* WGS datasets, 13 (18.5%) were present in both *S. scrofa* and other *Sus* species, and seven (10%) were exclusive to the other *Sus* species. These 70 novel NUMT regions were detected in more than one of the investigated WGS datasets (from *S. scrofa* ENA datasets: n. 52; from Italian breed datasets: n. 25; from DNA pool datasets: n. 22; from ENA datasets of other Suinae species: n. 20) and were mainly found in wild boars (n. 30), Erhualian (n. 24), Meishan (n. 21), Rongchang (n. 21) and Italian Large White (n. 20) breeds. Among the other *Sus* species, these novel NUMT regions were found in *S. barbatus* (n. 13), *S. verrucosus* (n. 13), *S. cebifrons* (n. 11) *and S. celebensis* (n. 5). The number of novel NUMT regions per *S. scrofa* WGS dataset ranged from 0 to 13, with an average of 3.03 (with sizes ranging from 51 to 2443 bp, average of 299 bp). In the WGS datasets of species other than *S. scrofa,* this number ranged from 0 to 10, with an average of 6.06 across the 16 datasets (and sizes ranging from 66 to 603 bp, with an average of 226 bp). The number of novel NUMT was partially correlated with chromosome size (Pearson correlation, r = 0.52, p = 0.03; Additional file 3: Fig. S3) and was also partly correlated with the depth of sequencing of the WGS datasets (Pearson correlation, r = 0.30, p = 8.97E−12; Additional file 3: Fig. S4). Detailed information for all novel NUMT found in WGS datasets is reported in Additional file 2: Table S10.

#### Distribution of novel NUMT in different pig breeds and *Suidae* species

Considering the 70 novel NUMT regions found only in WGS datasets, the number of WGS datasets sharing the same NUMT region ranged from 2 to 278, with a mean value of 21. The frequency of carriers of all these novel NUMT insertions divided by breeds or groups of pigs/species is reported in Additional file 2: Table S11. Additional file 3: Fig. S5 shows a heatmap of the carrier frequencies for all breeds and groups of datasets (including species and origin of the breeds). These novel NUMT regions had an overall low frequency, with some exceptions. For example, the NUMT region SSC13_REGION_34 was present in 278 WGS (57%), including 18 out of 68 Asian pig datasets (26%) from six out of 13 Asian breeds/populations, and 239 out of 347 European pig datasets (65%) from 34 out of 38 European breeds/populations, in addition to WGS from both European and Asian wild boars and all other *Sus* species except *S. celebensis*. PCR validation of this NUMT region provided a similar frequency of the insertion in the tested cosmopolitan breeds (Italian Duroc, Italian Landrace and Italian Landrace), with an overall frequency of 0.6. The polymorphic state of this NUMT was also confirmed in autochtonous Italian pig breeds (Additional file 2: Table S12). Other PCR validated polymorphic NUMT had very close insertion frequencies to those estimated from WGS datasets (Additional file 2: Tables S4, S11, S12 and S13).

None of these novel NUMT regions were observed in all Asian or in all European breed datasets. A total of 27 novel NUMT were observed in both Asian and European pig breeds/wild boars (see the sum of the yellow bars of Fig. 3f) A total of 14 NUMT regions were detected in one or more Asian population datasets (with overall carrier frequencies lower than 0.10) and in none of the European breed or European wild boar datasets (see the sum of the purple bars of Fig. 3f). On the other hand, a total of 11 NUMT regions were identified only in European pig breed/European wild boar datasets (with overall carrier frequencies lower than 0.12) and in none of the Asian population datasets (Fig. 3f, Additional file 2: Table S11). These putative specific NUMT regions of the Asian or European breeds might have been recently introgressed into one of these two lineages. Their presence was, in a few cases, observed in just one or few breeds (with one breed having a higher carrier frequency), suggesting that they emerged in one breed and then spread to other breeds due to crossbreedings that contributed to the constitution of the breed genetic pool. For example, NUMT SSC11_REGION_15 was identified only in Duroc (20% of the carriers) and Italian Duroc datasets (58.3% of the carriers), for a total of 27 out of 66 Duroc-line derived datasets, and in one out of six Iberian datasets analysed.

#### Comparison between WGS datasets and assembled genomes

We observed a moderate and significant correlation between the number of WGS datasets containing NUMT regions and the number of assembled genomes in which the corresponding NUMT region was identified (Pearson correlation, r = 0.61, p = 2.09E−36). Additional file 3: Fig. S6 reports the relationship between these two values. All NUMT regions found in more than 20 assembled genomes are also found in more than 400 WGS datasets, while NUMT found in less than 5 assembled genomes are found in a varied range of WGS datasets, from 0 to more than 400. Additional file 2: Tables S13 and S14 summarise, for all NUMT regions reported on Sscrofa11.1, the frequency of carriers of the insertion computed for all pig breeds, groups of breeds and other *Sus* species, with an indication of polymorphic NUMT derived from comparative analyses across assembled genomes and WGS datasets. From this overview, it was interesting to note that several NUMT that were always (or almost always present) in a few or all Suinae assembled genomes were also polymorphic in *S. scrofa* and viceversa. For example, among the 289 NUMT regions shared by both *S. scrofa* and *S. cebifrons* genomes, 24 had a carrier frequency in *Sus scrofa* WGS datasets lower than 97%, suggesting that these regions might be polymorphic in domestic pig populations (as 3% was estimated to be the false negative detection error rate; see below). These NUMT regions were present in a number of mined *Sus scrofa* assembled genomes that ranged from 11 to 23 out of the 23 that we investigated, further supporting the fact that these NUMT sequences are polymorphic in domestic pigs (Additional file 2: Table S14). There were some differences in the frequency of the carriers between the European and Asian pig derived WGS datasets, which were also reflected in the frequency of the presence/absence of some NUMT regions in the two groups of assembled pig genomes (Fig. 3g). For example, NUMT SSC9_REGION_22 of 648 bp was carried by all WGS datasets obtained from European pigs (99.2%) and was observed in all 13 assembled genomes of the same origin but was carried by 75% of the WGS datasets generated from Asian domestic pigs and was detected in only 4 out of 9 Asian pig assembled genomes. SSC16_REGION_13 constituted another interesting example: it was detected in the *S. cebifrons* assembled genome; it was not detected in any *S. scrofa* assembled genomes; domestic pig WGS datasets carried this NUMT with a frequency of 13.6%; the total frequency of this NUMT region in the WGS obtained from other *Sus* species was 31.2% (Additional file 2: Table S14).

Considering porcine NUMT regions that emerged in the *Sus* genus (i.e., either found in *S. scrofa* only or shared between *S. scrofa* and other *Sus* species), we identified a total of 82 polymorphic NUMT regions (i.e., NUMT with a carrier frequency lower than 97%, considering the false negative detection rate of 3% explained below). Out of these 82 NUMT regions (Fig. 3g), 57 were novel NUMT detected only in the WGS datasets, while the remaining 25 were detected in the assembled genomes.

### NUMT age estimation and phylogenetic analyses

#### Phylogenetic information

**Fig. 4:**
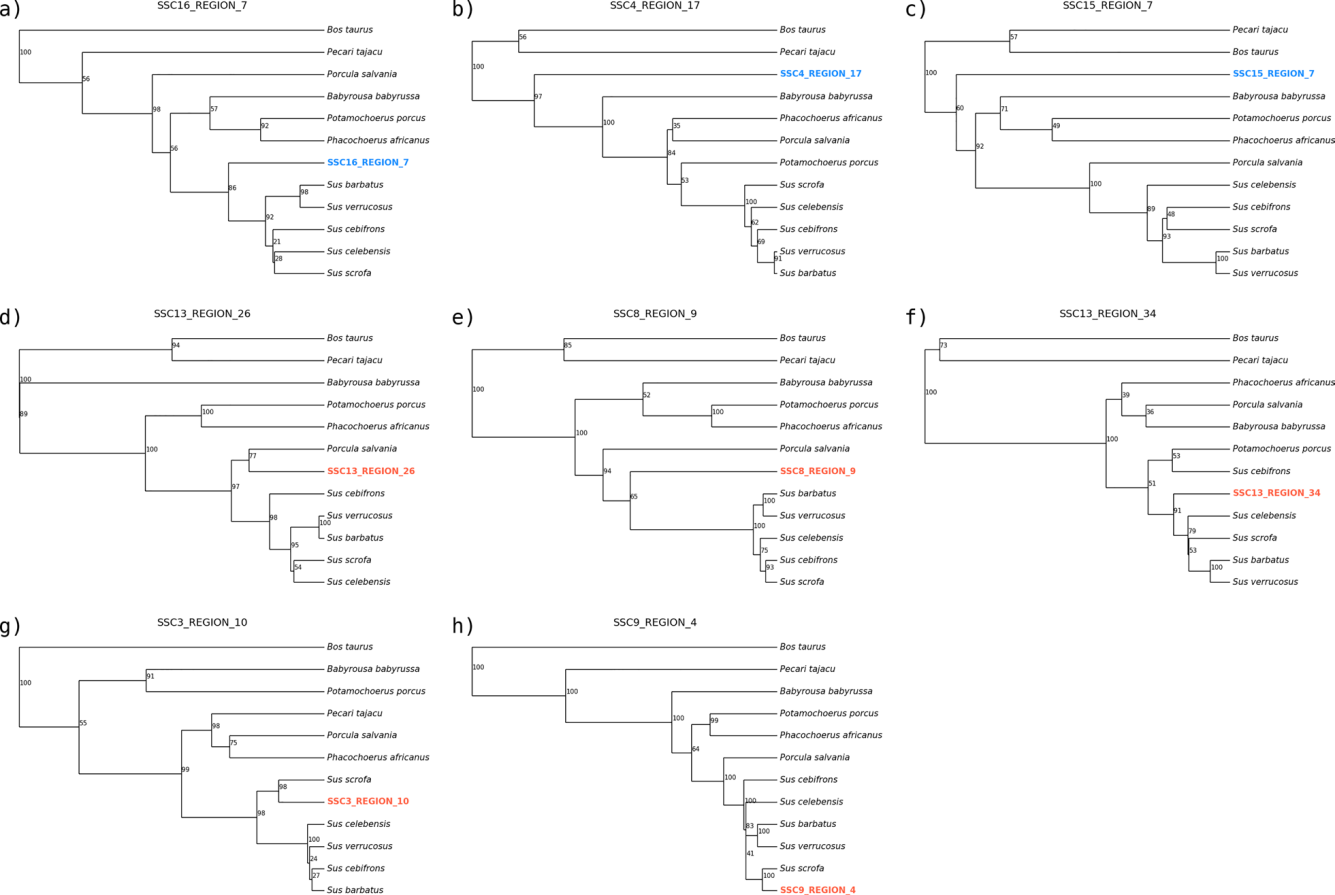
Phylogenetic trees of eight NUMT regions. The figure shows the phylogenetic tree computed for eight NUMT regions (in the subsets indicated from a to h), including the information from the corresponding region of the authentic mitochondrial DNA (mtDNA) of other Cetardiodactyla species. The NUMT are indicated in red or blue in the phylogenetic trees. The mtDNA of the other species is labelled with the scientific name written in black. Fixed NUMT (i.e. non-polymorphic NUMT) are labelled in blue: **a** SSC16_REGION_7; **b** SSC4_REGION_17; **c** SSC15_REGION_7. Polymorphic NUMT are labelled in red: **d** SSC13_REGION_26; **e** SSC8_REGION_9; **f** SSC13_REGION_34; **g** SSC3_REGION_10; **h** SSC9_REGION_4). More information on these NUMT are reported in Additional file 2: Tables S4 and S6. The posterior probability computed by BEAST is reported along each node in the tree, and ranges from 0 to 100. For each tree, the X axis represents the branch length

**Fig. 5:**
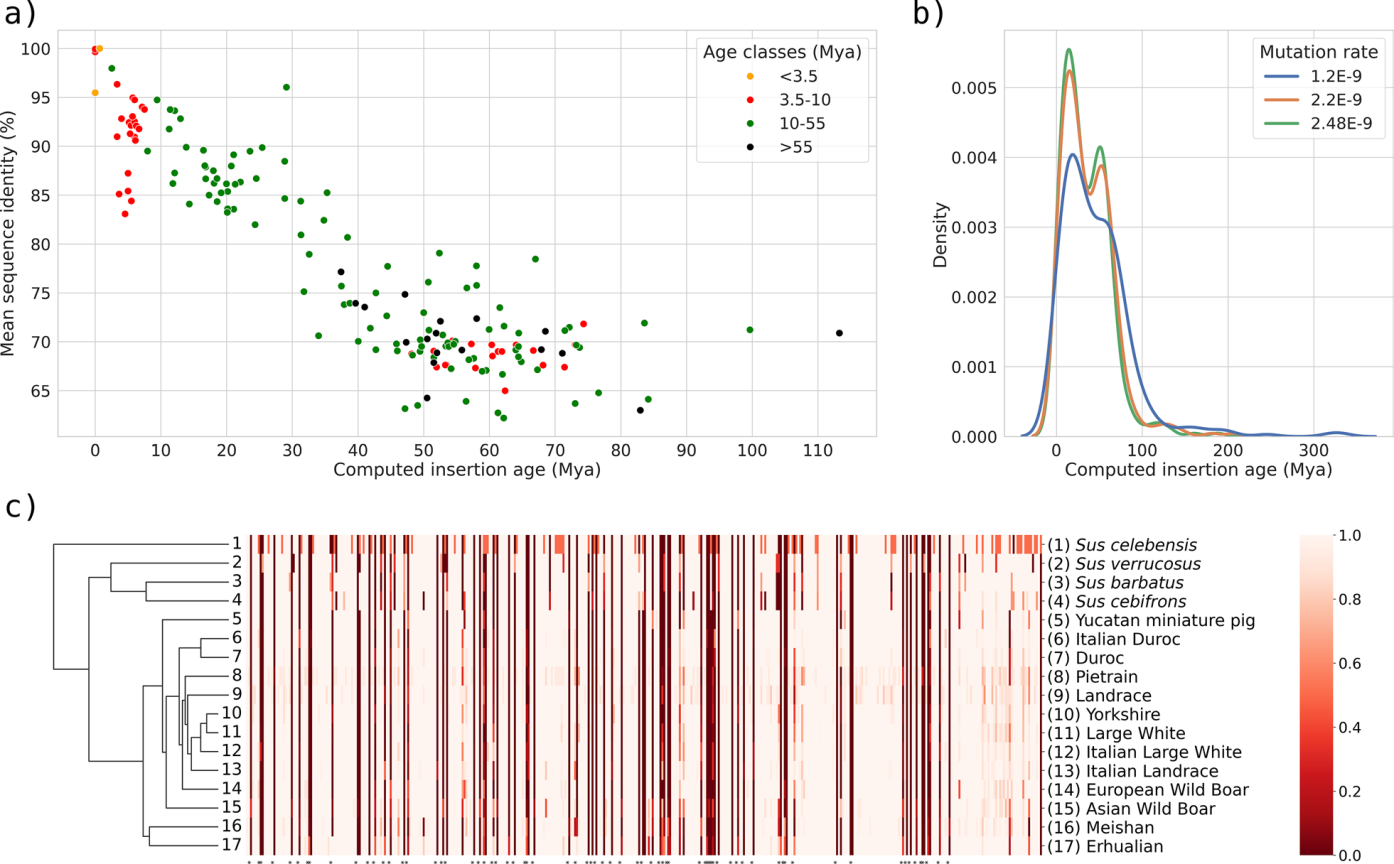
Phylogenetic information obtained from NUMT regions. **a** Estimated ages of the NUMT regions compared to their sequence identity; each point represents a different NUMT region; the age of the region is reported on the X axis; the average sequence identity between the authentic mtDNA and the nuclear NUMT sequence is reported on the Y axis. The colours of the dots represent the estimated age class of the NUMTs determined from its presence or absence in the assembled genomes and WGS datasets. An outlier NUMT region with a computed age > 200 Mya (SSC7_REGION_2) was not included in the plot. **b** Kernel density estimate of the computed NUMT region ages based on the different mutation rates. The X axis shows the computed age and the Y axis the kernel density. **c** Cluster representation derived from NUMT information obtained from WGS datasets of *S. barbatus*, *S. cebifrons*, *S. celebensis*, and *S. verrucosus* and several pig breeds and populations. The cluster includes only breeds/populations and species for which at least 10 WGS datasets were available. The numbers in the correspondence of the different tree branches are referred to the respective name on the indicated on the right of the figure. The dendrogram on the left side of the figure shows the clustering based on the UPGMA algorithm (unweighted pair group method with arithmetic mean) and the Euclidean distance metric, derived from the presence/absence and carrier frequency of all NUMT, as represented in the middle of the figure and listed in the X axis (each position represents a NUMT identified from WGS). The NUMT positions are marked with an asterisk if identified only in the WGS datasets and in none of the assembled genomes (novel NUMT). The carrier frequency for each NUMT in the corresponding breed/population or species is represented with a scale colour reported on the right of the figure, that goes from dark red (0%) to pale pink (100%). The heatmap of Additional file 2: Fig. S5 includes all precise indications of the NUMT in the X axis, that here are simplified

Phylogenetic trees were obtained for all NUMT regions (n. 178) for which a NUMT sequence was longer than 150 bp (Additional file 4: Fig. S7). Figure 4 reports the phylogenetic trees of a few NUMT that demonstrate how their estimated insertion age agrees with the phylogenetic relationships between species. Figure 5a shows the distribution of the 172 out of 178 NUMT regions for which an insertion age could be estimated using sequence identity information with the true mtDNA sequences (Additional file 2: Table S14), classified according to their estimated age window based on the presence of the corresponding ortholog NUMT in other species or only in *Sus scrofa* (defining four age classes: in *Sus scrofa* and *Bos taurus*/*Capra hircus*: > 55 Mya; in *Sus scrofa* and *P. africanus* from 55 ≥ Mya > 10; in *Sus scrofa* and other *Sus* species but not in *P. africanus*: 10 ≥ Mya > 3.5; only in *Sus scrofa* or only in other *Sus* species: ≤ 3.5 Mya). Estimation of the insertion age for the two age classes > 10 Mya was based on the average mammalian nuclear mutation rate of 2.2E–9 per site/year [72]. Nearly overlapping results for these two age classes were obtained using the mutation rate proposed by Garcia-Erill et al. [74] (mean rate of 2.48E–9 per site/year; Fig. 5b; Additional file 2: Table S14). A general agreement with the estimated age and the related classification is evident, considering the median computed age (Fig. 5a, Table 2): 101 out of 172 NUMT regions (59%) had a computed insertion age compatible with its classification defined as mentioned above. When we used the mutation rate of 1.2E–9 per site/year proposed by Zhang et al. [75], the number of concordant results decreased for the age class from 55 to 10 Mya (55 NUMT regions instead of 72 classified using the average mammalian mutation rate or 76 classified using the mean rate of Garcia-Erill et al. [74]) but increased for the age class > 55 Mya (15 NUMT regions instead of 7 or 6 using the other two alternative mutation rates, respectively; Fig. 5b; Additional file 2: Tables S14 and S15).

**Table 2:** NUMT regions divided by age classes with information on the computed insertion age

Age class	Age window (Mya)^a^	Total number of NUMT regions	Number of NUMT regions with computed age^b^	Average sequence identity^b^	Median sequence identity^b^	Average computed age^c^	Median computed age^c^
Ancestral (shared by *B. taurus/C. hircus* and *S. scrofa*)	> 55	22	19	70.94	70.89	57.40	51.89
Shared by *S. scrofa* and *P. africanus*	55–10	247	106	80.48	80.88	43.19	44.43
Shared by *S. scrofa* and other *Sus* species	10–3.5	72	45	84.29	88.94	28.31	6.25
*P. africanus* only	< 10	4	−	94.68	94.48	−	−
*S. cebifrons* only	< 3.5	4	−	92.60	91.67	−	−
*S. scrofa* only	< 3.5	57	2	97.37	100.00	0.35	0.35
Other *Sus* species only (WGS samples)	< 3.5	12	−	−	−	−	–

^a^Evolutionary time window (age class) of NUMT insertion defined according to the evolutionary relationships between the investigated species

^b^The average and median of the sequence identity (%) was computed using NUMT from the assembled genomes (i.e. with a complete sequence available). Sequences included in the Sscrofa11.1 genome were used when this genome included the NUMT

^c^The region approx. age (Mya) was computed only for NUMT with sequence length > 150 bp; some age classes did not have NUMT > 150 bp. For the two age classes 10–55 Mya and > 55 Mya, computed ages were based on the mammalian nuclear mutation rate [72]. Information on the estimated age of NUMT regions using the mutation rate proposed by Garcia-Erill et al. [74] and Zhang et al. [75] is reported in Additional file 2: Tables S14 and S15

A positive correlation was found between the estimated insertion age and the number of sequence fragments in the NUMT regions (Spearman’s correlation, ρ = 0.53, p = 6.4E−14), and between the estimated insertion age and the length of the NUMT sequences (Spearman’s correlation, ρ = 0.55, p = 8.25E−11). Therefore, longer NUMT were generally associated with more primitive insertions. However, it is possible that a few inconsistencies might arise from estimation biases due to the small size of the considered NUMT sequences. Other inconsistencies could be attributed to the loss of some NUMT in a few lineages during their evolutionary pathways, interspecies introgression (as exampled later), or misassembled problems in these regions. Some of these regions might be also subjected to selection pressures that could alter their evolutionary signature pattern.

#### Ancient NUMT

A total of 22 out of the 348 non-redundant NUMT regions described in a Suinae assembled genome (~ 6%) were also shared by *B. taurus* and/or *C. hircus* (using our stringent criteria to define orthologous NUMT; Table 2). These NUMT regions were considered ancient NUMT, which may have been inserted in a common ancestor over 55 Mya. Twenty-one out of these 22 NUMT regions are also found in both *P. africanus* assembled genomes, while one is only found in one of the two common warthog assembled genomes. The sequence identity with the *S. scrofa* mtDNA sequence for all these ancestral NUMT ranged from 63 to 77%, with an average of 71%. Their calculated average insertion age was ~ 57.4 ± 17.7 Mya, ranging from ~ 37.4 to ~ 113.3 Mya. The discrepancies in age insertion estimation, in comparison to what would be expected (> 55 Mya) for a few NUMT may be due to biases from the short inserted NUMT fragments (as previously mentioned) and the not constant mutation rates over evolutionary ages and genome regions. These NUMT regions were also found in 469 (97%) to 485 (100%) WGS datasets, including from 15 to 16 out of 16 other *Sus* species WGS datasets. This supports the hypothesis that all these NUMT insertions occurred in a common ancestor of all considered species and were subsequently inherited by all descendant lineages. The minor discrepancies reported for a few WGS datasets (3%) can be empirically considered, in these cases, as a measure of the false negative detection rate of the methodology used to identify NUMT from WGS datasets based on short sequencing reads (which we also used above to define a threshold useful to declare NUMT as polymorphic). The 106 NUMT regions with sequences longer than 150 bp that were shared by both *S. scrofa* and *P. africanus* had an average insertion age of ~ 43 ± 26 Mya (with a range from ~ 2.5 to 206 Mya). Of these, 31 NUMT regions had an estimated insertion age > 55 Mya but were not detected in *B. taurus* and *C. hircus*. This suggests that they could have been lost from an ancestral genome common only to both cattle and goat. The oldest region of ~ 206 Mya is an outlier of 178 bp with a sequence identity of 59%, likely been degraded by several disruptive mutation events. This NUMT may be considered an ancient NUMT that was lost or undetectable in *B taurus* and *C. hircus.* It is important to note that the stringent criteria used to define orthologous NUMT across distant species may have also led to increased false negative results in some instances. Three NUMT were found to have a computed age < 10 Mya (SSC4_REGION_3, ~ 8 Mya; SSC14_REGION_2, ~ 2.5 Mya; SSC15_REGION_21, ~ 9.4 Mya), which contradicts their presumed insertion time of more than 10 Mya, even though all three were found in at least 480 (99%) WGS datasets, including those of other *Sus* species. The mutation rate of these three NUMT may have been slowed down due to some evolutionary constraints or interspecies/intertaxa introgressions over a long evolutionary period or the short-inserted sequences (ranging from 197 to 237 bp) could have skewed the age estimation.

#### Suinae specific NUMT

A total of 45 NUMT regions for which it was possible to estimate an insertion age were found in both *S. scrofa* and in the other *Sus* species using both assembled genomes and WGS information (Table 2). Their absence from the *P. africanus* assembled genomes implies that the NUMT insertion might have occurred in a common ancestor to all *Sus* species but not to the *Phacochoerus* genus (between ~ 3.5 and 10 Mya). The median age computed for these regions was ~ 6.25 Mya (which is generally consistent with the classified insertion range of most of these NUMT). However, nineteen of these NUMT have a low sequence identity with the authentic mitochondrial sequence (< 80%), suggesting an insertion predating *Sus* speciation. The longest NUMT sequence found in this study (11,160 bp, SSC2_REGION_14), shared by both *S. scrofa* and *S. cebifrons* genomes, was estimated to be inserted ~ 6.1 Mya, consistent with the hypothesis of its insertion in a common ancestor between the two species, after the divergence from *P. africanus*. The phylogenetic tree (see Additional file 4: Figure S7) confirmed the insertion age of this NUMT, after the divergence with the *Phacochoerus* genus and before the divergence between *Porcula* and *Sus* species (~ 4 Mya).

Other NUMT were identified only in one Suinae species: *P. africanus* (four NUMT, for none of which an age could be computed due to their short fragment size), *S. cebifrons* (three other NUMT, again too short to compute their age) and *S. scrofa* (seven NUMT regions). Of these *S. scrofa* NUMT, an insertion age could be computed for two of them, which were found to be integrated at 0 and 0.69 Mya, respectively.

We also estimated the insertion age of 16 polymorphic NUMT regions in *S. scrofa*, as determined by combining (i) information from assembled genomes, (ii) the carrier frequency from WGS datasets and (iii) the PCR validation for some of them (Additional file 2: Table S12). For six NUMT regions with an estimated insertion age > 10 Mya, a plausible explanation for their polymorphic status in *Sus scrofa* might be due to deletions of the corresponding region or other mutational events that prevent their correct identification in some animals/populations. For eight other NUMT regions with an estimated insertion age between ~ 3.5 and 10 Mya (i.e., before the separation of the *S. scrofa* lineage from the other *Sus* species), it is possible that their polymorphic status in domestic pigs results from introgression events between the *S. scrofa* lineage and other *Sus* lineages after the speciation time, further supporting findings by Schiavo et al. [14]. In all of these cases, the NUMT insertion borders are perfectly conserved within *S. scrofa* and the other *Sus* species. The frequency of genomes carrying these NUMT ranged from 0.67 to 1 and from 0.26 to 1 in the European and Asian pig derived WGS datasets, respectively. For example, the polymorphic NUMT SSC9_REGION_22, already mentioned above, may have been inserted into the nuclear genome around 6 Mya. This NUMT was polymorphic in Asian pig breeds (carried by 75% of the Asian pig WGS datasets), it was present in the *S. cebifrons* assembled genome and in all other WGS datasets derived from *Sus* species different from *S. scrofa*. A similar polymorphic profile was observed for another NUMT (SSC8_REGION_9) with the same estimated insertion age (Additional file 2: Table S14). Other polymorphic NUMT in *S. scrofa* (SSC3_REGION_17, estimated insertion age ~ 4 Mya; SSC5_REGION_13, estimated insertion age ~ 7.1 Mya; SSC13_REGION_26, estimated insertion age ~ 6.25 Mya; and SSC13_REGION_34, estimated insertion age ~ 3.33 Mya, close to the extreme border time of this class of NUMT), with a carrier frequency in pig populations derived from WGS datasets ranging from 0.57 to 0.96 and not present in the *S. cebifrons* assembled genome, were also polymorphic in one or more of the other *Sus* species, as determined from the respective WGS datasets. Two polymorphic NUMT in *S. scrofa* (SSC8_REGION_3 and SSC9_REGION_4) were not detected in the assembled genome of *S. cebifrons* but were identified in all WGS datasets derived from *S. barbatus*, *S. cebifrons*, *S. celebensis* and *S. verrucosus*. Their estimated insertion ages (~ 3.33 and ~ 0 Mya, respectively) suggest that they may have been recently included in the *Sus* lineage, even though they might be classified among the group of NUMT that occurred between ~ 10 to 3.5 Mya. In these two cases, as well as for the three other NUMT previously mentioned that were not fixed in all *Sus* species different from *S. scrofa*, it is not clear what the direction of the introgression would be.

Two other NUMT (SSC5_REGION_12 and SSC11_REGION_15), only reported in *S. scrofa* genomes, for which we also estimated the insertion age in agreement with their classification among the recently occurred NUMT (< 3.5 Mya), had carrier frequencies determined from all WGS datasets of 0.55 and 0.06, respectively. The first had a low frequency in the Asian WGS datasets (0.07) whereas the second was not observed in pigs of Asian origin (Additional file 2: Table S14).

Taking into consideration the frequency of NUMT identified from WGS datasets (Additional file 3: Fig. S5) in *S. barbatus*, *S. cebifrons*, *S. celebensis*, and *S. verrucosus* as well as in different pig breeds/populations (for which at least 10 datasets were available, to obtain reliable population genomic analyses) we clustered the obtained information. The dendrogram we obtained matched the phylogenetic relationships between the considered groups/taxa (Fig. 5c): *S. barbatus*, *S. cebifrons*, *S. celebensis*, and *S. verrucosus* were clearly separated from the other *Sus scrofa* groups; the Asian breeds were clustered together; and the European breeds populations were grouped together; closely related breeds (for example, Large White, Italian Large White and Yorkshire; Landrace and Italian Landrace; Duroc and Italian Duroc) were also branched together. The position of the Yucatan miniature pig was in the middle between European and Asian breeds and close to Asian wild boars. Even though all of these taxa share some polymorphic NUMT (segregating within and across species), the NUMT information obtained from WGS datasets was able to provide valuable phylogenetic information based solely on the genetic diversity described by NUMT. The only exception was for the Asian wild boar datasets, which clustered with the Yucatan miniature pig datasets. However, both groups were still branched together and separated from the European breed/population branches.

### Features of NUMT sequences and NUMT insertion regions

**Fig. 6:**
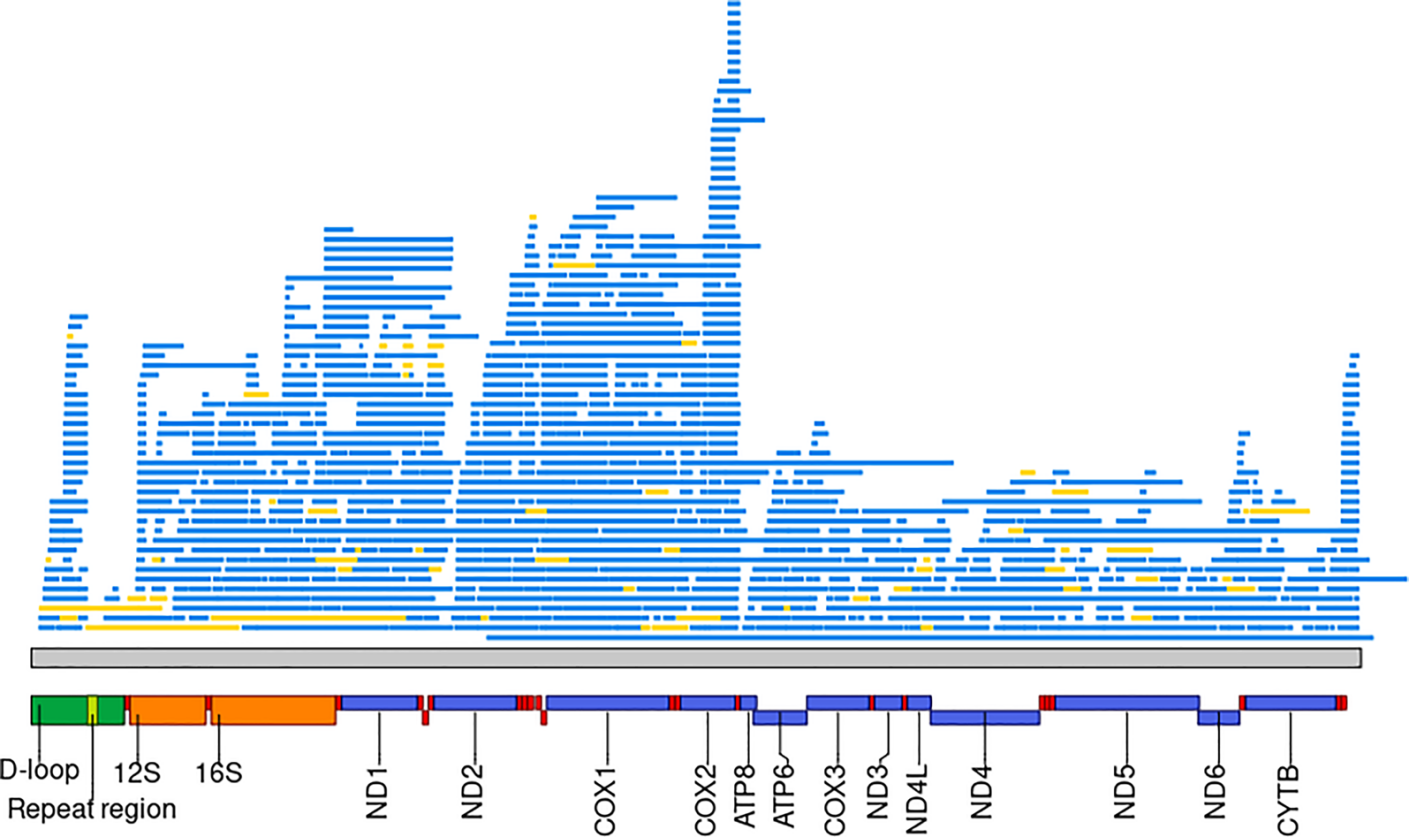
Alignments of the NUMT fragments identified in* Sus scrofa* with their linearized and annotated authentic mitochondrial DNA (mtDNA). Each line represents a NUMT sequence identified in the assembled genomes (blue) or in the WGS datasets (yellow). The mtDNA is annotated with the corresponding genes and D-loop regions

Figure 6 displays the distribution of NUMT hits derived from all *S. scrofa* investigated genomes (all assembled genomes and from all WGS datasets) on the linearized authentic *S. scrofa* mitochondrial genome sequence annotated for the mtDNA genes and regions. This distribution is also reported for *S. cebifrons* and *P. africanus*, including hits from their corresponding NUMT (Additional file 3: Fig. S8). In all these three species, every mtDNA region was covered by more than one NUMT. Excluding the D-loop, which has more complicated alignments due to a highly variable number of repeats, the first part of the mtDNA (from *16S rRNA* to *COX2* genes) was, generally, more covered by NUMT than the second part (from *ATP8* to *CYTB* genes). The most covered regions included *ND1*, *COX1* and *COX2* genes.

Annotation of the 418 NUMT regions identified (348 NUMT from the assembled genomes plus the 70 novel NUMT from WGS datasets) indicated that 50% of them (n. 209) overlapped with a gene feature in the nuclear genome, all of which were introns. The remaining regions were inserted into intergenic positions. None of the sequences overlapped with gene exons. Therefore, it appears that the detected NUMT could not directly impact on any gene function or structure. Further verification is needed to determine if their integration could affect regulatory regions potentially impacting gene expression, splicing, or silencing. The general distribution across different porcine genome regions is similar to what was observed in humans [12], with 42% of NUMT located in introns and 43% in intergenic regions.

All 418 NUMT insertion flanking regions overlapped with repeated elements. The most frequent repeats are SINE (327 regions), LINE (293 regions), tRNA and RNA containing repeats (286 regions). All information about the annotated NUMT insertion regions is available in Additional file 2: Table S16. To test whether NUMT regions are preferentially located within regions containing repetitive elements, Fisher’s exact test was performed, using the number of nucleotides overlapping repeats in the NUMT insertion flanking sequences compared to the whole reference genome. NUMT insertions appeared to be preferentially located within repeated regions (p < 0.000001), in particular in the regions that had the following repeats: LINE, SINE, Simple repeat, LTR, DNA, tRNA, Satellite, snRNA, rRNA, RC, and RNA. No significant difference in GC content was observed between flanking regions and the whole reference genome (41.5% *vs* 41.6%).

Additional file 5: Table S17 reports the GFF file annotation of the Sscrofa11.1 genome version with the NUMT identified in this study, which could be useful for other investigations.

## Discussion

In this study we used various sources of sequence information to depict a landscape of NUMT insertions not only in the *Sus scrofa* genomes but also in the genomes of other related species of the Suidae family (subfamily Suinae). The availability of numerous assembled genomes and hundreds of WGS datasets obtained from many different breeds and populations provided an opportunity to characterise pig NUMT in greater depth than has been done in any other livestock species to date. We primarily referred to the concept of NUMT region, defined as a single putative insertion event of mtDNA sequences into the nuclear genome, which can provide phylogenetically relevant information. The majority of NUMT regions (70–79%, as identified from the assembled genomes) were determined by a single NUMT sequence, simplifying the interpretation of the data. It is quite interesting that some NUMT regions, after being inserted into the nuclear genome, experienced further mutational events that fragmented the original mtDNA sequences. Older NUMT insertions, as it might be expected, tended to be more fragmented into multiple sequences. This could be also due to the degradation of some sequences that prevented them from being read as one continuous fragment. Additionally, there was a positive correlation between the length of NUMT sequences and their age, suggesting that longer mtDNA sequences had a higher likelihood of being integrated into more primitive genomes. It is also possible that waves of NUMT integrations over evolutionary time had different characteristics. As far as we know, these aspects have not been thoroughly explored in other studies on NUMT in mammalian genomes. It will be interesting to further explore this question in other species and lineages to obtain a more detailed analysis of NUMT integration.

The occurrence of NUMT is a very rare event, still ongoing [56]. It leaves an evolutionarily relevant signature in the lineages that derived from the ancestral genome where the unique insertion event occurred, providing a link of common evolutionary origin between genomes sharing orthologous NUMT [11, 13, 15]. These signatures, therefore, span a very broad evolutionary time window, that can be traced back until it is possible to detect mtDNA sequences not completely degraded by the time-proportional mutation rate: the level of identity between the NUMT sequence and the corresponding modern mtDNA sequence can provide useful information to estimate the insertion approximate age. It is however important to consider that mtDNA sequences integrated into the nuclear genome have adapted their evolutionary rate to the slower pace of evolution of the genome where they are inserted compared to the faster pace of the authentic mtDNA. Therefore, to obtain an approximate insertion age of NUMT into the porcine genome we applied a combination of information that considered the different mutation rates between nuclear and mitochondrial genomes. We also examined the presence of orthologous NUMT in various species, tracing back to a common ancestral lineage. The farthest boundary of this lineage was determined using the cattle and goat genomes. Additional intermediate time points were identified using the genomes of the warthog (*P. africanus*) and Visayan warty pig (*S. cebifrons*), which are phylogenetically closer to the *S. scrofa* genome, as these species belong to the same Suinae subfamily. Including the genomes of two species from the Bovidae family (*B. taurus* and *C. hircus*) provided valuable information on ancient orthologous NUMT that may have been lost in one species or the other, or that could not be identified due to genome assembly issues in one of these two Bovidae reference genomes.

Only NUMT sequences > 150 bp were used to estimate an insertion age. For shorter sequences, the class of insertion age was defined based on their presence or absence in other genomes mentioned. It is evident that the size limit chosen may not contain enough informative differences, or could contain by chance too many differences, leading to unreliable insertion age estimation. This poses the risk of underestimating or overestimating the NUMT age, resulting in assigning a younger or older) age than the correct age. Two mutation rates [72, 74] that we used to estimate the age of NUMT insertions for the two age classes > 10 Mya produced nearly identical results. The mutation rate proposed by Zhang et al. [75] was probably not suitable for NUMT inserted up to 55 Mya but it worked well for older NUMT regions. These findings suggest that different mutation rates should be taken into account when studying a broad evolutionary time frame. Additionally, another challenge we encountered was the limited availability of few assembled genomes for orthologous validation of insertion sites across closely related species to *S. scrofa*. The quality of these assembled genomes is still not very high, which introduces another potential bias in the identification of orthologous NUMT across species. Additionally, orthologous analyses become somewhat complicated when considering evolutionarily distant genomes, especially for NUMT inserted in intergenic regions (the most common group of NUMT detected in the pig genome), which might have, in our study, restricted the number of NUMT that we confidently considered orthologous between cattle/goat and pigs. For these reasons we did not evaluate in more detail if we could detect constant or differing rates of insertion throughout the evolutionary history of the *S. scrofa* genome or between Suinae species. Although our results were not specifically obtained at exploring this issue in depth, and considering the limitations mentioned above, we observed a few groups of more frequent waves of NUMT insertion (Fig. 5a, b and Additional file 2: Table S14). One wave embraced the last 25 Mya (late Oligocene) and a more ancient pattern dating back over 40 Mya (early Eocene). Further research is needed to provide a more comprehensive description of the insertion frequency of NUMT over time, incorporating data from additional genomes of the Suidae family, similar to studies conducted on the primate lineage [58].

A total of 418 putative unique integration events of mtDNA derived sequences (determined by the total number of NUMT regions that could be mapped on the Sscrofa11.1 genome version) were detected. When summing up the sequence of all detected NUMT, the authentic mtDNA was covered multiple times. The integration sites into the pig genome were more frequent within repeated regions, as previously reported in our previous study on pigs [14], and in agreement with findings in other mammalian genomes [3, 12, 34, 36, 38]. The methodologies used, including (i) bidirectional pairwise LAST analyses [52, 61], to compare assembled genomes, and (ii) WGS datasets to retrieve additional NUMT not included in the genomes of the animals from which the assembled genomes were obtained [55, 56], provided a comprehensive list of cross-validated NUMT present in the pig germline. Considering the strict criteria we used to call NUMT from WGS datasets, putative NUMT regions detected in just one of these datasets may have been originated from unique integration events that occurred in the somatic cell lines of the sequenced animals (from which DNA was extracted [56]). However, the stringent filtering criteria applied to WGS datasets could potentially lead to false negative results. To address this, we implemented an internal control method based on ancient NUMT to estimate the rate of false negative results, which could impact the identification of polymorphic NUMT. The in vitro validation, that we carried out for some of the polymorphic NUMT, confirmed what was derived from WGS dataset analyses, indicating that the applied pipeline correctly identified carriers of the inserted NUMT. The depth of sequencing was, as expected, correlated, at least in part, to the possibility of correctly detecting NUMT from WGS datasets. Moreover, the quality of the assembled genomes might also be relevant to correctly report NUMT: it seems that some NUMT could not be correctly included in the genomes with low N50, reducing the number of detected NUMT regions. In addition, some local assembly problems could reduce the total number of NUMT sequences in the genomes of lower quality. This is also evident from the differences detected in the two latest porcine reference genome versions: Sscrofa11.1, which has a better assembled quality than Sscrofa10.2, contained more NUMT. Some putative NUMT included at the extreme ends of unassembled scaffolds in all assembled genomes could not be considered as true integrations with high confidence, because it was not possible to identify the corresponding orthologous sequence in the other assembled genomes. Most of the putative low-confidence NUMT included in unassembled scaffolds are likely derived from assembly artifacts caused by chimeric sequences containing authentic mtDNA fragments. Other problems were detected in three assembled genomes (Meishan, Bama miniature and Ningxiang) where orthologous NUMT did not share the same sequence identity as the corresponding mtDNA sequences reported in all other assembled genomes. This bias likely resulted from the correction procedures applied during assembly, rather than different evolutionary rates of the same NUMT in different breeds. The routine use of long sequencing read technologies for genome resequencing and the construction of pangenomes will improve the identification of NUMT, offering optimal solutions to solve the problems mentioned above.

By combining information from several assembled pig genomes, WGS datasets, and in vitro confirmation through PCR analyses, it was possible to validate that polymorphic NUMT, characterised by the presence or absence of integration in the genomes (i.e., indels), segregate in different pig populations. Approximately 7% (n. 25) of the NUMT regions included in the assembled genomes were found to be polymorphic. This estimation of the number of polymorphic NUMT is close to what was reported, on average, across six primate species [58], with a higher level in humans (18%) where more information could be retrieved. Therefore, it might be possible to identify additional polymorphic NUMT in pigs by adding other assembled genomes and WGS datasets to this analysis.

The porcine polymorphic NUMT were integrated either after the *Sus* species differentiation (< 3.5 Mya), or before this evolutionary divergence time (> 3.5 Mya), as defined by classical phylogenetic approaches, without considering the reticulate history resulting from interspecific admixture [65]. Some of the NUMT older than 3.5 Mya were also observed in the assembled *S. cebifrons* genome and/or detected in other *Sus* species. Their estimated insertion age and presence in other *Sus* species may indicate that they were introduced into the *S. scrofa* genome from interspecific admixture that might have occurred quite recently [14, 66]. Some other polymorphic NUMT were quite recent as their identity with true mtDNA was 100% or close to it. It is also interesting to note that in several cases the same polymorphic NUMT segregated in both European and Asian breeds, suggesting that their occurrences predated the separation between these two groups or that subsequent human mediated introgression between European and Chinese domestic pools might have contributed to shaping the within breed genetic variability of the breeds originating in these two geographic areas [77]. Human-mediated crossbreeding may have played a role in the spread of ancient or more recent (> 3.5 Mya or < 3.5 Mya) polymorphic NUMT regions in the two groups of pig breeds, as the same polymorphic NUMT (of the two types) segregated in Asian and European breeds. Our analysis of porcine WGS datasets was also able to preliminarily estimate the frequency of indel alleles of polymorphic NUMT in many different pig breeds and populations (based on the carrier status of the sequenced pigs), providing additional information on this type of DNA variants at the population genomic level. We did not differentiate between homozygous carriers and heterozygous carriers due to the limited depth of sequencing in some datasets (close to 10 ×) that did not provide enough reads to confidentially distinguish these two genotypes. However, this analysis revealed that certain polymorphic NUMT alleles are more prevalent in either European or Asian domesticated pools, shedding light on the micro-evolutionary relationships between these populations, whose complex domestication trajectory [78] could be also traced back, or even refined, including NUMT information. The identification of the inserted allele of polymorphic NUMT regions only in closely related pig breeds can further demonstrate the genetic closeness or relationships of these populations.

According to evolutionary information inferred from the presence or absence of certain polymorphic NUMT, it appears that interspecies exchange of nuclear genomic material may have occurred throughout the entire *Sus* genus. These results further confirm that the evolutionary history of *Sus* nuclear genomes can be better explained by interspecies reticulate exchange of genomic material resulting from numerous events of interspecies admixture [79]. All extant species of the *Sus* genus, with the exception of *S. scrofa* (originally found across a wide area spanning many Euro-Asian regions) are now only found in the biodiversity hotspot of the Island Southeast Asia (ISEA) region (*S. barbatus* is found in Borneo, the Malay Peninsula and Sumatra; S. *cebifrons* and *S. philippensis* in the Philippines; *S. celebensis* in Sulawesi *S. verrucosus* in Java and Bawean). In this region, intertaxa crossbreeding events may have taken place [79], contributing to the creation of unique intragenus specific combinations whose signature can also be observed in NUMT regions. One of the potential uses of NUMT is to disentangle the evolutionary relationships not only between *Sus* species but also within the Suidae family. This usefulness is derived from the fact that NUMT contains information from both mtDNA and nuclear genomes, thus integrating (but also complicating) the phylogenetic signatures from these two types of genomes. Therefore, it is suggested that NUMT should be further investigated to improve the reconstruction of the intricate evolutionary relationships and events within the Suidae family. It is also interesting to note that the overall profile of polymorphic and fixed NUMT identified in *S. scrofa* and other *Sus* species, along with information on the approximated frequency of indel alleles in these groups, allowed for the clustering of different pig breeds, *S. barbatus*, *S. cebifrons*, *S. celebensis*, and *S. verrucosus* in a meaningful way that resembled (at least partially) their evolutionary relationships and genetic distance.

As far as we know, polymorphic NUMT have been reported only in few other mammals till now: for example, in humans [12, 56, 58, 80], other primates [15, 58] and horse [41]. However, it is possible to expect that more detailed analyses of WGS datasets can obtain information on polymorphic NUMT in several other species, providing micro-evolutionary insights (e.g., at the species, subspecies and breed levels) that might be quite difficult to be reconstructed with other approaches, in particular when reticulate introgression could have contributed to shaping the modern genomes, like in the case of pig breeds [77, 78].

Polymorphic NUMT segregating within domestic pig populations could mark the corresponding pig genome regions that may reveal unique features or effects on economically important traits, stemming from the ancient origin of the surrounding haplotypes. Other studies in pigs have already shown significant associations between certain haplotypes, resulting from interspecies introgression or preserved as ancestral trans-species polymorphic sites, and relevant traits. For example, Ai et al. [81] reported that a large (14-Mb) region with a low recombination rate on porcine chromosome X, identified in Chinese pig breeds carries a haplotype conferring environmental adaptation that may have been introgressed from an extinct *Sus* species. The ancestral trans-species *O* allele of the *ABO* gene, segregating in various pig populations and in *S. cebifrons* [82, 83], was associated with a distinct gut microbiota profile [82]. Exploring polymorphic NUMT regions, especially those with older insertion age, could provide insights into why their alleles continue to segregate in highly selected pig breeds and whether polymorphic haplotype regions encompassing these NUMT integration sites are associated with economically important traits.

## Conclusions

This study provided a first comprehensive analysis of NUMT included in the *Sus scrofa* genome, compared to NUMT detected in the genomes of other species of the order Cetartiodactyla (two ruminants: cattle and goat; and several species of the Suinae subfamily, more closely related to *S. scrofa*), to obtain a hierarchical evolutionary time window useful for tracking and estimating their integration age. Polymorphic NUMT insertions older or more recent than 3.5 Mya segregate in many different Asian and European pig breeds. This suggests that these events contributed to shaping the pig genome structure at different evolutionary ages and that the process is still ongoing. The analyses of more assembled genomes and other WGS are expected to increase the number of NUMT that have colonised the nuclear genome, adding other information for reconstructing the events that contributed to the domestication of this species and then the breed constitution processes.

## Supplementary Information

**Additional file 1: Table S1.** Information on the assembled nuclear genomes investigated in this study. **Table S2.** Information on the WGS datasets analysed in this study. **Table S3.** Information on the mitochondrial genomes used in this study. **Table S4.** Information on the NUMT regions that were validated by PCR analyses.

**Additional file 2: Table S5.** Information on the NUMT identified in the assembled genomes. **Table S6.** List of all NUMT sequences identified in the assembled genomes with LAST alignment information. **Table S7.** Comparison at the chromosome/scaffold level between NUMT identified in Sscrofa11.1 and Sscrofa10.2 reference genomes. **Table S8.** Matrix of orthologous NUMT regions over all Suinae investigated assembled nuclear genomes. The matrix below the diagonal contains the number of NUMT regions orthologous between the two genomes; the diagonal of the matrix reports the total number of regions found in the genome and the number of regions belonging to portions of the genome not aligned with Sscrofa11.1. The matrix above the diagonal contains the number of regions from genome A not found in genome B and vice versa. The acronyms of the reference genomes are explained in Additional file 2 Table S5. **Table S9.** List of all non-redundant NUMT regions found in the assembled genomes with presence/absence in all other assembled genomes. NUMT regions that could not be reconducted to a position in Sscrofa11.1 were not included. The first six columns contain the unique NUMT region ID and its coordinates in Sscrofa11.1. For NUMT regions absent in Sscrofa11.1 the coordinates of the insertion point are reported instead. The remaining columns refer to the information for the unique NUMT region in the assembled genomes: the reported information for each genome, when available, were the ID and coordinates of the corresponding NUMT region, along with its status, defined as follows: CORRESPONDING, if the genome contains the NUMT region; COMPATIBLE SEQUENCE, if the genome aligns with Sscrofa11.1 on the NUMT region coordinates, and the corresponding sequence in the other genome was not annotated as NUMT by the NUMT discovery pipeline; PRIVATE, if the alignment between the assembled genome and Sscrofa11.1 contains gaps corresponding to the NUMT region coordinates in one of the two genomes, meaning that the NUMT insertion happened only in one of the two genomes, and the NUMT can be therefore considered as polymorphic. Each status column contains the information for Sscrofa11.1 on the left side and the other genome on the right side. **Table S10.** List of all novel NUMT identified by mining WGS datasets. Breakpoints belonging to the same NUMT insertion are grouped with the same NUMT region ID. **Table S11.** Frequency of the carriers of novel NUMT regions estimated from WGS datasets within breed/population and species. For each NUMT region, number of WGS datasets and their percentageof the carriers is reported. **Table S12.** Genotype information obtained by PCR analyses of some polymorphic NUMT in several pig breeds and populations. The number of pigs having the three genotypesis reported. **Table S13.** Frequency of the carriers of all non-redundant NUMT regions found in assembled genomes estimated from WGS datasets within breed/populations and species. For each NUMT region, number of WGS datasets and their percentage of the carriers is reported. **Table S14.** Information on NUMT derived from the assembled genomes and WGS datasets with age estimation, presence/absence in the assembled genomes and WGS datasets divided by species and geographic origin of the pigs. **Table S15.** Comparative age estimation of NUMT regions assigned to the age classes 10–55 Mya and > 55 Mya, based on different mutation rates. **Table S16.** Annotation of the genomic regions where the NUMT regions are inserted.

**Additional file 3: Figure S1.** Number of NUMT regions, divided by chromosome, identified in the investigated pig assembled genomes, mapped on the Sscrofa11.1 chromosomes. Acronyms of the assembled genomes are explained in Additional file 1: Table S1. **Figure S2.** Distribution of NUMT regions identified in one or more assembled genomes and shared in two or more assembled genomes. The X axis reports the number of assembled genomes in which a NUMT region was identified. Numbers range from 0 to 25. Acronyms of the assembled genomes are explained in Additional file 1: Table S1. **Figure S3.** Graphical representation of the relationships between Sscrofa11.1 chromosome length and number of novel NUMTs found on the chromosome. Chromosome length is shown in in Mb. The regression line shows the trend along with its confidence. **Figure S4.** Graphical representation of the relationships between WGS dataset sequencing depth and number of novel NUMT found in the dataset. Dots represent the investigated WGS datasets. **Figure S5.** Heatmap showing the frequencies of the NUMT in different breeds/populations/species as estimated from WGS datasets. The Y axis reports all breeds/populations/species from which WGS datasets were used to estimate the frequency of the carriers of the inserted NUMT into the genome. Information is divided for pig breeds/populations and species or grouped considering all datasets or according to the origin of the pig breeds or investigated species. WGS datasets derived from DNA pools are indicated with an asterisk on the Y axis. Novel NUMT regions identified only in WGS datasets are indicated with an asterisk on the X axis. The frequency is reported with the scale colour, going from dark red to pale pink. Figure 9 shows a clusterisation of the species, breeds and populations based on the same information, considering only groups where at least 10 WGS datasets were analysed. **Figure S6.** Relationship between the number of assembled genomes sharing a NUMT region and the number of WGS datasets sharing the NUMT region. **Figure S8.** Alignments of the NUMT fragments identified in the *Sus cebifrons *and *Phacochoerus africanus* reference genomes with their corresponding linearized and annotated true mitochondrial DNA. In subfigure a) each line represents a NUMT fragment identified in the *S. cebifrons* assembled genome or in a *S. cebifrons* WGS datasets; in subfigure b) each line represents a NUMT fragment identified in the *P. africanus* assembled genomes. The mtDNA of both species is annotated with the corresponding genes and D-loop regions.

**Additional file 4: Figure S7.** Phylogenetic trees were obtained for all NUMT regions for which a NUMT sequence was longer than 150 bp. Each tree was computed from the longest NUMT sequence in the region. The title of the tree reports the name of the NUMT region. The X axis of each tree represents the branch length. Polymorphic NUMT regions are colored red, and fixed NUMT regions are colored blue. The NUMT nuclear sequence is compared with the corresponding sequences in the mtDNA of several species. The posterior probability computed by BEAST is reported along each node in the tree, and ranges from 0 to 100.

**Additional file 5: Table S17.** Annotation of the Sscrofa11.1 genome version with NUMT regions in GFF format. The nine columns in the file are based on the standard format for a GFF file. The second column indicates the NUMT source; the third column indicates the feature type name.

## Data Availability

Data generated during this study are included in this published article and its supplementary information files. The annotation of the Sscrofa11.1 genome version with NUMT regions in GFF format is included as Additional file 5: Table S17. Whole genome sequencing datasets generated from Italian Duroc, Italian Landrace and Italian Large White during the current study are not publicly available due restriction in their use but are available from the corresponding author on reasonable request, after signing an agreement on their use. Workflows and scripts developed for this study are freely available on GitHub (https://github.com/Animal-and-Food-Genomics-Group/comprehensive-atlas-of-numts-pig).

## Abbreviations

mtDNA: Mitochondrial DNA
Mya: Millions of years ago
NUMT: Nuclear sequence of mitochondrial origin
WGS: Whole genome sequencing
