# Supplementary material for: A comprehensive atlas of nuclear sequences of mitochondrial origin (NUMT) inserted into the pig genome
Source: Genet Sel Evol. 2024 Sep 16;56:64. doi: 10.1186/s12711-024-00930-6 (PMC11403998; doi:10.1186/s12711-024-00930-6)
Supplement: Supplementary file 1 — Additional file 1: Table S1. Information on the assembled nuclear genomes investigated in this study. Table S2. Information on the WGS datasets analysed in this study. Table S3. Information on the mitochondrial genomes used in this study. Table S4. Information on the NUMT regions that were validated by PCR analyses. [file 12711_2024_930_MOESM1_ESM.docx]

Additional file 1

Supporting information

**A comprehensive atlas of nuclear sequences of mitochondrial origin (NUMT) inserted into the pig genome**

Matteo Bolner, Samuele Bovo, Mohamad Ballan, Giuseppina Schiavo, Valeria Taurisano, Anisa Ribani, Francesca Bertolini and Luca Fontanesi

**Table of content**

**Additional file 1: Table S1.** Information on the assembled nuclear genomes investigated in this study.

**Additional file 1: Table S2.** Information on the WGS datasets analysed in this study.

**Additional file 1: Table S3.** Information on the mitochondrial genomes used in this study.

**Table S1. Information on the assembled nuclear genomes investigated in this study.**

| **Abbreviation** | **Assembly name** | **Species** | **Breed/ Species common name** | **NCBI Accession ID** | **Genome sequence length (bp)** | **Assembly level** | **Contig N50** | **Pig breed geographic origins** |
| --- | --- | --- | --- | --- | --- | --- | --- | --- |
| REF11 | Sscrofa11.1 | *Sus scrofa* | Duroc | GCF_000003025.6 | 2501895775 | Chromosome | 48231277 | European |
| REF10 | Sscrofa10.2 | *Sus scrofa* | Duroc | GCF_000003025.5 | 2808509378 | Chromosome | 69503 | European |
| BE | Berkshire_pig_v1 | *Sus scrofa* | Berkshire | GCA_001700575.1 | 2434706773 | Scaffold | 94651 | European |
| DU | Ninghe_Sus_1 | *Sus scrofa* | Duroc | GCA_015776825.1 | 2458240772 | Contig | 4265198 | European |
| HS | Hampshire_pig_v1 | *Sus scrofa* | Hampshire | GCA_001700165.1 | 2437109612 | Scaffold | 102417 | European |
| KE | CAU-K | *Sus scrofa* | Kenyan domestic pig | GCA_019290145.1 | 2444968461 | Scaffold | 223518 | European |
| LA | Landrace_pig_v1 | *Sus scrofa* | Landrace | GCA_001700215.1 | 2440981886 | Scaffold | 88142 | European |
| LW | Large_White_v1 | *Sus scrofa* | Large White | GCA_001700135.1 | 2457907103 | Scaffold | 88831 | European |
| NS | NSME_pig_1.2 | *Sus scrofa* | Nero Siciliano | GCA_006511355.2 | 2479314267 | Scaffold | 594997 | European |
| OB | ASM2471841v1 | *Sus scrofa* | Ossabaw Island hog | GCA_024718415.1 | 2455664950 | Chromosome | 8942329 | European |
| PT | Pietrain_pig_v1 | *Sus scrofa* | Pietrain | GCA_001700255.1 | 2438316299 | Scaffold | 80611 | European |
| UM | USMARCv1.0 | *Sus scrofa* | USMARC Crossbreed | GCA_002844635.1 | 2755421422 | Scaffold | 6372407 | European |
| WB | ASM2165605v1 | *Sus scrofa* | Wild Boar | GCA_021656055.1 | 2457973704 | Scaffold | 157950 | European |
| MP | SscrofaMinipig | *Sus scrofa* | Ellegaard Gottingen Minipig | GCA_000331475.1 | 2358017222 | Scaffold | 22008 | Mixed (Crossbred) |
| BA | Bamei_pig_v1 | *Sus scrofa* | Bamei | GCA_001700235.1 | 2460755741 | Scaffold | 70893 | Asian |
| BM | ASM764409v1 | *Sus scrofa* | Bama Miniature Pig | GCA_007644095.1 | 2491053062 | Chromosome | 1009524 | Asian |
| JI | Jinhua_pig_v1 | *Sus scrofa* | Jinhua | GCA_001700295.1 | 2453702738 | Chromosome | 95227 | Asian |
| ME_1 | Meishan_pig_v1 | *Sus scrofa* | Meishan | GCA_001700195.1 | 2467495771 | Chromosome | 63263 | Asian |
| ME_2 | ASM1795798v1 | *Sus scrofa* | Meishan | GCA_017957985.1 | 2510037796 | Scaffold | 51011532 | Asian |
| NX | ASM2056790v1 | *Sus scrofa* | Ningxiang | GCA_020567905.1 | 2440945073 | Scaffold | 26066865 | Asian |
| RC | Rongchang_pig_v1 | *Sus scrofa* | Rongchang | GCA_001700155.1 | 2459027728 | Chromosome | 79093 | Asian |
| TI | Tibetan_Pig_v2 | *Sus scrofa* | Tibetan | GCA_000472085.2 | 2437742524 | Chromosome | 57199 | Asian |
| WU | minipig_v1.0 | *Sus scrofa* | Wuzhisan | GCA_000325925.2 | 2508912457 | Chromosome | 31939 | Asian |
| SCE | Sus_cebifrons.v1 | *Sus cebifrons* | Visayan warty pig | GCA_905335845.1 | 2459270091 | Scaffold | 159795 | / |
| PA_1 | ROS_Pafr_v1 | *Phacochoerus africanus* | Warthog | GCA_016906955.1 | 2435083091 | Chromosome | 10602503 | / |
| PA_2 | CAU-W | *Phacochoerus africanus* | Warthog | GCA_019331625.1 | 2416652813 | Scaffold | 212097 | / |
| BTA | ARS-UCD1.3 | *Bos taurus* | Cattle | GCA_002263795.3 | 2711209831 | Chromosome | 25896116 | / |
| CHI | ARS1.2 | *Capra hircus* | Goat | GCA_001704415.2 | 2922617086 | Chromosome | 26244591 | / |

**Table S2. Information on the WGS datasets analysed in this study.**

| **Progressive no.** | **Dataset ID** | **Breed/population** | **Origin of the dataset** | **Sequencing depth** | **Species** | **Breed origin** |
| --- | --- | --- | --- | --- | --- | --- |
| 1 | SS_100 | Italian Duroc | This study | 24.17 | *Sus scrofa* | Europe |
| 2 | SS_101 | Italian Duroc | This study | 24.12 | *Sus scrofa* | Europe |
| 3 | SS_102 | Italian Duroc | This study | 24.30 | *Sus scrofa* | Europe |
| 4 | SS_103 | Italian Duroc | This study | 24.46 | *Sus scrofa* | Europe |
| 5 | SS_104 | Italian Duroc | This study | 24.26 | *Sus scrofa* | Europe |
| 6 | SS_105 | Italian Duroc | This study | 24.16 | *Sus scrofa* | Europe |
| 7 | SS_106 | Italian Duroc | This study | 24.36 | *Sus scrofa* | Europe |
| 8 | SS_107 | Italian Duroc | This study | 24.05 | *Sus scrofa* | Europe |
| 9 | SS_111 | Italian Duroc | This study | 24.08 | *Sus scrofa* | Europe |
| 10 | SS_113 | Italian Duroc | This study | 24.27 | *Sus scrofa* | Europe |
| 11 | SS_114 | Italian Duroc | This study | 24.16 | *Sus scrofa* | Europe |
| 12 | SS_126 | Italian Duroc | This study | 24.09 | *Sus scrofa* | Europe |
| 13 | SS_58 | Italian Duroc | This study | 24.22 | *Sus scrofa* | Europe |
| 14 | SS_59 | Italian Duroc | This study | 24.28 | *Sus scrofa* | Europe |
| 15 | SS_60 | Italian Duroc | This study | 24.01 | *Sus scrofa* | Europe |
| 16 | SS_61 | Italian Duroc | This study | 24.34 | *Sus scrofa* | Europe |
| 17 | SS_66 | Italian Duroc | This study | 24.55 | *Sus scrofa* | Europe |
| 18 | SS_67 | Italian Duroc | This study | 24.25 | *Sus scrofa* | Europe |
| 19 | SS_68 | Italian Duroc | This study | 23.99 | *Sus scrofa* | Europe |
| 20 | SS_69 | Italian Duroc | This study | 24.04 | *Sus scrofa* | Europe |
| 21 | SS_70 | Italian Duroc | This study | 24.26 | *Sus scrofa* | Europe |
| 22 | SS_71 | Italian Duroc | This study | 24.22 | *Sus scrofa* | Europe |
| 23 | SS_72 | Italian Duroc | This study | 23.96 | *Sus scrofa* | Europe |
| 24 | SS_74 | Italian Duroc | This study | 24.03 | *Sus scrofa* | Europe |
| 25 | SS_75 | Italian Duroc | This study | 23.80 | *Sus scrofa* | Europe |
| 26 | SS_76 | Italian Duroc | This study | 24.18 | *Sus scrofa* | Europe |
| 27 | SS_79 | Italian Duroc | This study | 23.27 | *Sus scrofa* | Europe |
| 28 | SS_80 | Italian Duroc | This study | 23.34 | *Sus scrofa* | Europe |
| 29 | SS_81 | Italian Duroc | This study | 23.46 | *Sus scrofa* | Europe |
| 30 | SS_82 | Italian Duroc | This study | 23.87 | *Sus scrofa* | Europe |
| 31 | SS_94 | Italian Duroc | This study | 23.71 | *Sus scrofa* | Europe |
| 32 | SS_96 | Italian Duroc | This study | 24.23 | *Sus scrofa* | Europe |
| 33 | SS_97 | Italian Duroc | This study | 24.47 | *Sus scrofa* | Europe |
| 34 | SS_98 | Italian Duroc | This study | 24.23 | *Sus scrofa* | Europe |
| 35 | SS_99 | Italian Duroc | This study | 23.90 | *Sus scrofa* | Europe |
| 36 | SS_10 | Italian Landrace | This study | 22.40 | *Sus scrofa* | Europe |
| 37 | SS_11 | Italian Landrace | This study | 24.06 | *Sus scrofa* | Europe |
| 38 | SS_12 | Italian Landrace | This study | 24.65 | *Sus scrofa* | Europe |
| 39 | SS_13 | Italian Landrace | This study | 22.13 | *Sus scrofa* | Europe |
| 40 | SS_14 | Italian Landrace | This study | 24.26 | *Sus scrofa* | Europe |
| 41 | SS_15 | Italian Landrace | This study | 24.35 | *Sus scrofa* | Europe |
| 42 | SS_16 | Italian Landrace | This study | 24.44 | *Sus scrofa* | Europe |
| 43 | SS_17 | Italian Landrace | This study | 24.15 | *Sus scrofa* | Europe |
| 44 | SS_18 | Italian Landrace | This study | 24.37 | *Sus scrofa* | Europe |
| 45 | SS_19 | Italian Landrace | This study | 24.26 | *Sus scrofa* | Europe |
| 46 | SS_20 | Italian Landrace | This study | 24.26 | *Sus scrofa* | Europe |
| 47 | SS_21 | Italian Landrace | This study | 20.38 | *Sus scrofa* | Europe |
| 48 | SS_22 | Italian Landrace | This study | 24.51 | *Sus scrofa* | Europe |
| 49 | SS_23 | Italian Landrace | This study | 24.42 | *Sus scrofa* | Europe |
| 50 | SS_24 | Italian Landrace | This study | 24.48 | *Sus scrofa* | Europe |
| 51 | SS_25 | Italian Landrace | This study | 24.11 | *Sus scrofa* | Europe |
| 52 | SS_26 | Italian Landrace | This study | 24.30 | *Sus scrofa* | Europe |
| 53 | SS_27 | Italian Landrace | This study | 24.43 | *Sus scrofa* | Europe |
| 54 | SS_28 | Italian Landrace | This study | 24.51 | *Sus scrofa* | Europe |
| 55 | SS_29 | Italian Landrace | This study | 24.24 | *Sus scrofa* | Europe |
| 56 | SS_2 | Italian Landrace | This study | 24.20 | *Sus scrofa* | Europe |
| 57 | SS_30 | Italian Landrace | This study | 24.36 | *Sus scrofa* | Europe |
| 58 | SS_31 | Italian Landrace | This study | 24.24 | *Sus scrofa* | Europe |
| 59 | SS_32 | Italian Landrace | This study | 24.43 | *Sus scrofa* | Europe |
| 60 | SS_33 | Italian Landrace | This study | 24.07 | *Sus scrofa* | Europe |
| 61 | SS_34 | Italian Landrace | This study | 24.07 | *Sus scrofa* | Europe |
| 62 | SS_35 | Italian Landrace | This study | 24.04 | *Sus scrofa* | Europe |
| 63 | SS_36 | Italian Landrace | This study | 24.09 | *Sus scrofa* | Europe |
| 64 | SS_3 | Italian Landrace | This study | 24.56 | *Sus scrofa* | Europe |
| 65 | SS_4 | Italian Landrace | This study | 24.20 | *Sus scrofa* | Europe |
| 66 | SS_5 | Italian Landrace | This study | 23.86 | *Sus scrofa* | Europe |
| 67 | SS_6 | Italian Landrace | This study | 24.38 | *Sus scrofa* | Europe |
| 68 | SS_7 | Italian Landrace | This study | 24.71 | *Sus scrofa* | Europe |
| 69 | SS_8 | Italian Landrace | This study | 24.18 | *Sus scrofa* | Europe |
| 70 | SS_9 | Italian Landrace | This study | 24.38 | *Sus scrofa* | Europe |
| 71 | SS_108 | Italian Large White | This study | 23.98 | *Sus scrofa* | Europe |
| 72 | SS_109 | Italian Large White | This study | 24.08 | *Sus scrofa* | Europe |
| 73 | SS_110 | Italian Large White | This study | 24.16 | *Sus scrofa* | Europe |
| 74 | SS_112 | Italian Large White | This study | 24.24 | *Sus scrofa* | Europe |
| 75 | SS_115 | Italian Large White | This study | 24.25 | *Sus scrofa* | Europe |
| 76 | SS_116 | Italian Large White | This study | 24.45 | *Sus scrofa* | Europe |
| 77 | SS_118 | Italian Large White | This study | 24.06 | *Sus scrofa* | Europe |
| 78 | SS_119 | Italian Large White | This study | 24.62 | *Sus scrofa* | Europe |
| 79 | SS_120 | Italian Large White | This study | 24.55 | *Sus scrofa* | Europe |
| 80 | SS_121 | Italian Large White | This study | 24.12 | *Sus scrofa* | Europe |
| 81 | SS_124 | Italian Large White | This study | 24.19 | *Sus scrofa* | Europe |
| 82 | SS_37 | Italian Large White | This study | 24.33 | *Sus scrofa* | Europe |
| 83 | SS_38 | Italian Large White | This study | 24.09 | *Sus scrofa* | Europe |
| 84 | SS_39 | Italian Large White | This study | 24.33 | *Sus scrofa* | Europe |
| 85 | SS_40 | Italian Large White | This study | 24.45 | *Sus scrofa* | Europe |
| 86 | SS_41 | Italian Large White | This study | 24.31 | *Sus scrofa* | Europe |
| 87 | SS_42 | Italian Large White | This study | 24.30 | *Sus scrofa* | Europe |
| 88 | SS_43 | Italian Large White | This study | 24.47 | *Sus scrofa* | Europe |
| 89 | SS_45 | Italian Large White | This study | 24.40 | *Sus scrofa* | Europe |
| 90 | SS_46 | Italian Large White | This study | 23.61 | *Sus scrofa* | Europe |
| 91 | SS_47 | Italian Large White | This study | 24.24 | *Sus scrofa* | Europe |
| 92 | SS_48 | Italian Large White | This study | 24.24 | *Sus scrofa* | Europe |
| 93 | SS_49 | Italian Large White | This study | 24.16 | *Sus scrofa* | Europe |
| 94 | SS_50 | Italian Large White | This study | 24.18 | *Sus scrofa* | Europe |
| 95 | SS_51 | Italian Large White | This study | 24.22 | *Sus scrofa* | Europe |
| 96 | SS_52 | Italian Large White | This study | 24.39 | *Sus scrofa* | Europe |
| 97 | SS_53 | Italian Large White | This study | 24.42 | *Sus scrofa* | Europe |
| 98 | SS_54 | Italian Large White | This study | 24.27 | *Sus scrofa* | Europe |
| 99 | SS_56 | Italian Large White | This study | 24.20 | *Sus scrofa* | Europe |
| 100 | SS_57 | Italian Large White | This study | 24.25 | *Sus scrofa* | Europe |
| 101 | SS_62 | Italian Large White | This study | 24.06 | *Sus scrofa* | Europe |
| 102 | SS_63 | Italian Large White | This study | 24.09 | *Sus scrofa* | Europe |
| 103 | SS_64 | Italian Large White | This study | 23.06 | *Sus scrofa* | Europe |
| 104 | SS_65 | Italian Large White | This study | 24.47 | *Sus scrofa* | Europe |
| 105 | SS_73 | Italian Large White | This study | 23.13 | *Sus scrofa* | Europe |
| 106 | SS_77 | Italian Large White | This study | 23.91 | *Sus scrofa* | Europe |
| 107 | SS_78 | Italian Large White | This study | 23.32 | *Sus scrofa* | Europe |
| 108 | SS_83 | Italian Large White | This study | 24.16 | *Sus scrofa* | Europe |
| 109 | SS_84 | Italian Large White | This study | 24.09 | *Sus scrofa* | Europe |
| 110 | SS_85 | Italian Large White | This study | 24.34 | *Sus scrofa* | Europe |
| 111 | SS_86 | Italian Large White | This study | 24.28 | *Sus scrofa* | Europe |
| 112 | SS_87 | Italian Large White | This study | 24.27 | *Sus scrofa* | Europe |
| 113 | SS_88 | Italian Large White | This study | 24.34 | *Sus scrofa* | Europe |
| 114 | SS_89 | Italian Large White | This study | 24.36 | *Sus scrofa* | Europe |
| 115 | SS_90 | Italian Large White | This study | 24.00 | *Sus scrofa* | Europe |
| 116 | SS_91 | Italian Large White | This study | 24.22 | *Sus scrofa* | Europe |
| 117 | SS_92 | Italian Large White | This study | 24.21 | *Sus scrofa* | Europe |
| 118 | AA10 | Italian Large White | This study | 22.39 | *Sus scrofa* | Europe |
| 119 | AA11 | Italian Large White | This study | 22.50 | *Sus scrofa* | Europe |
| 120 | AA13 | Italian Large White | This study | 22.68 | *Sus scrofa* | Europe |
| 121 | AA14 | Italian Large White | This study | 22.18 | *Sus scrofa* | Europe |
| 122 | AA17 | Italian Large White | This study | 22.32 | *Sus scrofa* | Europe |
| 123 | AA1 | Italian Large White | This study | 22.44 | *Sus scrofa* | Europe |
| 124 | AA30 | Italian Large White | This study | 22.70 | *Sus scrofa* | Europe |
| 125 | AA3 | Italian Large White | This study | 22.43 | *Sus scrofa* | Europe |
| 126 | AA4 | Italian Large White | This study | 22.54 | *Sus scrofa* | Europe |
| 127 | AA5 | Italian Large White | This study | 22.45 | *Sus scrofa* | Europe |
| 128 | AA6 | Italian Large White | This study | 22.26 | *Sus scrofa* | Europe |
| 129 | AA9 | Italian Large White | This study | 22.09 | *Sus scrofa* | Europe |
| 130 | AB10 | Italian Large White | This study | 22.32 | *Sus scrofa* | Europe |
| 131 | AB16 | Italian Large White | This study | 22.50 | *Sus scrofa* | Europe |
| 132 | AB19 | Italian Large White | This study | 22.58 | *Sus scrofa* | Europe |
| 133 | AB1 | Italian Large White | This study | 22.42 | *Sus scrofa* | Europe |
| 134 | AB20 | Italian Large White | This study | 22.59 | *Sus scrofa* | Europe |
| 135 | AB21 | Italian Large White | This study | 22.76 | *Sus scrofa* | Europe |
| 136 | AB27 | Italian Large White | This study | 22.25 | *Sus scrofa* | Europe |
| 137 | AB2 | Italian Large White | This study | 22.38 | *Sus scrofa* | Europe |
| 138 | AB3 | Italian Large White | This study | 22.59 | *Sus scrofa* | Europe |
| 139 | AB4 | Italian Large White | This study | 22.33 | *Sus scrofa* | Europe |
| 140 | AB5 | Italian Large White | This study | 22.59 | *Sus scrofa* | Europe |
| 141 | AB6 | Italian Large White | This study | 22.40 | *Sus scrofa* | Europe |
| 142 | AB7 | Italian Large White | This study | 22.80 | *Sus scrofa* | Europe |
| 143 | W30 | Italian Large White | This study | 22.04 | *Sus scrofa* | Europe |
| 144 | W36 | Italian Large White | This study | 22.58 | *Sus scrofa* | Europe |
| 145 | W38 | Italian Large White | This study | 22.29 | *Sus scrofa* | Europe |
| 146 | W55 | Italian Large White | This study | 22.34 | *Sus scrofa* | Europe |
| 147 | Y10 | Italian Large White | This study | 20.83 | *Sus scrofa* | Europe |
| 148 | Y11 | Italian Large White | This study | 22.12 | *Sus scrofa* | Europe |
| 149 | Y12 | Italian Large White | This study | 22.19 | *Sus scrofa* | Europe |
| 150 | Y13 | Italian Large White | This study | 22.40 | *Sus scrofa* | Europe |
| 151 | Y17 | Italian Large White | This study | 22.51 | *Sus scrofa* | Europe |
| 152 | Y23 | Italian Large White | This study | 22.08 | *Sus scrofa* | Europe |
| 153 | Y27 | Italian Large White | This study | 22.39 | *Sus scrofa* | Europe |
| 154 | Y2 | Italian Large White | This study | 22.15 | *Sus scrofa* | Europe |
| 155 | Y39 | Italian Large White | This study | 22.28 | *Sus scrofa* | Europe |
| 156 | Y3 | Italian Large White | This study | 19.44 | *Sus scrofa* | Europe |
| 157 | Y40 | Italian Large White | This study | 22.11 | *Sus scrofa* | Europe |
| 158 | Y4 | Italian Large White | This study | 21.69 | *Sus scrofa* | Europe |
| 159 | Y5 | Italian Large White | This study | 22.56 | *Sus scrofa* | Europe |
| 160 | Y7 | Italian Large White | This study | 22.19 | *Sus scrofa* | Europe |
| 161 | 1 | Cinta Senese | DNA pool. [45] | 42.28 | *Sus scrofa* | Europe |
| 162 | 2 | Alentejano | DNA pool. [45] | 41.84 | *Sus scrofa* | Europe |
| 163 | 3 | Apulo Calabrese | DNA pool. [45] | 41.98 | *Sus scrofa* | Europe |
| 164 | 4 | Mangulica | DNA pool. [45] | 40.94 | *Sus scrofa* | Europe |
| 165 | 5 | Black Slavonian | DNA pool. [45] | 40.48 | *Sus scrofa* | Europe |
| 166 | 6 | Casertana | DNA pool. [45] | 43.47 | *Sus scrofa* | Europe |
| 167 | 7 | Mora Romagnola | DNA pool. [45] | 41.08 | *Sus scrofa* | Europe |
| 168 | 8 | Nero Siciliano | DNA pool. [45] | 38.79 | *Sus scrofa* | Europe |
| 169 | 9 | Krskopolje | DNA pool. [45] | 40.66 | *Sus scrofa* | Europe |
| 170 | 10 | Negre Mallorquì | DNA pool. [45] | 41.80 | *Sus scrofa* | Europe |
| 171 | 11 | Gascon | DNA pool. [45] | 41.00 | *Sus scrofa* | Europe |
| 172 | 12 | Basque | DNA pool. [45] | 39.44 | *Sus scrofa* | Europe |
| 173 | 13 | Bisaro | DNA pool. [45] | 42.33 | *Sus scrofa* | Europe |
| 174 | 14 | Lietuvos Baltosios | DNA pool. [45] | 41.50 | *Sus scrofa* | Europe |
| 175 | 15 | Lietuvos Vietines | DNA pool. [45] | 41.88 | *Sus scrofa* | Europe |
| 176 | 16 | Turopolje | DNA pool. [45] | 42.49 | *Sus scrofa* | Europe |
| 177 | 17 | Schwäbisch Hällisches | DNA pool. [45] | 42.58 | *Sus scrofa* | Europe |
| 178 | 18 | Moravka | DNA pool. [45] | 42.17 | *Sus scrofa* | Europe |
| 179 | 19 | Sarda | DNA pool. [45] | 44.19 | *Sus scrofa* | Europe |
| 180 | 20 | Italian Large White | DNA pool. [45] | 45.12 | *Sus scrofa* | Europe |
| 181 | 21 | Italian Large White | DNA pool. [45] | 44.23 | *Sus scrofa* | Europe |
| 182 | 22 | Italian Duroc | DNA pool. [45] | 41.80 | *Sus scrofa* | Europe |
| 183 | 23 | European Wild Boar | DNA pool. [45] | 11.73 | *Sus scrofa* | Europe |
| 184 | SAMEA1557393 | *Sus celebensis* | ENA | 17.60 | *Sus celebensis* | / |
| 185 | SAMEA3375684 | *Sus cebifrons* | ENA | 37.05 | *Sus cebifrons* | / |
| 186 | SAMEA3375685 | *Sus cebifrons* | ENA | 12.51 | *Sus cebifrons* | / |
| 187 | SAMEA3375686 | *Sus cebifrons* | ENA | 11.88 | *Sus cebifrons* | / |
| 188 | SAMEA3375688 | *Sus cebifrons* | ENA | 14.40 | *Sus cebifrons* | / |
| 189 | SAMEA3375689 | *Sus cebifrons* | ENA | 13.96 | *Sus cebifrons* | / |
| 190 | SAMEA3375690 | *Sus cebifrons* | ENA | 12.28 | *Sus cebifrons* | / |
| 191 | SAMEA3376936 | Pietrain | ENA | 13.23 | *Sus scrofa* | Europe |
| 192 | SAMEA3376937 | Pietrain | ENA | 13.20 | *Sus scrofa* | Europe |
| 193 | SAMEA3376938 | Pietrain | ENA | 13.75 | *Sus scrofa* | Europe |
| 194 | SAMEA3376939 | Pietrain | ENA | 14.73 | *Sus scrofa* | Europe |
| 195 | SAMEA3376940 | Pietrain | ENA | 10.80 | *Sus scrofa* | Europe |
| 196 | SAMEA3376941 | Pietrain | ENA | 11.93 | *Sus scrofa* | Europe |
| 197 | SAMEA3376942 | Pietrain | ENA | 11.16 | *Sus scrofa* | Europe |
| 198 | SAMEA3376943 | Pietrain | ENA | 11.26 | *Sus scrofa* | Europe |
| 199 | SAMEA3376944 | Pietrain | ENA | 10.99 | *Sus scrofa* | Europe |
| 200 | SAMEA3497792 | *Sus verrucosus* | ENA | 17.97 | *Sus verrucosus* | / |
| 201 | SAMEA3497793 | Jinhua | ENA | 10.87 | *Sus scrofa* | Asia |
| 202 | SAMEA3497794 | Jinhua | ENA | 10.93 | *Sus scrofa* | Asia |
| 203 | SAMEA3497795 | Jiangquhai | ENA | 13.78 | *Sus scrofa* | Asia |
| 204 | SAMEA3497798 | Leping Spotted | ENA | 11.59 | *Sus scrofa* | Asia |
| 205 | SAMEA3497799 | Leping Spotted | ENA | 14.51 | *Sus scrofa* | Asia |
| 206 | SAMEA3497800 | Meishan | ENA | 11.77 | *Sus scrofa* | Asia |
| 207 | SAMEA3497803 | Meishan | ENA | 10.74 | *Sus scrofa* | Asia |
| 208 | SAMEA3497805 | Meishan | ENA | 10.28 | *Sus scrofa* | Asia |
| 209 | SAMEA3497807 | Meishan | ENA | 10.83 | *Sus scrofa* | Asia |
| 210 | SAMEA3497808 | Meishan | ENA | 10.70 | *Sus scrofa* | Asia |
| 211 | SAMEA3497809 | Meishan | ENA | 16.79 | *Sus scrofa* | Asia |
| 212 | SAMEA3497810 | Wannan Spotted | ENA | 10.68 | *Sus scrofa* | Asia |
| 213 | SAMEA3497811 | Wannan Spotted | ENA | 10.18 | *Sus scrofa* | Asia |
| 214 | SAMEA3497814 | Zang | ENA | 10.91 | *Sus scrofa* | Asia |
| 215 | SAMEA3497816 | Asian Wild Boar | ENA | 12.78 | *Sus scrofa* | Asia |
| 216 | SAMEA3497819 | Asian Wild Boar | ENA | 14.70 | *Sus scrofa* | Asia |
| 217 | SAMEA3497821 | Asian Wild Boar | ENA | 13.06 | *Sus scrofa* | Asia |
| 218 | SAMEA3497822 | Asian Wild Boar | ENA | 14.17 | *Sus scrofa* | Asia |
| 219 | SAMEA3497823 | Asian Wild Boar | ENA | 14.31 | *Sus scrofa* | Asia |
| 220 | SAMEA3497824 | Angler Sattleschwein | ENA | 12.79 | *Sus scrofa* | Europe |
| 221 | SAMEA3497826 | Bunte Bentheimer | ENA | 15.39 | *Sus scrofa* | Europe |
| 222 | SAMEA3497827 | Berkshire | ENA | 12.93 | *Sus scrofa* | Europe |
| 223 | SAMEA3497828 | Berkshire | ENA | 10.68 | *Sus scrofa* | Europe |
| 224 | SAMEA3497830 | British Saddleback | ENA | 12.30 | *Sus scrofa* | Europe |
| 225 | SAMEA3497832 | Chato Murciano | ENA | 11.10 | *Sus scrofa* | Europe |
| 226 | SAMEA3497835 | Casertana | ENA | 12.44 | *Sus scrofa* | Europe |
| 227 | SAMEA3497836 | Casertana | ENA | 10.77 | *Sus scrofa* | Europe |
| 228 | SAMEA3497837 | Duroc | ENA | 13.90 | *Sus scrofa* | Europe |
| 229 | SAMEA3497838 | Duroc | ENA | 13.39 | *Sus scrofa* | Europe |
| 230 | SAMEA3497843 | Hampshire | ENA | 10.20 | *Sus scrofa* | Europe |
| 231 | SAMEA3497844 | Hampshire | ENA | 10.61 | *Sus scrofa* | Europe |
| 232 | SAMEA3497851 | Landrace | ENA | 11.39 | *Sus scrofa* | Europe |
| 233 | SAMEA3497852 | Landrace | ENA | 12.57 | *Sus scrofa* | Europe |
| 234 | SAMEA3497853 | Landrace | ENA | 12.35 | *Sus scrofa* | Europe |
| 235 | SAMEA3497854 | Mangalica | ENA | 10.43 | *Sus scrofa* | Europe |
| 236 | SAMEA3497855 | Mangalica | ENA | 11.82 | *Sus scrofa* | Europe |
| 237 | SAMEA3497856 | Middle White | ENA | 12.75 | *Sus scrofa* | Europe |
| 238 | SAMEA3497862 | Tamworth | ENA | 12.33 | *Sus scrofa* | Europe |
| 239 | SAMEA3497863 | Tamworth | ENA | 13.86 | *Sus scrofa* | Europe |
| 240 | SAMEA3497866 | European Wild Boar | ENA | 10.93 | *Sus scrofa* | Europe |
| 241 | SAMEA3497867 | European Wild Boar | ENA | 13.09 | *Sus scrofa* | Europe |
| 242 | SAMEA3497868 | European Wild Boar | ENA | 11.68 | *Sus scrofa* | Europe |
| 243 | SAMEA3497876 | European Wild Boar | ENA | 11.41 | *Sus scrofa* | Europe |
| 244 | SAMEA3497879 | European Wild Boar | ENA | 12.62 | *Sus scrofa* | Europe |
| 245 | SAMEA3497885 | Near East Wild Boar | ENA | 12.23 | *Sus scrofa* | Asia |
| 246 | SAMEA3497886 | European Wild Boar | ENA | 14.58 | *Sus scrofa* | Europe |
| 247 | SAMEA3497887 | European Wild Boar | ENA | 14.03 | *Sus scrofa* | Europe |
| 248 | SAMEA3497888 | European Wild Boar | ENA | 12.69 | *Sus scrofa* | Europe |
| 249 | SAMN02665307 | Duroc | ENA | 12.58 | *Sus scrofa* | Europe |
| 250 | SAMN02665306 | Mangalica | ENA | 15.45 | *Sus scrofa* | Europe |
| 251 | SAMN02665304 | Mangalica | ENA | 21.06 | *Sus scrofa* | Europe |
| 252 | SAMN02665305 | Mangalica | ENA | 15.88 | *Sus scrofa* | Europe |
| 253 | SAMEA4828315 | Goettingen Minipig | ENA | 13.87 | *Sus scrofa* | Mixed |
| 254 | SAMEA4828316 | Goettingen Minipig | ENA | 14.19 | *Sus scrofa* | Mixed |
| 255 | SAMEA4828317 | Goettingen Minipig | ENA | 14.00 | *Sus scrofa* | Mixed |
| 256 | SAMEA4828318 | Goettingen Minipig | ENA | 14.09 | *Sus scrofa* | Mixed |
| 257 | SAMEA4828319 | Goettingen Minipig | ENA | 14.31 | *Sus scrofa* | Mixed |
| 258 | SAMEA4828320 | Goettingen Minipig | ENA | 13.75 | *Sus scrofa* | Mixed |
| 259 | SAMEA4828323 | Goettingen Minipig | ENA | 14.38 | *Sus scrofa* | Mixed |
| 260 | SAMEA4828324 | Goettingen Minipig | ENA | 12.71 | *Sus scrofa* | Mixed |
| 261 | SAMEA4828325 | MiniLEWE | ENA | 14.58 | *Sus scrofa* | Mixed |
| 262 | SAMEA4828326 | MiniLEWE | ENA | 15.25 | *Sus scrofa* | Mixed |
| 263 | SAMN06895011 | Large White | ENA | 13.46 | *Sus scrofa* | Europe |
| 264 | SAMN06895012 | Iberian | ENA | 13.49 | *Sus scrofa* | Europe |
| 265 | SAMN03031126 | Duroc | ENA | 14.44 | *Sus scrofa* | Europe |
| 266 | SAMN03031127 | Duroc | ENA | 16.23 | *Sus scrofa* | Europe |
| 267 | SAMN03031128 | Duroc | ENA | 15.51 | *Sus scrofa* | Europe |
| 268 | SAMN03031132 | Duroc | ENA | 14.39 | *Sus scrofa* | Europe |
| 269 | SAMN03031133 | Duroc | ENA | 15.33 | *Sus scrofa* | Europe |
| 270 | SAMN03031134 | Duroc | ENA | 14.00 | *Sus scrofa* | Europe |
| 271 | SAMN03031136 | Duroc | ENA | 15.56 | *Sus scrofa* | Europe |
| 272 | SAMN03031138 | Duroc | ENA | 13.70 | *Sus scrofa* | Europe |
| 273 | SAMN03031140 | Duroc | ENA | 14.54 | *Sus scrofa* | Europe |
| 274 | SAMN03031142 | Duroc | ENA | 13.94 | *Sus scrofa* | Europe |
| 275 | SAMN03031143 | Duroc | ENA | 13.45 | *Sus scrofa* | Europe |
| 276 | SAMN03031144 | Duroc | ENA | 12.69 | *Sus scrofa* | Europe |
| 277 | SAMN03031147 | Landrace | ENA | 13.14 | *Sus scrofa* | Europe |
| 278 | SAMN03031148 | Landrace | ENA | 12.51 | *Sus scrofa* | Europe |
| 279 | SAMN03031149 | Landrace | ENA | 12.18 | *Sus scrofa* | Europe |
| 280 | SAMN03031150 | Landrace | ENA | 10.04 | *Sus scrofa* | Europe |
| 281 | SAMN03031152 | Landrace | ENA | 10.57 | *Sus scrofa* | Europe |
| 282 | SAMN03031153 | Landrace | ENA | 10.68 | *Sus scrofa* | Europe |
| 283 | SAMN03031154 | Landrace | ENA | 11.76 | *Sus scrofa* | Europe |
| 284 | SAMN03031155 | Landrace | ENA | 11.01 | *Sus scrofa* | Europe |
| 285 | SAMN03031156 | Landrace | ENA | 11.12 | *Sus scrofa* | Europe |
| 286 | SAMN03031157 | Landrace | ENA | 12.06 | *Sus scrofa* | Europe |
| 287 | SAMN03031158 | Landrace | ENA | 12.42 | *Sus scrofa* | Europe |
| 288 | SAMN03031159 | Yucatan miniature pig | ENA | 13.73 | *Sus scrofa* | Europe |
| 289 | SAMN03031160 | Yucatan miniature pig | ENA | 14.54 | *Sus scrofa* | Europe |
| 290 | SAMN03031161 | Yucatan miniature pig | ENA | 12.74 | *Sus scrofa* | Europe |
| 291 | SAMN03031162 | Yucatan miniature pig | ENA | 13.73 | *Sus scrofa* | Europe |
| 292 | SAMN03031163 | Yucatan miniature pig | ENA | 12.88 | *Sus scrofa* | Europe |
| 293 | SAMN03031164 | Yucatan miniature pig | ENA | 12.45 | *Sus scrofa* | Europe |
| 294 | SAMN03031165 | Yucatan miniature pig | ENA | 12.78 | *Sus scrofa* | Europe |
| 295 | SAMN03031166 | Yucatan miniature pig | ENA | 14.20 | *Sus scrofa* | Europe |
| 296 | SAMN03031167 | Yucatan miniature pig | ENA | 10.39 | *Sus scrofa* | Europe |
| 297 | SAMN03031169 | Yucatan miniature pig | ENA | 12.27 | *Sus scrofa* | Europe |
| 298 | SAMN03031170 | Yucatan miniature pig | ENA | 14.02 | *Sus scrofa* | Europe |
| 299 | SAMN03031171 | Asian Wild Boar | ENA | 14.10 | *Sus scrofa* | Asia |
| 300 | SAMN03031172 | Asian Wild Boar | ENA | 10.10 | *Sus scrofa* | Asia |
| 301 | SAMN03031174 | Asian Wild Boar | ENA | 14.52 | *Sus scrofa* | Asia |
| 302 | SAMN03031175 | Asian Wild Boar | ENA | 13.69 | *Sus scrofa* | Asia |
| 303 | SAMN03031176 | Asian Wild Boar | ENA | 12.85 | *Sus scrofa* | Asia |
| 304 | SAMN03031177 | Asian Wild Boar | ENA | 10.76 | *Sus scrofa* | Asia |
| 305 | SAMN03031178 | Asian Wild Boar | ENA | 13.48 | *Sus scrofa* | Asia |
| 306 | SAMN03031179 | Asian Wild Boar | ENA | 13.58 | *Sus scrofa* | Asia |
| 307 | SAMN03031180 | Asian Wild Boar | ENA | 11.98 | *Sus scrofa* | Asia |
| 308 | SAMN03031182 | Yorkshire | ENA | 12.17 | *Sus scrofa* | Europe |
| 309 | SAMN03031183 | Yorkshire | ENA | 11.68 | *Sus scrofa* | Europe |
| 310 | SAMN03031184 | Yorkshire | ENA | 10.33 | *Sus scrofa* | Europe |
| 311 | SAMN03031185 | Yorkshire | ENA | 10.32 | *Sus scrofa* | Europe |
| 312 | SAMN03031186 | Yorkshire | ENA | 12.07 | *Sus scrofa* | Europe |
| 313 | SAMN03031187 | Yorkshire | ENA | 12.26 | *Sus scrofa* | Europe |
| 314 | SAMN03031191 | Yorkshire | ENA | 11.98 | *Sus scrofa* | Europe |
| 315 | SAMN03031193 | Yorkshire | ENA | 13.17 | *Sus scrofa* | Europe |
| 316 | SAMN03031192 | Yorkshire | ENA | 12.83 | *Sus scrofa* | Europe |
| 317 | SAMN03031194 | Yorkshire | ENA | 12.77 | *Sus scrofa* | Europe |
| 318 | SAMN06115544 | Rongchang | ENA | 20.91 | *Sus scrofa* | Asia |
| 319 | SAMN06115548 | Duroc | ENA | 12.65 | *Sus scrofa* | Europe |
| 320 | SAMN06115553 | Rongchang | ENA | 10.80 | *Sus scrofa* | Asia |
| 321 | SAMN06115552 | Rongchang | ENA | 13.18 | *Sus scrofa* | Asia |
| 322 | SAMN06289796 | Iberian | ENA | 12.28 | *Sus scrofa* | Europe |
| 323 | SAMN06289795 | Iberian | ENA | 11.93 | *Sus scrofa* | Europe |
| 324 | SAMN08689107 | Wuzhishan minipig | ENA | 13.61 | *Sus scrofa* | Asia |
| 325 | SAMN03396579 | Isla del Coco feral pig | ENA | 11.96 | *Sus scrofa* | Mixed |
| 326 | SAMN03396581 | Tamworth | ENA | 14.35 | *Sus scrofa* | Europe |
| 327 | SAMN03396582 | Yucatan miniature pig | ENA | 15.54 | *Sus scrofa* | Europe |
| 328 | SAMN03421607 | Iberian | ENA | 16.05 | *Sus scrofa* | Europe |
| 329 | SAMN05362552 | European Wild Boar | ENA | 13.84 | *Sus scrofa* | Europe |
| 330 | SAMN05362553 | European Wild Boar | ENA | 12.23 | *Sus scrofa* | Europe |
| 331 | SAMN05362554 | Iberian | ENA | 13.68 | *Sus scrofa* | Europe |
| 332 | SAMEA3497818 | Asian Wild Boar | ENA | 20.15 | *Sus scrofa* | Asia |
| 333 | SAMEA5059488 | Duroc | ENA | 14.01 | *Sus scrofa* | Europe |
| 334 | SAMEA5059489 | Duroc | ENA | 19.54 | *Sus scrofa* | Europe |
| 335 | SAMEA5730044 | Kune Kune | ENA | 20.81 | *Sus scrofa* | Mixed |
| 336 | SAMEA5730045 | Casertana | ENA | 14.85 | *Sus scrofa* | Europe |
| 337 | SAMEA5730046 | Schweizer Edelschwein | ENA | 17.28 | *Sus scrofa* | Europe |
| 338 | SAMEA5730047 | Pietrain | ENA | 17.68 | *Sus scrofa* | Europe |
| 339 | SAMEA5730048 | Kune Kune | ENA | 15.70 | *Sus scrofa* | Mixed |
| 340 | SAMEA5730050 | Pietrain | ENA | 25.25 | *Sus scrofa* | Europe |
| 341 | SAMEA5730051 | Pietrain | ENA | 25.71 | *Sus scrofa* | Europe |
| 342 | SAMEA5730052 | Pietrain | ENA | 17.12 | *Sus scrofa* | Europe |
| 343 | SAMEA5772924 | Pietrain | ENA | 10.38 | *Sus scrofa* | Europe |
| 344 | SAMEA6141238 | *Sus barbatus* | ENA | 11.22 | *Sus barbatus* | / |
| 345 | SAMEA6141239 | *Sus barbatus* | ENA | 20.51 | *Sus barbatus* | / |
| 346 | SAMEA6141240 | *Sus barbatus* | ENA | 11.59 | *Sus barbatus* | / |
| 347 | SAMEA6141241 | *Sus cebifrons* | ENA | 11.88 | *Sus cebifrons* | / |
| 348 | SAMEA6141242 | *Sus cebifrons* | ENA | 21.97 | *Sus cebifrons* | / |
| 349 | SAMEA6141244 | *Sus celebensis* | ENA | 11.95 | *Sus celebensis* | / |
| 350 | SAMEA6141245 | *Sus verrucosus* | ENA | 12.56 | *Sus verrucosus* | / |
| 351 | SAMEA6141246 | *Sus verrucosus* | ENA | 13.66 | *Sus verrucosus* | / |
| 352 | SAMEA6798259 | Yorkshire | ENA | 15.70 | *Sus scrofa* | Europe |
| 353 | SAMEA6798260 | Yorkshire | ENA | 16.14 | *Sus scrofa* | Europe |
| 354 | SAMEA6798261 | Yorkshire | ENA | 13.80 | *Sus scrofa* | Europe |
| 355 | SAMEA6798262 | Yorkshire | ENA | 13.12 | *Sus scrofa* | Europe |
| 356 | SAMEA6798263 | Yorkshire | ENA | 16.08 | *Sus scrofa* | Europe |
| 357 | SAMEA6813549 | Large White | ENA | 11.19 | *Sus scrofa* | Europe |
| 358 | SAMEA6813550 | Large White | ENA | 10.62 | *Sus scrofa* | Europe |
| 359 | SAMEA6813551 | Large White | ENA | 13.73 | *Sus scrofa* | Europe |
| 360 | SAMEA6813552 | Large White | ENA | 14.55 | *Sus scrofa* | Europe |
| 361 | SAMEA6813553 | Large White | ENA | 13.89 | *Sus scrofa* | Europe |
| 362 | SAMEA7059264 | Large White | ENA | 11.37 | *Sus scrofa* | Europe |
| 363 | SAMEA7059265 | Large White | ENA | 15.64 | *Sus scrofa* | Europe |
| 364 | SAMEA7059266 | Large White | ENA | 12.86 | *Sus scrofa* | Europe |
| 365 | SAMEA7059267 | Large White | ENA | 14.05 | *Sus scrofa* | Europe |
| 366 | SAMEA7059268 | Large White | ENA | 10.57 | *Sus scrofa* | Europe |
| 367 | SAMEA7059270 | Large White | ENA | 10.85 | *Sus scrofa* | Europe |
| 368 | SAMEA7059271 | Large White | ENA | 13.48 | *Sus scrofa* | Europe |
| 369 | SAMEA7059272 | Large White | ENA | 19.12 | *Sus scrofa* | Europe |
| 370 | SAMEA7059273 | Large White | ENA | 10.33 | *Sus scrofa* | Europe |
| 371 | SAMEA7059276 | Large White | ENA | 15.91 | *Sus scrofa* | Europe |
| 372 | SAMEA7059277 | Large White | ENA | 12.33 | *Sus scrofa* | Europe |
| 373 | SAMEA7059278 | Large White | ENA | 26.14 | *Sus scrofa* | Europe |
| 374 | SAMEA7059283 | Large White | ENA | 11.67 | *Sus scrofa* | Europe |
| 375 | SAMEA7059285 | Large White | ENA | 40.34 | *Sus scrofa* | Europe |
| 376 | SAMEA7059286 | Large White | ENA | 10.31 | *Sus scrofa* | Europe |
| 377 | SAMEA7059287 | Large White | ENA | 12.91 | *Sus scrofa* | Europe |
| 378 | SAMEA7059291 | Large White | ENA | 13.81 | *Sus scrofa* | Europe |
| 379 | SAMEA7059292 | Large White | ENA | 16.20 | *Sus scrofa* | Europe |
| 380 | SAMEA7059293 | Large White | ENA | 13.13 | *Sus scrofa* | Europe |
| 381 | SAMEA7059297 | Large White | ENA | 16.58 | *Sus scrofa* | Europe |
| 382 | SAMEA7059298 | Large White | ENA | 15.60 | *Sus scrofa* | Europe |
| 383 | SAMEA7059299 | Large White | ENA | 10.85 | *Sus scrofa* | Europe |
| 384 | SAMEA7059305 | Large White | ENA | 14.36 | *Sus scrofa* | Europe |
| 385 | SAMEA7059307 | Large White | ENA | 16.54 | *Sus scrofa* | Europe |
| 386 | SAMEA7059308 | Large White | ENA | 17.29 | *Sus scrofa* | Europe |
| 387 | SAMEA7059309 | Large White | ENA | 14.74 | *Sus scrofa* | Europe |
| 388 | SAMEA7059311 | Large White | ENA | 17.27 | *Sus scrofa* | Europe |
| 389 | SAMEA7059312 | Large White | ENA | 15.95 | *Sus scrofa* | Europe |
| 390 | SAMEA7059313 | Large White | ENA | 15.24 | *Sus scrofa* | Europe |
| 391 | SAMEA7059314 | Large White | ENA | 16.03 | *Sus scrofa* | Europe |
| 392 | SAMEA7059315 | Large White | ENA | 12.96 | *Sus scrofa* | Europe |
| 393 | SAMEA7059317 | Large White | ENA | 14.00 | *Sus scrofa* | Europe |
| 394 | SAMEA7059318 | Large White | ENA | 20.34 | *Sus scrofa* | Europe |
| 395 | SAMEA7059319 | Large White | ENA | 12.78 | *Sus scrofa* | Europe |
| 396 | SAMEA7059320 | Large White | ENA | 13.33 | *Sus scrofa* | Europe |
| 397 | SAMEA7059321 | Large White | ENA | 20.45 | *Sus scrofa* | Europe |
| 398 | SAMEA7059323 | Large White | ENA | 18.46 | *Sus scrofa* | Europe |
| 399 | SAMEA7059324 | Large White | ENA | 15.74 | *Sus scrofa* | Europe |
| 400 | SAMEA7059325 | Large White | ENA | 17.76 | *Sus scrofa* | Europe |
| 401 | SAMEA7059327 | Large White | ENA | 19.11 | *Sus scrofa* | Europe |
| 402 | SAMEA7059328 | Large White | ENA | 20.63 | *Sus scrofa* | Europe |
| 403 | SAMEA7059329 | Large White | ENA | 14.69 | *Sus scrofa* | Europe |
| 404 | SAMEA7059330 | Large White | ENA | 14.97 | *Sus scrofa* | Europe |
| 405 | SAMEA7059331 | Large White | ENA | 13.18 | *Sus scrofa* | Europe |
| 406 | SAMEA7059332 | Large White | ENA | 15.68 | *Sus scrofa* | Europe |
| 407 | SAMEA7059333 | Large White | ENA | 13.08 | *Sus scrofa* | Europe |
| 408 | SAMEA7059334 | Large White | ENA | 10.68 | *Sus scrofa* | Europe |
| 409 | SAMEA7059336 | Large White | ENA | 16.72 | *Sus scrofa* | Europe |
| 410 | SAMEA7059337 | Large White | ENA | 16.59 | *Sus scrofa* | Europe |
| 411 | SAMEA7059338 | Large White | ENA | 12.50 | *Sus scrofa* | Europe |
| 412 | SAMEA7059339 | Large White | ENA | 16.80 | *Sus scrofa* | Europe |
| 413 | SAMN02904855 | European Wild Boar | ENA | 14.49 | *Sus scrofa* | Europe |
| 414 | SAMN02904857 | Iberian | ENA | 11.95 | *Sus scrofa* | Europe |
| 415 | SAMN03160621 | Korean pig | ENA | 12.99 | *Sus scrofa* | Asia |
| 416 | SAMN03160622 | Korean pig | ENA | 15.25 | *Sus scrofa* | Asia |
| 417 | SAMN03160623 | Korean pig | ENA | 14.89 | *Sus scrofa* | Asia |
| 418 | SAMN03160624 | Korean pig | ENA | 10.80 | *Sus scrofa* | Asia |
| 419 | SAMN03160625 | Korean pig | ENA | 14.38 | *Sus scrofa* | Asia |
| 420 | SAMN03160626 | Korean pig | ENA | 13.73 | *Sus scrofa* | Asia |
| 421 | SAMN03160627 | Jeju black pig | ENA | 12.93 | *Sus scrofa* | Asia |
| 422 | SAMN03160628 | Jeju black pig | ENA | 15.34 | *Sus scrofa* | Asia |
| 423 | SAMN03160629 | Jeju black pig | ENA | 10.28 | *Sus scrofa* | Asia |
| 424 | SAMN03160630 | Jeju black pig | ENA | 15.41 | *Sus scrofa* | Asia |
| 425 | SAMN03160631 | Jeju black pig | ENA | 13.50 | *Sus scrofa* | Asia |
| 426 | SAMN03160633 | Jeju black pig | ENA | 14.99 | *Sus scrofa* | Asia |
| 427 | SAMN03160634 | Jeju black pig | ENA | 16.42 | *Sus scrofa* | Asia |
| 428 | SAMN04440474 | Hampshire | ENA | 19.97 | *Sus scrofa* | Europe |
| 429 | SAMN04440475 | Berkshire | ENA | 21.26 | *Sus scrofa* | Europe |
| 430 | SAMN04440476 | Landrace | ENA | 16.16 | *Sus scrofa* | Europe |
| 431 | SAMN04440477 | Pietrain | ENA | 13.41 | *Sus scrofa* | Europe |
| 432 | SAMN04440478 | Large White | ENA | 19.71 | *Sus scrofa* | Europe |
| 433 | SAMN06560025 | Rongchang | ENA | 18.43 | *Sus scrofa* | Asia |
| 434 | SAMN06560051 | Rongchang | ENA | 23.27 | *Sus scrofa* | Asia |
| 435 | SAMN06560373 | Diannanxiaoer | ENA | 11.92 | *Sus scrofa* | Asia |
| 436 | SAMN06560458 | Diannanxiaoer | ENA | 12.39 | *Sus scrofa* | Asia |
| 437 | SAMN06562573 | Duroc | ENA | 13.15 | *Sus scrofa* | Europe |
| 438 | SAMN06562584 | Meishan | ENA | 11.81 | *Sus scrofa* | Asia |
| 439 | SAMN06562589 | Meishan | ENA | 10.16 | *Sus scrofa* | Asia |
| 440 | SAMN06562602 | Meishan | ENA | 12.45 | *Sus scrofa* | Asia |
| 441 | SAMN06562956 | Meishan | ENA | 10.49 | *Sus scrofa* | Asia |
| 442 | SAMN06562957 | Dawezi | ENA | 12.21 | *Sus scrofa* | Asia |
| 443 | SAMN06562961 | Duroc | ENA | 12.17 | *Sus scrofa* | Europe |
| 444 | SAMN06562968 | Landrace | ENA | 11.49 | *Sus scrofa* | Europe |
| 445 | SAMN06562971 | Meishan | ENA | 11.66 | *Sus scrofa* | Asia |
| 446 | SAMN06562977 | Meishan | ENA | 10.27 | *Sus scrofa* | Asia |
| 447 | SAMN06562978 | Meishan | ENA | 10.62 | *Sus scrofa* | Asia |
| 448 | SAMN06562981 | Duroc | ENA | 12.29 | *Sus scrofa* | Europe |
| 449 | SAMN06562984 | Meishan | ENA | 11.08 | *Sus scrofa* | Asia |
| 450 | SAMN06562994 | Duroc | ENA | 11.52 | *Sus scrofa* | Europe |
| 451 | SAMN06563284 | Meishan | ENA | 11.90 | *Sus scrofa* | Asia |
| 452 | SAMN06563285 | Yorkshire | ENA | 11.87 | *Sus scrofa* | Europe |
| 453 | SAMN06563306 | Duroc | ENA | 11.30 | *Sus scrofa* | Europe |
| 454 | SAMN06563369 | Meishan | ENA | 10.70 | *Sus scrofa* | Asia |
| 455 | SAMN06563372 | Asian Wild Boar | ENA | 16.10 | *Sus scrofa* | Asia |
| 456 | SAMN06563583 | Meishan | ENA | 12.41 | *Sus scrofa* | Asia |
| 457 | SAMN06564163 | Meishan | ENA | 10.17 | *Sus scrofa* | Asia |
| 458 | SAMN06579320 | Meishan | ENA | 10.66 | *Sus scrofa* | Asia |
| 459 | SAMN06603310 | Meishan | ENA | 10.87 | *Sus scrofa* | Asia |
| 460 | SAMN06603321 | Meishan | ENA | 10.22 | *Sus scrofa* | Asia |
| 461 | SAMN06603369 | Meishan | ENA | 11.36 | *Sus scrofa* | Asia |
| 462 | SAMN06603407 | Meishan | ENA | 10.93 | *Sus scrofa* | Asia |
| 463 | SAMN06603626 | Meishan | ENA | 11.44 | *Sus scrofa* | Asia |
| 464 | SAMN06607518 | Meishan | ENA | 10.53 | *Sus scrofa* | Asia |
| 465 | SAMN06610205 | Meishan | ENA | 10.08 | *Sus scrofa* | Asia |
| 466 | SAMN06610393 | Meishan | ENA | 12.15 | *Sus scrofa* | Asia |
| 467 | SAMN06617789 | Duroc | ENA | 10.68 | *Sus scrofa* | Europe |
| 468 | SAMN09111834 | Duroc | ENA | 10.88 | *Sus scrofa* | Europe |
| 469 | SAMN09878800 | Duroc | ENA | 11.18 | *Sus scrofa* | Europe |
| 470 | SAMN09878801 | Duroc | ENA | 12.85 | *Sus scrofa* | Europe |
| 471 | SAMN09878802 | Duroc | ENA | 11.68 | *Sus scrofa* | Europe |
| 472 | SAMN09930389 | Erhualian | ENA | 24.16 | *Sus scrofa* | Asia |
| 473 | SAMN09930390 | Erhualian | ENA | 24.40 | *Sus scrofa* | Asia |
| 474 | SAMN09930391 | Erhualian | ENA | 23.90 | *Sus scrofa* | Asia |
| 475 | SAMN09930392 | Erhualian | ENA | 23.80 | *Sus scrofa* | Asia |
| 476 | SAMN09930393 | Erhualian | ENA | 24.29 | *Sus scrofa* | Asia |
| 477 | SAMN09930395 | Erhualian | ENA | 23.69 | *Sus scrofa* | Asia |
| 478 | SAMN09930396 | Erhualian | ENA | 22.73 | *Sus scrofa* | Asia |
| 479 | SAMN09930397 | Erhualian | ENA | 24.12 | *Sus scrofa* | Asia |
| 480 | SAMN09930398 | Erhualian | ENA | 23.81 | *Sus scrofa* | Asia |
| 481 | SAMN09930400 | Erhualian | ENA | 23.96 | *Sus scrofa* | Asia |
| 482 | SAMN09930401 | Erhualian | ENA | 24.45 | *Sus scrofa* | Asia |
| 483 | SAMEA7059322 | Large White | ENA | 12.47 | *Sus scrofa* | Europe |
| 484 | SAMN06611257 | Duroc | ENA | 10.50 | *Sus scrofa* | Europe |
| 485 | SAMN06618362 | Duroc | ENA | 11.02 | *Sus scrofa* | Europe |

**Table S3.** **Information on the mitochondrial genomes used in this study.**

| **NCBI Accession ID** | **GI^1^** | **Species** | **Sequence length (bp)** | **Breed/ Species common name** | **Pig breed geographic origins** |
| --- | --- | --- | --- | --- | --- |
| NC_000845.1 | 5835862 | *Sus scrofa* | 16613 | Landrace | European |
| AY337045.1 | 37962869 | *Sus scrofa* | 16585 | Duroc | European |
| AY574046.1 | 45826183 | *Sus scrofa* | 16541 | Hampshire | European |
| KC250275.1 | 443611020 | *Sus scrofa* | 16610 | Large White | European |
| KJ746666.1 | 669176308 | *Sus scrofa* | 16727 | Mangalica | European |
| KP223728.1 | 814936710 | *Sus scrofa* | 16532 | Jeuma | Asian |
| KM433673.1 | 698174237 | *Sus scrofa* | 16699 | Longlin | Asian |
| KM044239.1 | 693583934 | *Sus scrofa* | 16710 | Rongchang | Asian |
| KM094194.1 | 698173772 | *Sus scrofa* | 16741 | Sandu Black | Asian |
| KP681245.1 | 794460880 | *Sus scrofa* | 16689 | Wild Boar | Asian |
| KM259826.1 | 697738682 | *Sus scrofa* | 16709 | Wuyi Black | Asian |
| NC_023541.1 | 589144623 | *Sus cebifrons* | 16473 | Visayan warty pig | / |
| NC_008830.1 | 124358976 | *Phacochoerus africanus* | 16719 | Warthog | / |
| NC_006853.1 | 60101824 | *Bos taurus* | 16338 | Cattle | / |
| NC_005044.2 | 316926505 | *Capra hircus* | 16443 | Goat | / |

^1^ GenInfo Identifier (NCBI).
