# Supplementary material for: A comprehensive atlas of nuclear sequences of mitochondrial origin (NUMT) inserted into the pig genome
Source: Genet Sel Evol. 2024 Sep 16;56:64. doi: 10.1186/s12711-024-00930-6 (PMC11403998; doi:10.1186/s12711-024-00930-6)
Supplement: Supplementary file 3 — Additional file 3: Figure S1. Number of NUMT regions, divided by chromosome, identified in the investigated pig assembled genomes, mapped on the Sscrofa11.1 chromosomes. Acronyms of the assembled genomes are explained in Additional file 1: Table S1. Figure S2. Distribution of NUMT regions identified in one or more assembled genomes and shared in two or more assembled genomes. The X axis reports the number of assembled genomes in which a NUMT region was identified. Numbers range from 0 to 25. Acronyms of the assembled genomes are explained in Additional file 1: Table S1. Figure S3. Graphical representation of the relationships between Sscrofa11.1 chromosome length and number of novel NUMTs found on the chromosome. Chromosome length is shown in in Mb. The regression line shows the trend along with its confidence. Figure S4. Graphical representation of the relationships between WGS dataset sequencing depth and number of novel NUMT found in the dataset. Dots represent the investigated WGS datasets. Figure S5. Heatmap showing the frequencies of the NUMT in different breeds/populations/species as estimated from WGS datasets. The Y axis reports all breeds/populations/species from which WGS datasets were used to estimate the frequency of the carriers of the inserted NUMT into the genome. Information is divided for pig breeds/populations and species or grouped considering all datasets or according to the origin of the pig breeds or investigated species. WGS datasets derived from DNA pools are indicated with an asterisk on the Y axis. Novel NUMT regions identified only in WGS datasets are indicated with an asterisk on the X axis. The frequency is reported with the scale colour, going from dark red to pale pink. Figure 9 shows a clusterisation of the species, breeds and populations based on the same information, considering only groups where at least 10 WGS datasets were analysed. Figure S6. Relationship between the number of assembled genomes sharing a NUMT region and the num [file 12711_2024_930_MOESM3_ESM.docx]

Additional file 3

**A comprehensive atlas of nuclear sequences of mitochondrial origin (NUMT) inserted into the pig genome**

Matteo Bolner, Samuele Bovo, Mohamad Ballan, Giuseppina Schiavo, Valeria Taurisano, Anisa Ribani, Francesca Bertolini and Luca Fontanesi

**Table of content**

**Additional file 3: Figure S1.** Number of NUMT regions per chromosome found in each pig assembled genome, mapped on the reference pig genome chromosomes.

**Additional file 3: Figure S2.** Distribution of NUMT regions identified in one or more assembled genomes and shared in two or more assembled genomes.

**Additional file 3: Figure S3.** Graphical representation of the relationships between WGS dataset sequencing depth (X axis) and number of novel NUMTs found in the dataset (Y axis).

**Additional file 3: Figure S4.** Graphical representation of the relationships between Sscrofa11.1 chromosome size (X axis) and number of novel NUMT found on the chromosome (Y axis).

**Additional file 3: Figure S5.** Heatmap showing the frequencies of the NUMTs in different breeds/populations/species as estimated from WGS datasets.

**Additional file 3: Figure S6.** Relationship between number of assembled genomes sharing a NUMT region (X axis) and number of WGS datasets sharing the NUMT region (Y axis).

***Additional file 3: Figure S8.** Alignments of the NUMT fragments identified in the *Sus cebifrons* and *Phacochoerus africanus* reference genomes with their corresponding linearized and annotated true mitochondrial DNA (mtDNA).

*Figure S7 is reported in Additional file 4.

**Figure S1.** **Number of NUMT regions, divided by chromosome, identified in the investigated pig assembled genomes, mapped on the Sscrofa11.1 chromosomes.** Acronyms of the assembled genomes are explained in Additional file 1 Table S1.


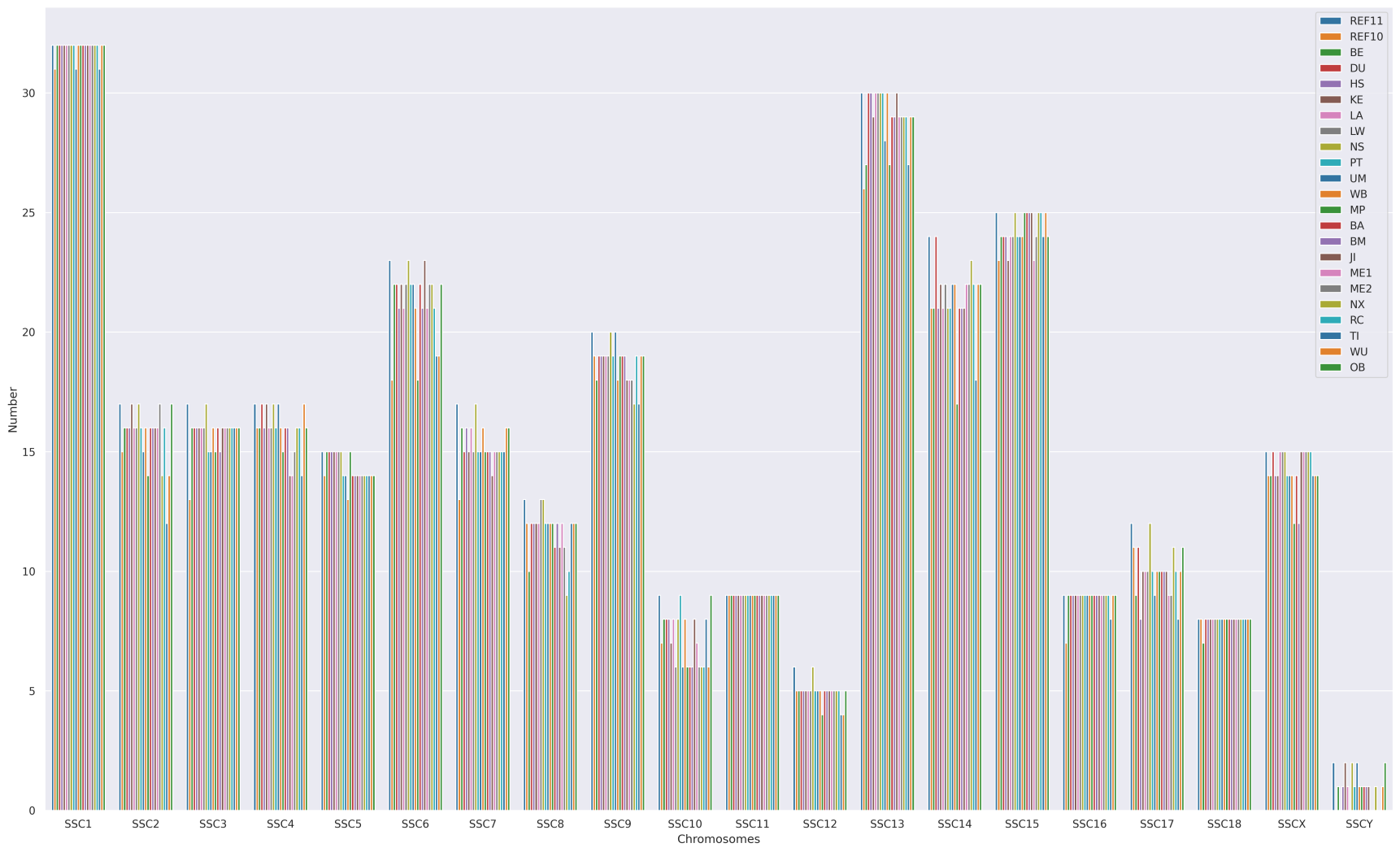


**Figure S2.** **Distribution of NUMT regions identified in one or more assembled genomes and shared in two or more assembled genomes.** The X axis reports the number of assembled genomes in which a NUMT region was identified. Numbers range from 0 (NUMT region identified in only one genome and not shared with any other genomes) to 25 (NUMT region identified in all assembled genomes investigated). Acronyms of the assembled genomes are explained in Additional file 1 Table S1.


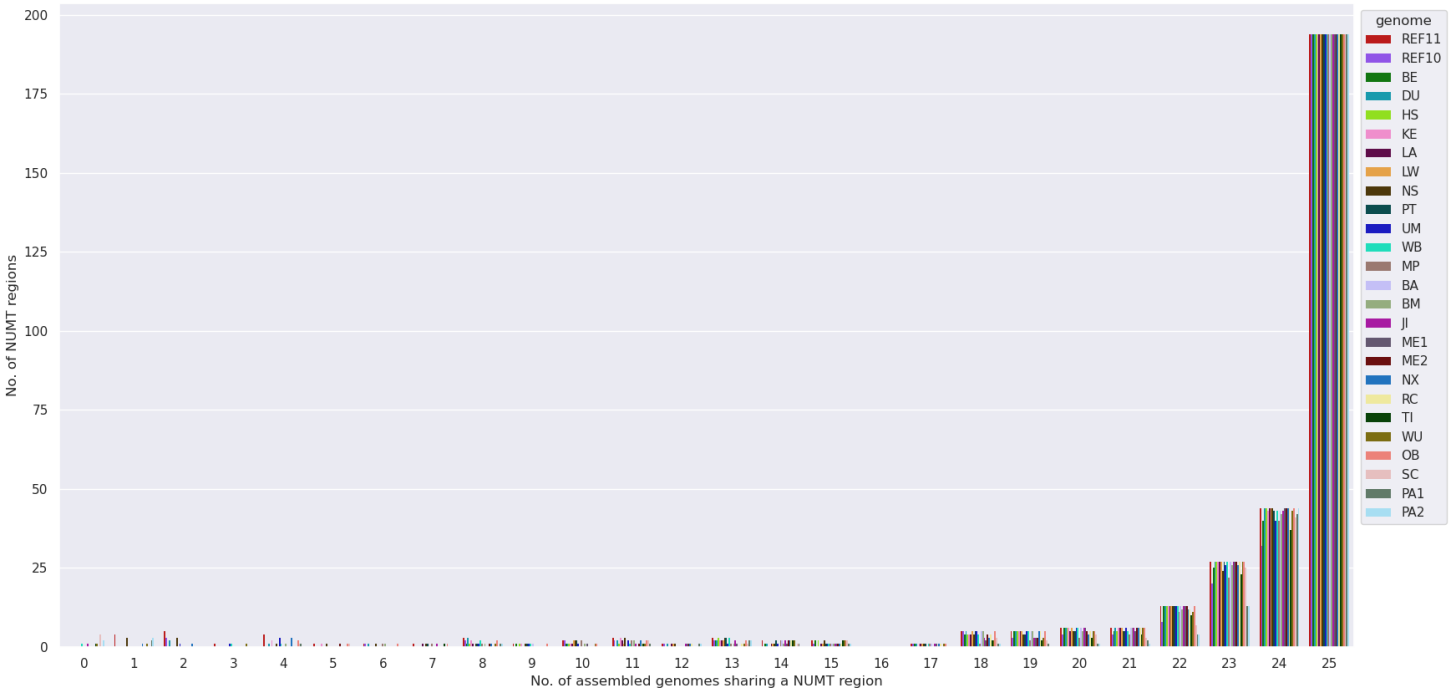


**Figure S3. Graphical representation of the relationships between Sscrofa11.1 chromosome length (X axis) and number of novel NUMT found on the chromosome (Y axis).** Chromosome length is shown in in Mb (Millions of base pairs). The regression line shows the trend along with its confidence (light blue area around the line)


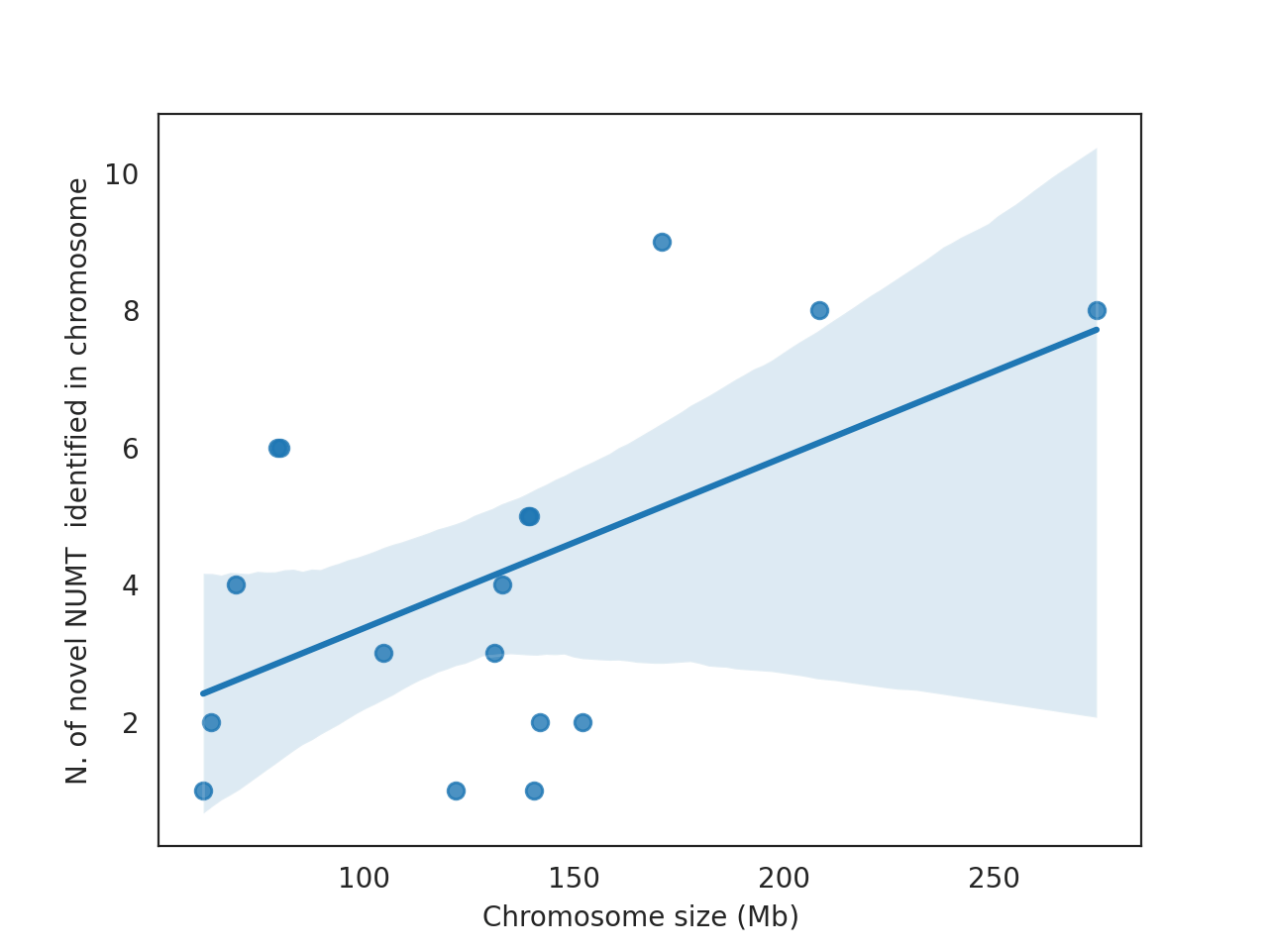


**Figure S4.** **Graphical representation of the relationships between WGS dataset sequencing depth (X axis) and number of novel NUMTs found in the dataset (Y axis).** Dots represent the investigated WGS datasets.


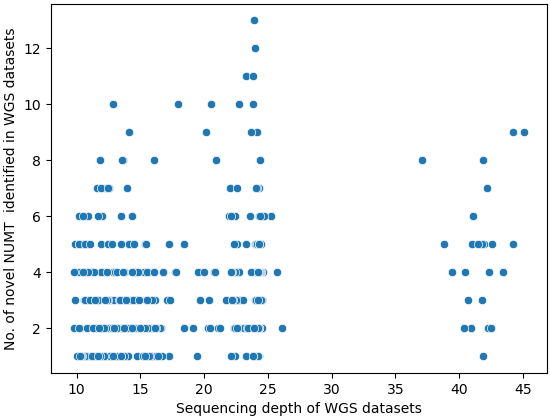


**Figure S5.** **Heatmap showing the frequencies of the NUMTs in different breeds/populations/species as estimated from WGS datasets.** The Y axis reports all breeds/populations/species from which WGS datasets (in parenthesis the number of datasets) were used to estimate the frequency of the carriers of the inserted NUMTs into the genome. Information is divided for pig breeds/populations and species (a) or grouped considering all datasets or according to the origin of the pig breeds or investigated species. WGS datasets derived from DNA pools are indicated with an asterisk (*) of the Y axis. Novel NUMT regions identified only in WGS datasets are indicated with an asterisk (*) on the X axis. The frequency is reported with the scale colour, going from dark red (0%) to pale pink (100%). Figure 7 shows a clusterisation of the species, breeds and populations based on the same information, considering only groups where at least 10 WGS datasets were analysed.


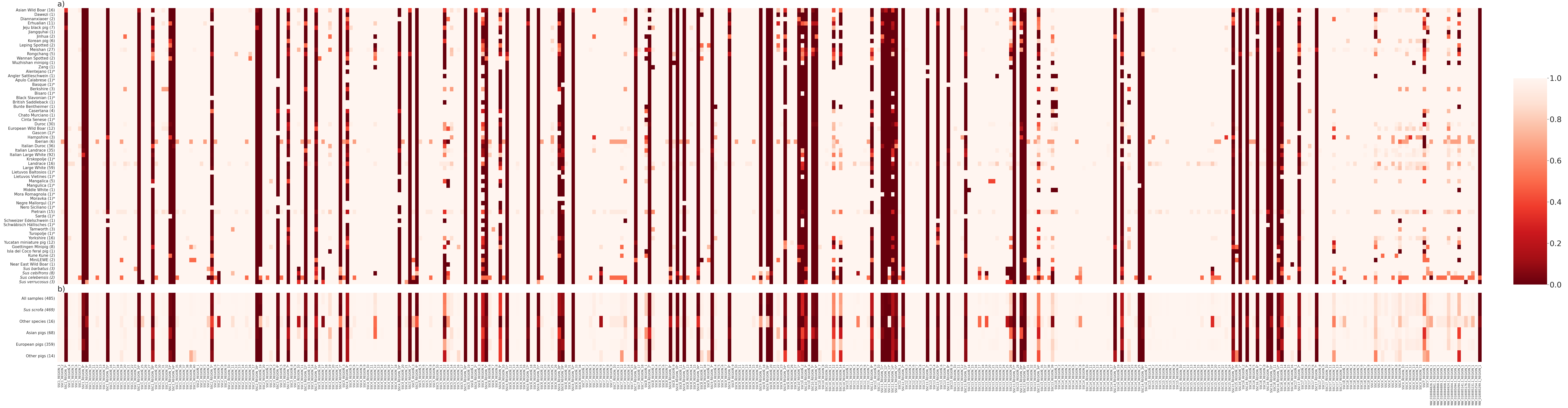


**Figure S6.** **Relationship between the number of assembled genomes sharing a NUMT region (X axis) and the number of WGS datasets sharing the NUMT region (Y axis).**


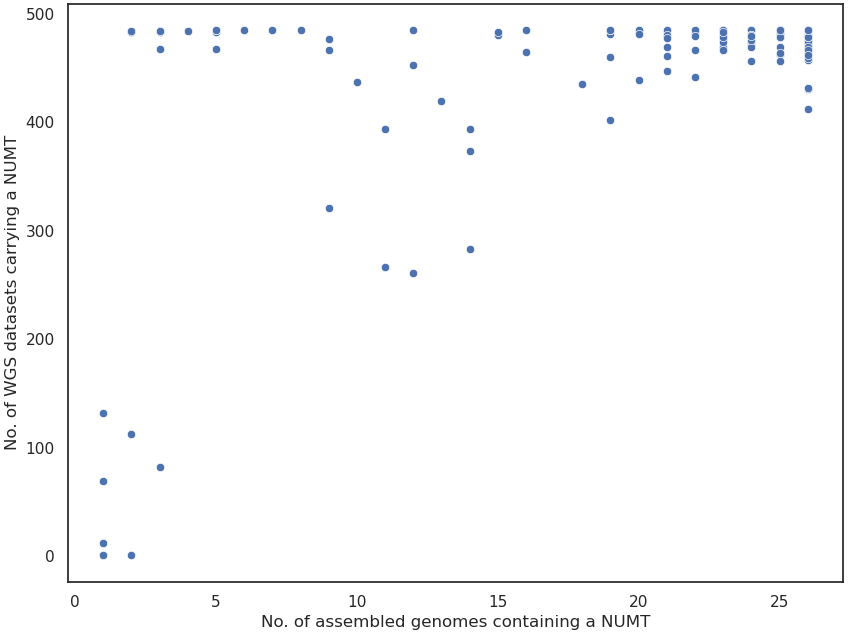


**Figure S8 Alignments of the NUMT fragments identified in the *Sus cebifrons* and *Phacochoerus africanus* reference genomes with their corresponding linearized and annotated true mitochondrial DNA (mtDNA).** In subfigure a) each line represents a NUMT fragment identified in the *S. cebifrons* assembled genome (blue) or in a *S. cebifrons* WGS datasets (yellow); in subfigure b) each line represents a NUMT fragment identified in the *P. africanus* assembled genomes (blue). The mtDNA of both species is annotated with the corresponding genes and D-loop regions.

**
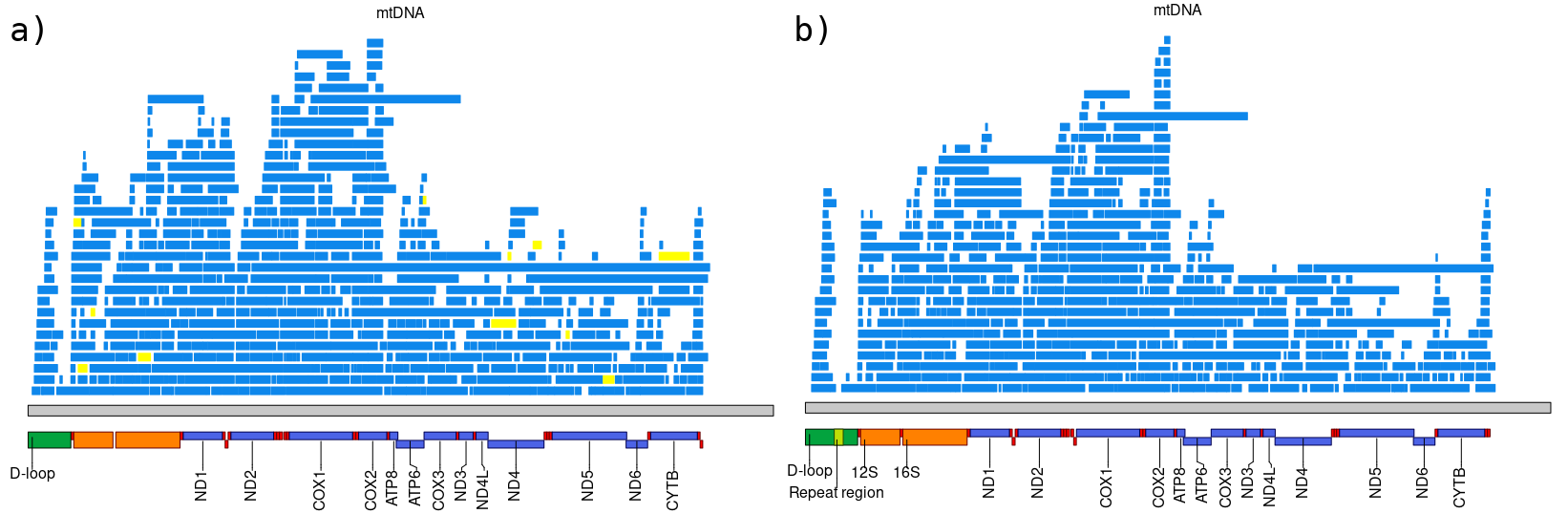
**
