# Supplementary material for: A comprehensive atlas of nuclear sequences of mitochondrial origin (NUMT) inserted into the pig genome
Source: Genet Sel Evol. 2024 Sep 16;56:64. doi: 10.1186/s12711-024-00930-6 (PMC11403998; doi:10.1186/s12711-024-00930-6)
Supplement: Supplementary file 4 — Additional file 4: Figure S7. Phylogenetic trees were obtained for all NUMT regions for which a NUMT sequence was longer than 150 bp. Each tree was computed from the longest NUMT sequence in the region. The title of the tree reports the name of the NUMT region. The X axis of each tree represents the branch length. Polymorphic NUMT regions are colored red, and fixed NUMT regions are colored blue. The NUMT nuclear sequence is compared with the corresponding sequences in the mtDNA of several species. The posterior probability computed by BEAST is reported along each node in the tree, and ranges from 0 to 100. [file 12711_2024_930_MOESM4_ESM.pdf]

Additional file 4

**A comprehensive atlas of nuclear sequences of mitochondrial origin (NUMTs) inserted into the pig genome**

Matteo Bolner, Samuele Bovo, Mohamad Ballan, Giuseppina Schiavo, Valeria Taurisano, Anisa Ribani, Francesca Bertolini and Luca Fontanesi

**Table of content**

**Additional file 4: Figure S7. Phylogenetic trees obtained for all NUMT regions (n. 178) for which a NUMT sequence was longer than 150 bp.**

Each tree was computed from the longest NUMT sequence in the region. The title of the tree reports the name of the NUMT region. The X axis of each tree represents the branch length. Polymorphic NUMT regions are colored red, and fixed NUMT regions are colored blue. The NUMT nuclear sequence is compared with the corresponding sequences in the mitochondrial DNA of several species (the other branches). The posterior probability computed by BEAST is reported along each node in the tree, and ranges from 0 to 100.

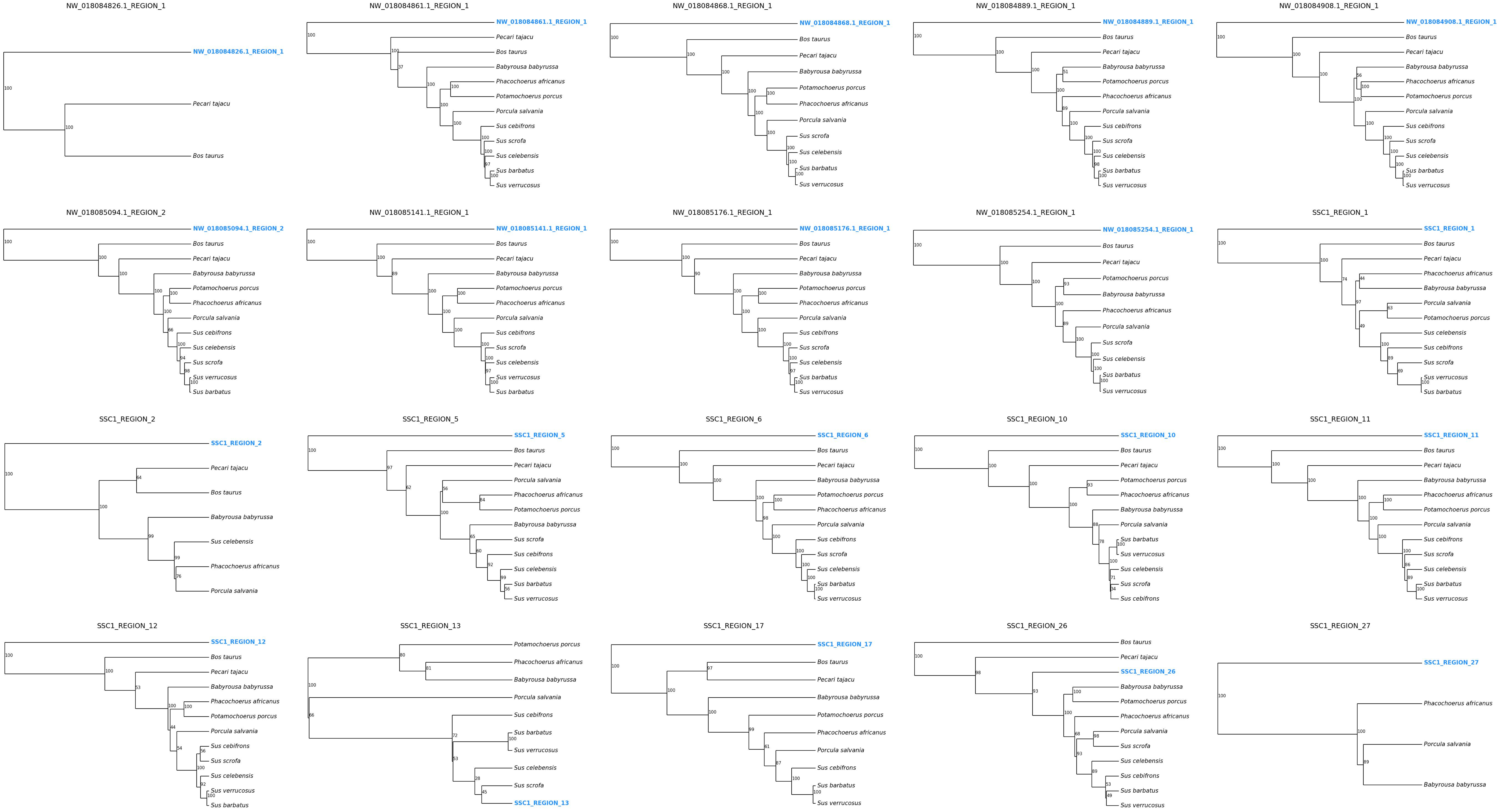
NW_018084826.1_REGION_1
NW_018084826.1_REGION_1
Pecari tajacu
Bos taurus
NW_018084861.1_REGION_1
NW_018084861.1_REGION_1
Pecari tajacu
Bos taurus
Babyrousa babyrussa
Phacochoerus africanus
Potamochoerus porcus
Porcula salvania
Sus cebifrons
Sus scrofa
Sus celebensis
Sus barbatus
Sus verrucosus
NW_018084868.1_REGION_1
NW_018084868.1_REGION_1
Bos taurus
Pecari tajacu
Babyrousa babyrussa
Potamochoerus porcus
Phacochoerus africanus
Porcula salvania
Sus scrofa
Sus celebensis
Sus barbatus
Sus verrucosus
NW_018084889.1_REGION_1
NW_018084889.1_REGION_1
Bos taurus
Pecari tajacu
Babyrousa babyrussa
Potamochoerus porcus
Phacochoerus africanus
Porcula salvania
Sus cebifrons
Sus scrofa
Sus celebensis
Sus barbatus
Sus verrucosus
NW_018084908.1_REGION_1
NW_018084908.1_REGION_1
Bos taurus
Pecari tajacu
Babyrousa babyrussa
Phacochoerus africanus
Potamochoerus porcus
Porcula salvania
Sus cebifrons
Sus scrofa
Sus celebensis
Sus barbatus
Sus verrucosus
NW_018085094.1_REGION_2
NW_018085094.1_REGION_2
Bos taurus
Pecari tajacu
Babyrousa babyrussa
Potamochoerus porcus
Phacochoerus africanus
Porcula salvania
Sus cebifrons
Sus celebensis
Sus scrofa
Sus verrucosus
Sus barbatus
NW_018085141.1_REGION_1
NW_018085141.1_REGION_1
Bos taurus
Pecari tajacu
Babyrousa babyrussa
Potamochoerus porcus
Phacochoerus africanus
Porcula salvania
Sus cebifrons
Sus scrofa
Sus celebensis
Sus verrucosus
Sus barbatus
NW_018085176.1_REGION_1
NW_018085176.1_REGION_1
Bos taurus
Pecari tajacu
Babyrousa babyrussa
Potamochoerus porcus
Phacochoerus africanus
Porcula salvania
Sus cebifrons
Sus scrofa
Sus celebensis
Sus barbatus
Sus verrucosus
NW_018085254.1_REGION_1
NW_018085254.1_REGION_1
Bos taurus
Pecari tajacu
Potamochoerus porcus
Babyrousa babyrussa
Phacochoerus africanus
Porcula salvania
Sus scrofa
Sus celebensis
Sus barbatus
Sus verrucosus
SSC1_REGION_1
SSC1_REGION_1
Bos taurus
Pecari tajacu
Phacochoerus africanus
Babyrousa babyrussa
Porcula salvania
Potamochoerus porcus
Sus celebensis
Sus cebifrons
Sus scrofa
Sus verrucosus
Sus barbatus
SSC1_REGION_2
SSC1_REGION_2
Pecari tajacu
Bos taurus
Babyrousa babyrussa
Sus celebensis
Phacochoerus africanus
Porcula salvania
SSC1_REGION_5
SSC1_REGION_5
Bos taurus
Pecari tajacu
Porcula salvania
Phacochoerus africanus
Potamochoerus porcus
Babyrousa babyrussa
Sus scrofa
Sus cebifrons
Sus celebensis
Sus barbatus
Sus verrucosus
SSC1_REGION_6
SSC1_REGION_6
Bos taurus
Pecari tajacu
Babyrousa babyrussa
Potamochoerus porcus
Phacochoerus africanus
Porcula salvania
Sus cebifrons
Sus scrofa
Sus celebensis
Sus barbatus
Sus verrucosus
SSC1_REGION_10
SSC1_REGION_10
Bos taurus
Pecari tajacu
Potamochoerus porcus
Phacochoerus africanus
Babyrousa babyrussa
Porcula salvania
Sus barbatus
Sus verrucosus
Sus celebensis
Sus scrofa
Sus cebifrons
SSC1_REGION_11
SSC1_REGION_11
Bos taurus
Pecari tajacu
Babyrousa babyrussa
Phacochoerus africanus
Potamochoerus porcus
Porcula salvania
Sus cebifrons
Sus scrofa
Sus celebensis
Sus barbatus
Sus verrucosus
SSC1_REGION_12
SSC1_REGION_12
Bos taurus
Pecari tajacu
Babyrousa babyrussa
Phacochoerus africanus
Potamochoerus porcus
Porcula salvania
Sus cebifrons
Sus scrofa
Sus celebensis
Sus verrucosus
Sus barbatus
SSC1_REGION_13
Potamochoerus porcus
Phacochoerus africanus
Babyrousa babyrussa
Porcula salvania
Sus cebifrons
Sus barbatus
Sus verrucosus
Sus celebensis
Sus scrofa
SSC1_REGION_13
SSC1_REGION_17
SSC1_REGION_17
Bos taurus
Pecari tajacu
Babyrousa babyrussa
Potamochoerus porcus
Phacochoerus africanus
Porcula salvania
Sus cebifrons
Sus barbatus
Sus verrucosus
SSC1_REGION_26
Bos taurus
Pecari tajacu
SSC1_REGION_26
Babyrousa babyrussa
Potamochoerus porcus
Phacochoerus africanus
Porcula salvania
Sus scrofa
Sus celebensis
Sus cebifrons
Sus barbatus
Sus verrucosus
SSC1_REGION_27
SSC1_REGION_27
Phacochoerus africanus
Porcula salvania
Babyrousa babyrussa

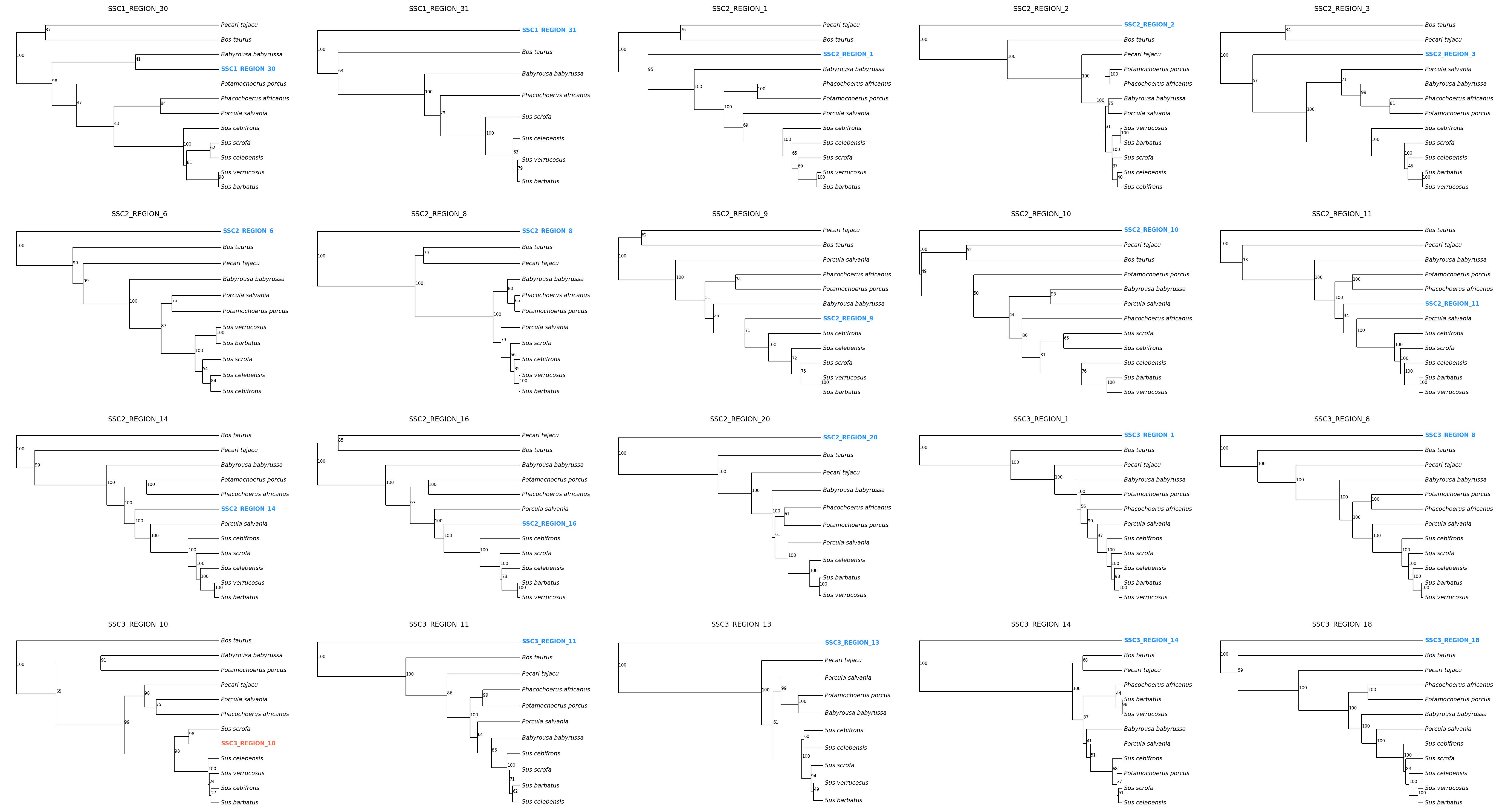
SSC1_REGION_30
Pecari tajacu
Bos taurus
Babyrousa babyrussa
SSC1_REGION_30
Potamochoerus porcus
Phacochoerus africanus
Porcula salvania
Sus cebifrons
Sus scrofa
Sus celebensis
Sus verrucosus
Sus barbatus
SSC1_REGION_31
SSC1_REGION_31
Bos taurus
Babyrousa babyrussa
Phacochoerus africanus
Sus scrofa
Sus celebensis
Sus verrucosus
Sus barbatus
SSC2_REGION_1
Pecari tajacu
Bos taurus
SSC2_REGION_1
Babyrousa babyrussa
Phacochoerus africanus
Potamochoerus porcus
Porcula salvania
Sus cebifrons
Sus celebensis
Sus scrofa
Sus verrucosus
Sus barbatus
SSC2_REGION_2
SSC2_REGION_2
Bos taurus
Pecari tajacu
Potamochoerus porcus
Phacochoerus africanus
Babyrousa babyrussa
Porcula salvania
Sus verrucosus
Sus barbatus
Sus scrofa
Sus celebensis
Sus cebifrons
SSC2_REGION_3
Bos taurus
Pecari tajacu
SSC2_REGION_3
Porcula salvania
Babyrousa babyrussa
Phacochoerus africanus
Potamochoerus porcus
Sus cebifrons
Sus scrofa
Sus celebensis
Sus barbatus
Sus verrucosus
SSC2_REGION_6
SSC2_REGION_6
Bos taurus
Pecari tajacu
Babyrousa babyrussa
Porcula salvania
Potamochoerus porcus
Sus verrucosus
Sus barbatus
Sus scrofa
Sus celebensis
Sus cebifrons
SSC2_REGION_8
SSC2_REGION_8
Bos taurus
Pecari tajacu
Babyrousa babyrussa
Phacochoerus africanus
Potamochoerus porcus
Porcula salvania
Sus scrofa
Sus cebifrons
Sus verrucosus
Sus barbatus
SSC2_REGION_9
Pecari tajacu
Bos taurus
Porcula salvania
Phacochoerus africanus
Potamochoerus porcus
Babyrousa babyrussa
SSC2_REGION_9
Sus cebifrons
Sus celebensis
Sus scrofa
Sus verrucosus
Sus barbatus
SSC2_REGION_10
SSC2_REGION_10
Pecari tajacu
Bos taurus
Potamochoerus porcus
Babyrousa babyrussa
Porcula salvania
Phacochoerus africanus
Sus scrofa
Sus cebifrons
Sus celebensis
Sus barbatus
Sus verrucosus
SSC2_REGION_11
Bos taurus
Pecari tajacu
Babyrousa babyrussa
Potamochoerus porcus
Phacochoerus africanus
SSC2_REGION_11
Porcula salvania
Sus cebifrons
Sus scrofa
Sus celebensis
Sus barbatus
Sus verrucosus
SSC2_REGION_14
Bos taurus
Pecari tajacu
Babyrousa babyrussa
Potamochoerus porcus
Phacochoerus africanus
SSC2_REGION_14
Porcula salvania
Sus cebifrons
Sus scrofa
Sus celebensis
Sus verrucosus
Sus barbatus
SSC2_REGION_16
Pecari tajacu
Bos taurus
Babyrousa babyrussa
Potamochoerus porcus
Phacochoerus africanus
Porcula salvania
SSC2_REGION_16
Sus cebifrons
Sus scrofa
Sus celebensis
Sus barbatus
Sus verrucosus
SSC2_REGION_20
SSC2_REGION_20
Bos taurus
Pecari tajacu
Babyrousa babyrussa
Phacochoerus africanus
Potamochoerus porcus
Porcula salvania
Sus celebensis
Sus barbatus
Sus verrucosus
SSC3_REGION_1
SSC3_REGION_1
Bos taurus
Pecari tajacu
Babyrousa babyrussa
Potamochoerus porcus
Phacochoerus africanus
Porcula salvania
Sus cebifrons
Sus scrofa
Sus celebensis
Sus barbatus
Sus verrucosus
SSC3_REGION_8
SSC3_REGION_8
Bos taurus
Pecari tajacu
Babyrousa babyrussa
Potamochoerus porcus
Phacochoerus africanus
Porcula salvania
Sus cebifrons
Sus scrofa
Sus celebensis
Sus barbatus
Sus verrucosus
SSC3_REGION_10
Bos taurus
Babyrousa babyrussa
Potamochoerus porcus
Pecari tajacu
Porcula salvania
Phacochoerus africanus
Sus scrofa
SSC3_REGION_10
Sus celebensis
Sus verrucosus
Sus cebifrons
Sus barbatus
SSC3_REGION_11
SSC3_REGION_11
Bos taurus
Pecari tajacu
Phacochoerus africanus
Potamochoerus porcus
Porcula salvania
Babyrousa babyrussa
Sus cebifrons
Sus scrofa
Sus barbatus
Sus celebensis
SSC3_REGION_13
SSC3_REGION_13
Pecari tajacu
Porcula salvania
Potamochoerus porcus
Babyrousa babyrussa
Sus cebifrons
Sus celebensis
Sus scrofa
Sus verrucosus
Sus barbatus
SSC3_REGION_14
SSC3_REGION_14
Bos taurus
Pecari tajacu
Phacochoerus africanus
Sus barbatus
Sus verrucosus
Babyrousa babyrussa
Porcula salvania
Sus cebifrons
Potamochoerus porcus
Sus scrofa
Sus celebensis
SSC3_REGION_18
SSC3_REGION_18
Bos taurus
Pecari tajacu
Phacochoerus africanus
Potamochoerus porcus
Babyrousa babyrussa
Porcula salvania
Sus cebifrons
Sus scrofa
Sus celebensis
Sus verrucosus
Sus barbatus

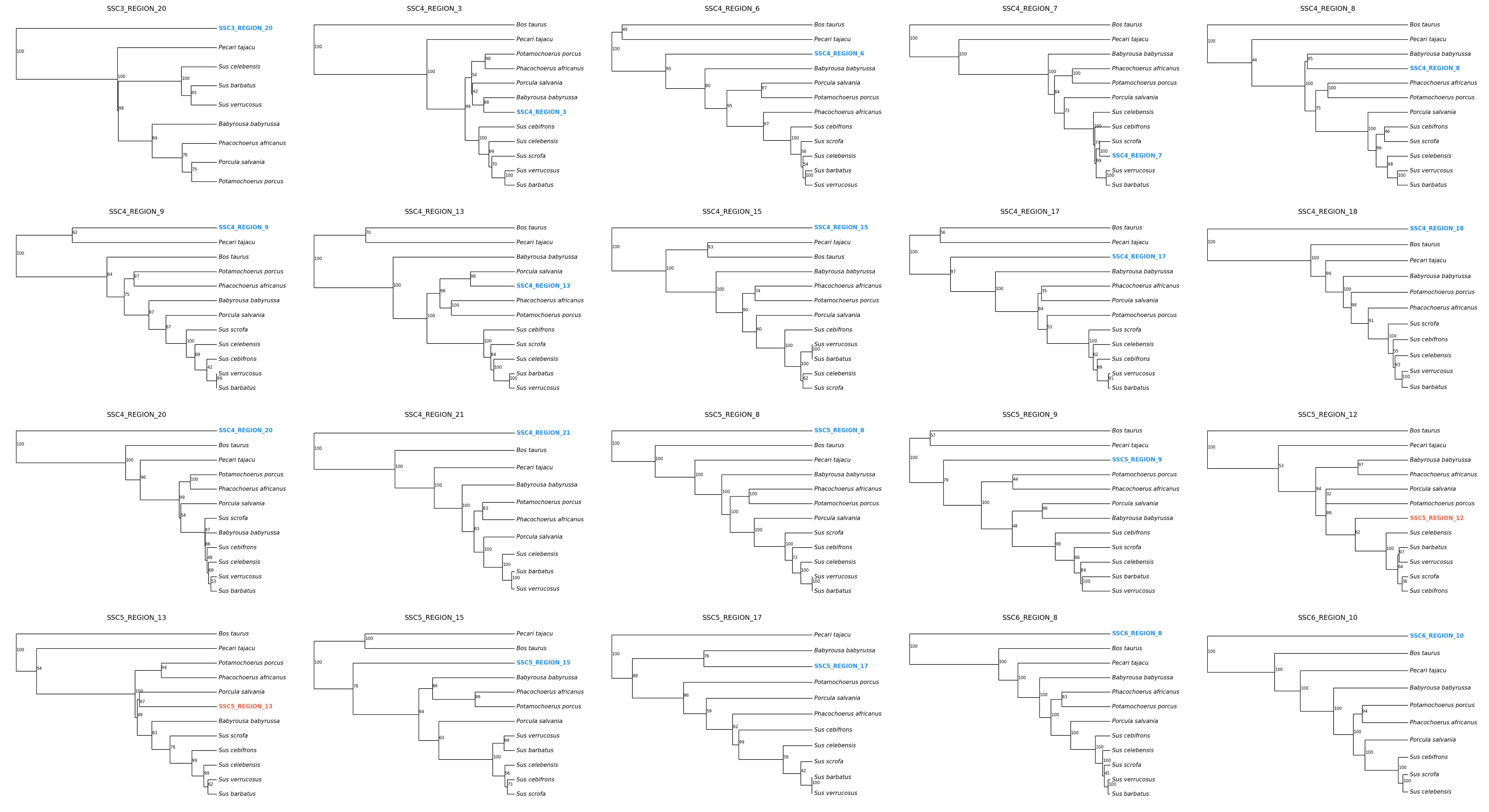
SSC3_REGION_20
SSC3_REGION_20
Pecari tajacu
Sus celebensis
Sus barbatus
Sus verrucosus
Babyrousa babyrussa
Phacochoerus africanus
Porcula salvania
Potamochoerus porcus
SSC4_REGION_3
Bos taurus
Pecari tajacu
Potamochoerus porcus
Phacochoerus africanus
Porcula salvania
Babyrousa babyrussa
SSC4_REGION_3
Sus cebifrons
Sus celebensis
Sus scrofa
Sus verrucosus
Sus barbatus
SSC4_REGION_6
Bos taurus
Pecari tajacu
SSC4_REGION_6
Babyrousa babyrussa
Porcula salvania
Potamochoerus porcus
Phacochoerus africanus
Sus cebifrons
Sus scrofa
Sus celebensis
Sus barbatus
Sus verrucosus
SSC4_REGION_7
Bos taurus
Pecari tajacu
Babyrousa babyrussa
Phacochoerus africanus
Potamochoerus porcus
Porcula salvania
Sus celebensis
Sus cebifrons
Sus scrofa
SSC4_REGION_7
Sus verrucosus
Sus barbatus
SSC4_REGION_8
Bos taurus
Pecari tajacu
Babyrousa babyrussa
SSC4_REGION_8
Phacochoerus africanus
Potamochoerus porcus
Porcula salvania
Sus cebifrons
Sus scrofa
Sus celebensis
Sus verrucosus
Sus barbatus
SSC4_REGION_9
SSC4_REGION_9
Pecari tajacu
Bos taurus
Potamochoerus porcus
Phacochoerus africanus
Babyrousa babyrussa
Porcula salvania
Sus scrofa
Sus celebensis
Sus cebifrons
Sus verrucosus
Sus barbatus
SSC4_REGION_13
Bos taurus
Pecari tajacu
Babyrousa babyrussa
Porcula salvania
SSC4_REGION_13
Phacochoerus africanus
Potamochoerus porcus
Sus cebifrons
Sus scrofa
Sus celebensis
Sus barbatus
Sus verrucosus
SSC4_REGION_15
SSC4_REGION_15
Pecari tajacu
Bos taurus
Babyrousa babyrussa
Phacochoerus africanus
Potamochoerus porcus
Porcula salvania
Sus cebifrons
Sus verrucosus
Sus barbatus
Sus celebensis
Sus scrofa
SSC4_REGION_17
Bos taurus
Pecari tajacu
SSC4_REGION_17
Babyrousa babyrussa
Phacochoerus africanus
Porcula salvania
Potamochoerus porcus
Sus scrofa
Sus celebensis
Sus cebifrons
Sus verrucosus
Sus barbatus
SSC4_REGION_18
SSC4_REGION_18
Bos taurus
Pecari tajacu
Babyrousa babyrussa
Potamochoerus porcus
Phacochoerus africanus
Sus scrofa
Sus cebifrons
Sus celebensis
Sus verrucosus
Sus barbatus
SSC4_REGION_20
SSC4_REGION_20
Bos taurus
Pecari tajacu
Potamochoerus porcus
Phacochoerus africanus
Porcula salvania
Sus scrofa
Babyrousa babyrussa
Sus cebifrons
Sus celebensis
Sus verrucosus
Sus barbatus
SSC4_REGION_21
SSC4_REGION_21
Bos taurus
Pecari tajacu
Babyrousa babyrussa
Potamochoerus porcus
Phacochoerus africanus
Porcula salvania
Sus celebensis
Sus barbatus
Sus verrucosus
SSC5_REGION_8
SSC5_REGION_8
Bos taurus
Pecari tajacu
Babyrousa babyrussa
Phacochoerus africanus
Potamochoerus porcus
Porcula salvania
Sus scrofa
Sus cebifrons
Sus celebensis
Sus verrucosus
Sus barbatus
SSC5_REGION_9
Bos taurus
Pecari tajacu
SSC5_REGION_9
Potamochoerus porcus
Phacochoerus africanus
Porcula salvania
Babyrousa babyrussa
Sus cebifrons
Sus scrofa
Sus celebensis
Sus barbatus
Sus verrucosus
SSC5_REGION_12
Bos taurus
Pecari tajacu
Babyrousa babyrussa
Phacochoerus africanus
Porcula salvania
Potamochoerus porcus
SSC5_REGION_12
Sus celebensis
Sus barbatus
Sus verrucosus
Sus scrofa
Sus cebifrons
SSC5_REGION_13
Bos taurus
Pecari tajacu
Potamochoerus porcus
Phacochoerus africanus
Porcula salvania
SSC5_REGION_13
Babyrousa babyrussa
Sus scrofa
Sus cebifrons
Sus celebensis
Sus verrucosus
Sus barbatus
SSC5_REGION_15
Pecari tajacu
Bos taurus
SSC5_REGION_15
Babyrousa babyrussa
Phacochoerus africanus
Potamochoerus porcus
Porcula salvania
Sus verrucosus
Sus barbatus
Sus celebensis
Sus cebifrons
Sus scrofa
SSC5_REGION_17
Pecari tajacu
Babyrousa babyrussa
SSC5_REGION_17
Potamochoerus porcus
Porcula salvania
Phacochoerus africanus
Sus cebifrons
Sus celebensis
Sus scrofa
Sus barbatus
Sus verrucosus
SSC6_REGION_8
SSC6_REGION_8
Bos taurus
Pecari tajacu
Babyrousa babyrussa
Phacochoerus africanus
Potamochoerus porcus
Porcula salvania
Sus cebifrons
Sus celebensis
Sus scrofa
Sus verrucosus
Sus barbatus
SSC6_REGION_10
SSC6_REGION_10
Bos taurus
Pecari tajacu
Babyrousa babyrussa
Potamochoerus porcus
Phacochoerus africanus
Porcula salvania
Sus cebifrons
Sus scrofa
Sus celebensis

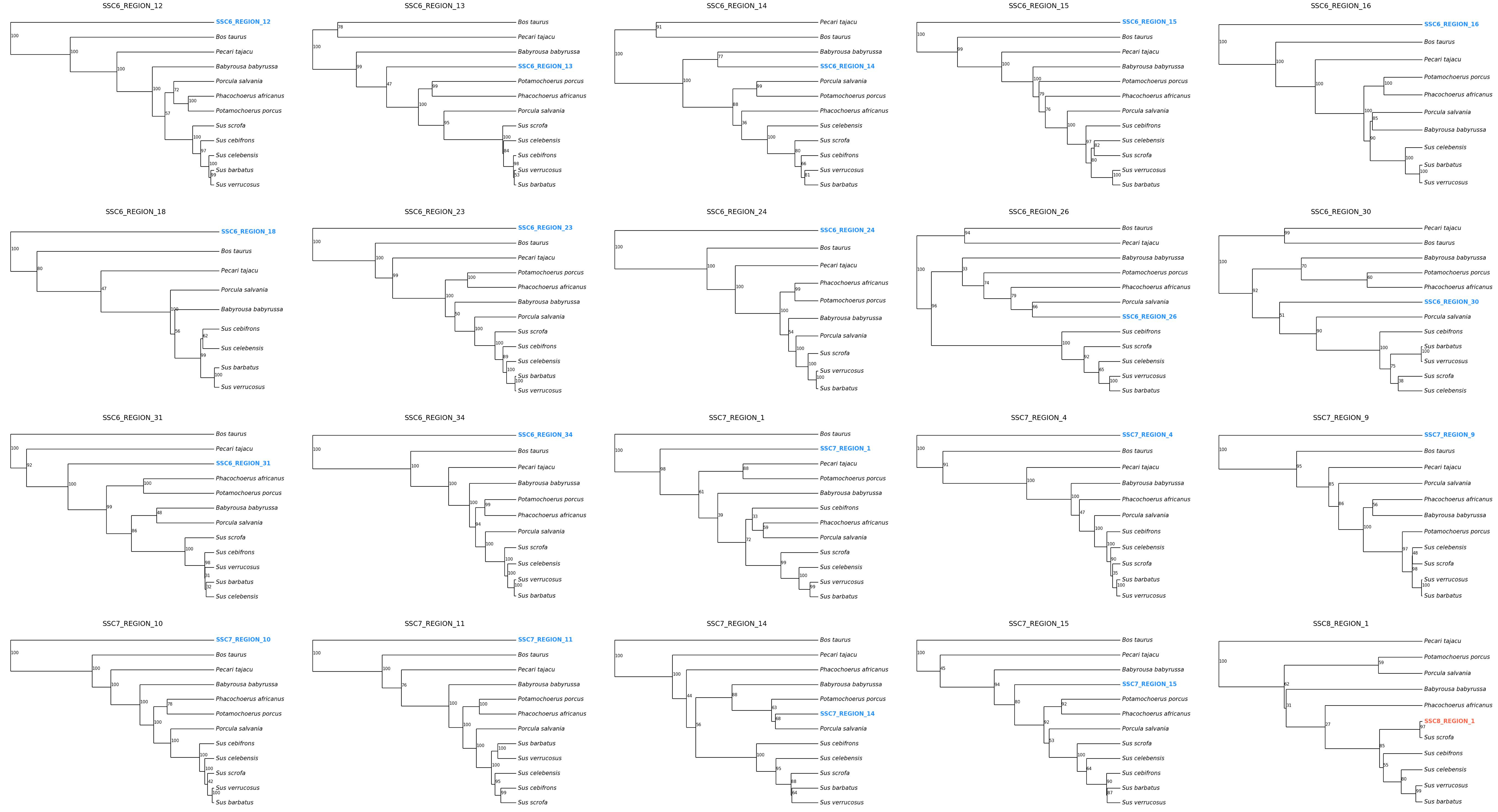
SSC6_REGION_12
SSC6_REGION_12
Bos taurus
Pecari tajacu
Babyrousa babyrussa
Porcula salvania
Phacochoerus africanus
Potamochoerus porcus
Sus scrofa
Sus cebifrons
Sus celebensis
Sus barbatus
Sus verrucosus
SSC6_REGION_13
Bos taurus
Pecari tajacu
Babyrousa babyrussa
SSC6_REGION_13
Potamochoerus porcus
Phacochoerus africanus
Porcula salvania
Sus scrofa
Sus celebensis
Sus cebifrons
Sus verrucosus
Sus barbatus
SSC6_REGION_14
Pecari tajacu
Bos taurus
Babyrousa babyrussa
SSC6_REGION_14
Porcula salvania
Potamochoerus porcus
Phacochoerus africanus
Sus celebensis
Sus scrofa
Sus cebifrons
Sus verrucosus
Sus barbatus
SSC6_REGION_15
SSC6_REGION_15
Bos taurus
Pecari tajacu
Babyrousa babyrussa
Potamochoerus porcus
Phacochoerus africanus
Porcula salvania
Sus cebifrons
Sus celebensis
Sus scrofa
Sus verrucosus
Sus barbatus
SSC6_REGION_16
SSC6_REGION_16
Bos taurus
Pecari tajacu
Potamochoerus porcus
Phacochoerus africanus
Porcula salvania
Babyrousa babyrussa
Sus celebensis
Sus barbatus
Sus verrucosus
SSC6_REGION_18
SSC6_REGION_18
Bos taurus
Pecari tajacu
Porcula salvania
Babyrousa babyrussa
Sus cebifrons
Sus celebensis
Sus barbatus
Sus verrucosus
SSC6_REGION_23
SSC6_REGION_23
Bos taurus
Pecari tajacu
Potamochoerus porcus
Phacochoerus africanus
Babyrousa babyrussa
Porcula salvania
Sus scrofa
Sus cebifrons
Sus celebensis
Sus barbatus
Sus verrucosus
SSC6_REGION_24
SSC6_REGION_24
Bos taurus
Pecari tajacu
Phacochoerus africanus
Potamochoerus porcus
Babyrousa babyrussa
Porcula salvania
Sus scrofa
Sus verrucosus
Sus barbatus
SSC6_REGION_26
Bos taurus
Pecari tajacu
Babyrousa babyrussa
Potamochoerus porcus
Phacochoerus africanus
Porcula salvania
SSC6_REGION_26
Sus cebifrons
Sus scrofa
Sus celebensis
Sus verrucosus
Sus barbatus
SSC6_REGION_30
Pecari tajacu
Bos taurus
Babyrousa babyrussa
Potamochoerus porcus
Phacochoerus africanus
SSC6_REGION_30
Porcula salvania
Sus cebifrons
Sus barbatus
Sus verrucosus
Sus scrofa
Sus celebensis
SSC6_REGION_31
Bos taurus
Pecari tajacu
SSC6_REGION_31
Phacochoerus africanus
Potamochoerus porcus
Babyrousa babyrussa
Porcula salvania
Sus scrofa
Sus cebifrons
Sus verrucosus
Sus barbatus
Sus celebensis
SSC6_REGION_34
SSC6_REGION_34
Bos taurus
Pecari tajacu
Babyrousa babyrussa
Potamochoerus porcus
Phacochoerus africanus
Porcula salvania
Sus scrofa
Sus celebensis
Sus verrucosus
Sus barbatus
SSC7_REGION_1
Bos taurus
SSC7_REGION_1
Pecari tajacu
Potamochoerus porcus
Babyrousa babyrussa
Sus cebifrons
Phacochoerus africanus
Porcula salvania
Sus scrofa
Sus celebensis
Sus verrucosus
Sus barbatus
SSC7_REGION_4
SSC7_REGION_4
Bos taurus
Pecari tajacu
Babyrousa babyrussa
Phacochoerus africanus
Porcula salvania
Sus cebifrons
Sus celebensis
Sus scrofa
Sus barbatus
Sus verrucosus
SSC7_REGION_9
SSC7_REGION_9
Bos taurus
Pecari tajacu
Porcula salvania
Phacochoerus africanus
Babyrousa babyrussa
Potamochoerus porcus
Sus celebensis
Sus scrofa
Sus verrucosus
Sus barbatus
SSC7_REGION_10
SSC7_REGION_10
Bos taurus
Pecari tajacu
Babyrousa babyrussa
Phacochoerus africanus
Potamochoerus porcus
Porcula salvania
Sus cebifrons
Sus celebensis
Sus scrofa
Sus verrucosus
Sus barbatus
SSC7_REGION_11
SSC7_REGION_11
Bos taurus
Pecari tajacu
Babyrousa babyrussa
Potamochoerus porcus
Phacochoerus africanus
Porcula salvania
Sus barbatus
Sus verrucosus
Sus celebensis
Sus cebifrons
Sus scrofa
SSC7_REGION_14
Bos taurus
Pecari tajacu
Phacochoerus africanus
Babyrousa babyrussa
Potamochoerus porcus
SSC7_REGION_14
Porcula salvania
Sus cebifrons
Sus celebensis
Sus scrofa
Sus barbatus
Sus verrucosus
SSC7_REGION_15
Bos taurus
Pecari tajacu
Babyrousa babyrussa
SSC7_REGION_15
Potamochoerus porcus
Phacochoerus africanus
Porcula salvania
Sus scrofa
Sus celebensis
Sus cebifrons
Sus barbatus
Sus verrucosus
SSC8_REGION_1
Pecari tajacu
Potamochoerus porcus
Porcula salvania
Babyrousa babyrussa
Phacochoerus africanus
SSC8_REGION_1
Sus scrofa
Sus cebifrons
Sus celebensis
Sus verrucosus
Sus barbatus

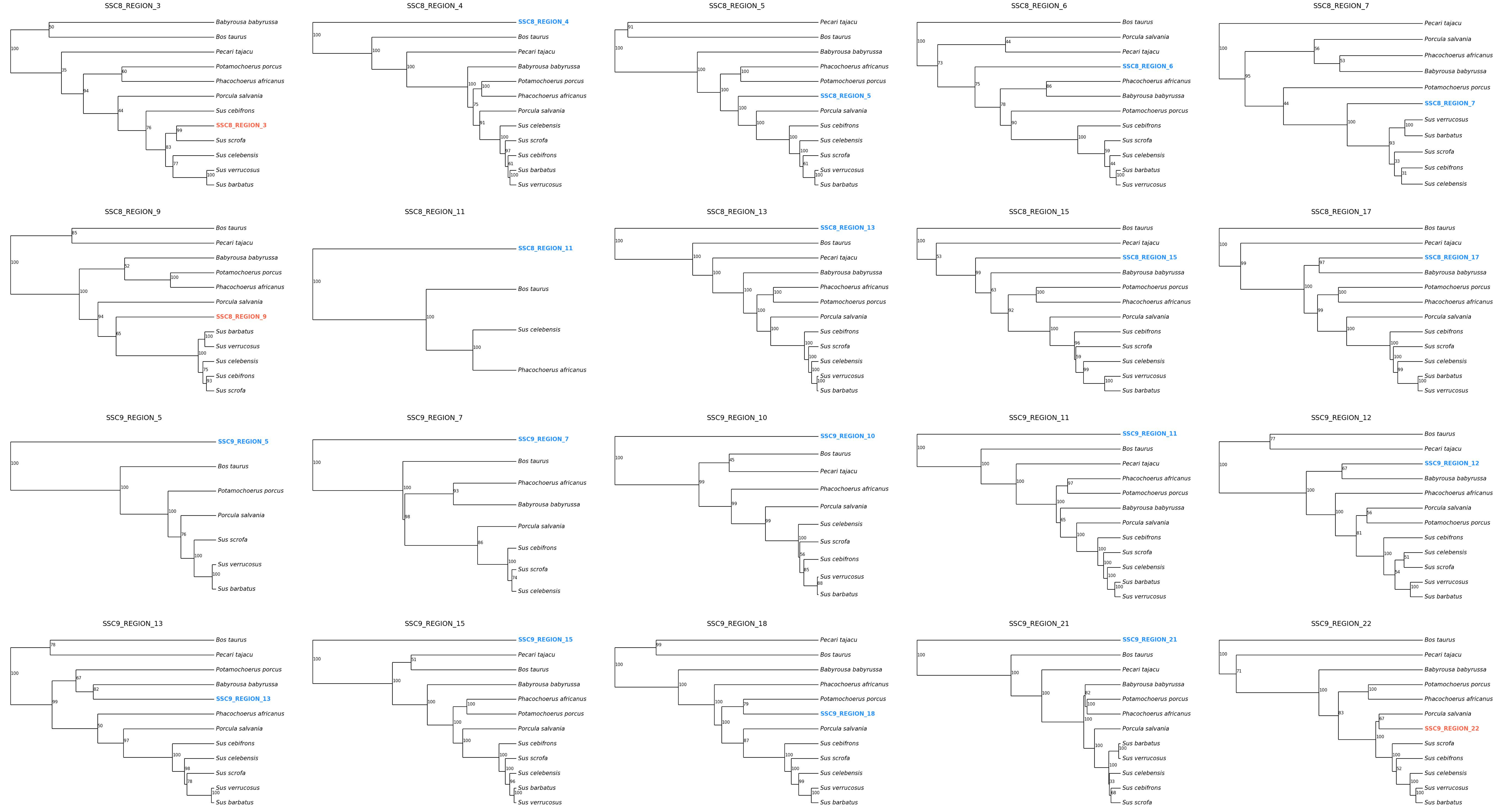
SSC8_REGION_3
Babyrousa babyrussa
Bos taurus
Pecari tajacu
Potamochoerus porcus
Phacochoerus africanus
Porcula salvania
Sus cebifrons
SSC8_REGION_3
Sus scrofa
Sus celebensis
Sus verrucosus
Sus barbatus
SSC8_REGION_4
SSC8_REGION_4
Bos taurus
Pecari tajacu
Babyrousa babyrussa
Potamochoerus porcus
Phacochoerus africanus
Porcula salvania
Sus celebensis
Sus scrofa
Sus cebifrons
Sus barbatus
Sus verrucosus
SSC8_REGION_5
Pecari tajacu
Bos taurus
Babyrousa babyrussa
Phacochoerus africanus
Potamochoerus porcus
SSC8_REGION_5
Porcula salvania
Sus cebifrons
Sus celebensis
Sus scrofa
Sus verrucosus
Sus barbatus
SSC8_REGION_6
Bos taurus
Porcula salvania
Pecari tajacu
SSC8_REGION_6
Phacochoerus africanus
Babyrousa babyrussa
Potamochoerus porcus
Sus cebifrons
Sus scrofa
Sus celebensis
Sus barbatus
Sus verrucosus
SSC8_REGION_7
Pecari tajacu
Porcula salvania
Phacochoerus africanus
Babyrousa babyrussa
Potamochoerus porcus
SSC8_REGION_7
Sus verrucosus
Sus barbatus
Sus scrofa
Sus cebifrons
Sus celebensis
SSC8_REGION_9
Bos taurus
Pecari tajacu
Babyrousa babyrussa
Potamochoerus porcus
Phacochoerus africanus
Porcula salvania
SSC8_REGION_9
Sus barbatus
Sus verrucosus
Sus celebensis
Sus cebifrons
Sus scrofa
SSC8_REGION_11
SSC8_REGION_11
Bos taurus
Sus celebensis
Phacochoerus africanus
SSC8_REGION_13
SSC8_REGION_13
Bos taurus
Pecari tajacu
Babyrousa babyrussa
Phacochoerus africanus
Potamochoerus porcus
Porcula salvania
Sus cebifrons
Sus scrofa
Sus celebensis
Sus verrucosus
Sus barbatus
SSC8_REGION_15
Bos taurus
Pecari tajacu
SSC8_REGION_15
Babyrousa babyrussa
Potamochoerus porcus
Phacochoerus africanus
Porcula salvania
Sus cebifrons
Sus scrofa
Sus celebensis
Sus verrucosus
Sus barbatus
SSC8_REGION_17
Bos taurus
Pecari tajacu
SSC8_REGION_17
Babyrousa babyrussa
Potamochoerus porcus
Phacochoerus africanus
Porcula salvania
Sus cebifrons
Sus scrofa
Sus celebensis
Sus barbatus
Sus verrucosus
SSC9_REGION_5
SSC9_REGION_5
Bos taurus
Potamochoerus porcus
Porcula salvania
Sus scrofa
Sus verrucosus
Sus barbatus
SSC9_REGION_7
SSC9_REGION_7
Bos taurus
Phacochoerus africanus
Babyrousa babyrussa
Porcula salvania
Sus cebifrons
Sus scrofa
Sus celebensis
SSC9_REGION_10
SSC9_REGION_10
Bos taurus
Pecari tajacu
Phacochoerus africanus
Porcula salvania
Sus celebensis
Sus scrofa
Sus cebifrons
Sus verrucosus
Sus barbatus
SSC9_REGION_11
SSC9_REGION_11
Bos taurus
Pecari tajacu
Phacochoerus africanus
Potamochoerus porcus
Babyrousa babyrussa
Porcula salvania
Sus cebifrons
Sus scrofa
Sus celebensis
Sus barbatus
Sus verrucosus
SSC9_REGION_12
Bos taurus
Pecari tajacu
SSC9_REGION_12
Babyrousa babyrussa
Phacochoerus africanus
Porcula salvania
Potamochoerus porcus
Sus cebifrons
Sus celebensis
Sus scrofa
Sus verrucosus
Sus barbatus
SSC9_REGION_13
Bos taurus
Pecari tajacu
Potamochoerus porcus
Babyrousa babyrussa
SSC9_REGION_13
Phacochoerus africanus
Porcula salvania
Sus cebifrons
Sus celebensis
Sus scrofa
Sus verrucosus
Sus barbatus
SSC9_REGION_15
SSC9_REGION_15
Pecari tajacu
Bos taurus
Babyrousa babyrussa
Phacochoerus africanus
Potamochoerus porcus
Porcula salvania
Sus cebifrons
Sus scrofa
Sus celebensis
Sus barbatus
Sus verrucosus
SSC9_REGION_18
Pecari tajacu
Bos taurus
Babyrousa babyrussa
Phacochoerus africanus
Potamochoerus porcus
SSC9_REGION_18
Porcula salvania
Sus cebifrons
Sus scrofa
Sus celebensis
Sus verrucosus
Sus barbatus
SSC9_REGION_21
SSC9_REGION_21
Bos taurus
Pecari tajacu
Babyrousa babyrussa
Potamochoerus porcus
Phacochoerus africanus
Porcula salvania
Sus barbatus
Sus verrucosus
Sus celebensis
Sus cebifrons
Sus scrofa
SSC9_REGION_22
Bos taurus
Pecari tajacu
Babyrousa babyrussa
Potamochoerus porcus
Phacochoerus africanus
Porcula salvania
SSC9_REGION_22
Sus scrofa
Sus cebifrons
Sus celebensis
Sus verrucosus
Sus barbatus

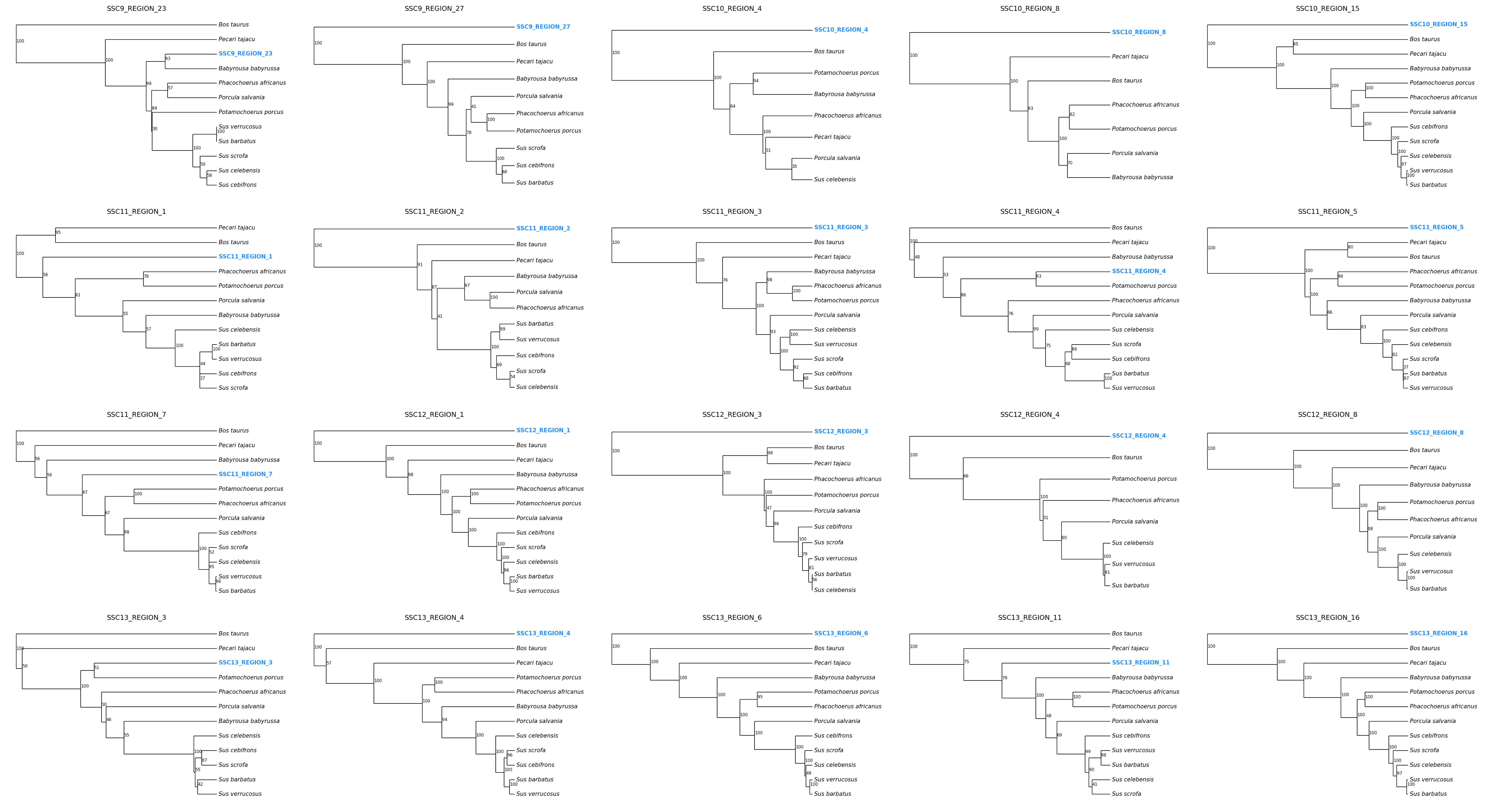
SSC9_REGION_23
Bos taurus
Pecari tajacu
SSC9_REGION_23
Babyrousa babyrussa
Phacochoerus africanus
Porcula salvania
Potamochoerus porcus
Sus verrucosus
Sus barbatus
Sus scrofa
Sus celebensis
Sus cebifrons
SSC9_REGION_27
SSC9_REGION_27
Bos taurus
Pecari tajacu
Babyrousa babyrussa
Porcula salvania
Phacochoerus africanus
Potamochoerus porcus
Sus scrofa
Sus cebifrons
Sus barbatus
SSC10_REGION_4
SSC10_REGION_4
Bos taurus
Potamochoerus porcus
Babyrousa babyrussa
Phacochoerus africanus
Pecari tajacu
Porcula salvania
Sus celebensis
SSC10_REGION_8
SSC10_REGION_8
Pecari tajacu
Bos taurus
Phacochoerus africanus
Potamochoerus porcus
Porcula salvania
Babyrousa babyrussa
SSC10_REGION_15
SSC10_REGION_15
Bos taurus
Pecari tajacu
Babyrousa babyrussa
Potamochoerus porcus
Phacochoerus africanus
Porcula salvania
Sus cebifrons
Sus scrofa
Sus celebensis
Sus verrucosus
Sus barbatus
SSC11_REGION_1
Pecari tajacu
Bos taurus
SSC11_REGION_1
Phacochoerus africanus
Potamochoerus porcus
Porcula salvania
Babyrousa babyrussa
Sus celebensis
Sus barbatus
Sus verrucosus
Sus cebifrons
Sus scrofa
SSC11_REGION_2
SSC11_REGION_2
Bos taurus
Pecari tajacu
Babyrousa babyrussa
Porcula salvania
Phacochoerus africanus
Sus barbatus
Sus verrucosus
Sus cebifrons
Sus scrofa
Sus celebensis
SSC11_REGION_3
SSC11_REGION_3
Bos taurus
Pecari tajacu
Babyrousa babyrussa
Phacochoerus africanus
Potamochoerus porcus
Porcula salvania
Sus celebensis
Sus verrucosus
Sus scrofa
Sus cebifrons
Sus barbatus
SSC11_REGION_4
Bos taurus
Pecari tajacu
Babyrousa babyrussa
SSC11_REGION_4
Potamochoerus porcus
Phacochoerus africanus
Porcula salvania
Sus celebensis
Sus scrofa
Sus cebifrons
Sus barbatus
Sus verrucosus
SSC11_REGION_5
SSC11_REGION_5
Pecari tajacu
Bos taurus
Phacochoerus africanus
Potamochoerus porcus
Babyrousa babyrussa
Porcula salvania
Sus cebifrons
Sus celebensis
Sus scrofa
Sus barbatus
Sus verrucosus
SSC11_REGION_7
Bos taurus
Pecari tajacu
Babyrousa babyrussa
SSC11_REGION_7
Potamochoerus porcus
Phacochoerus africanus
Porcula salvania
Sus cebifrons
Sus scrofa
Sus celebensis
Sus verrucosus
Sus barbatus
SSC12_REGION_1
SSC12_REGION_1
Bos taurus
Pecari tajacu
Babyrousa babyrussa
Phacochoerus africanus
Potamochoerus porcus
Porcula salvania
Sus cebifrons
Sus scrofa
Sus celebensis
Sus barbatus
Sus verrucosus
SSC12_REGION_3
SSC12_REGION_3
Bos taurus
Pecari tajacu
Phacochoerus africanus
Potamochoerus porcus
Porcula salvania
Sus cebifrons
Sus scrofa
Sus verrucosus
Sus barbatus
Sus celebensis
SSC12_REGION_4
SSC12_REGION_4
Bos taurus
Potamochoerus porcus
Phacochoerus africanus
Porcula salvania
Sus celebensis
Sus verrucosus
Sus barbatus
SSC12_REGION_8
SSC12_REGION_8
Bos taurus
Pecari tajacu
Babyrousa babyrussa
Potamochoerus porcus
Phacochoerus africanus
Porcula salvania
Sus celebensis
Sus verrucosus
Sus barbatus
SSC13_REGION_3
Bos taurus
Pecari tajacu
SSC13_REGION_3
Potamochoerus porcus
Phacochoerus africanus
Porcula salvania
Babyrousa babyrussa
Sus celebensis
Sus cebifrons
Sus scrofa
Sus barbatus
Sus verrucosus
SSC13_REGION_4
SSC13_REGION_4
Bos taurus
Pecari tajacu
Potamochoerus porcus
Phacochoerus africanus
Babyrousa babyrussa
Porcula salvania
Sus celebensis
Sus scrofa
Sus cebifrons
Sus barbatus
Sus verrucosus
SSC13_REGION_6
SSC13_REGION_6
Bos taurus
Pecari tajacu
Babyrousa babyrussa
Potamochoerus porcus
Phacochoerus africanus
Porcula salvania
Sus cebifrons
Sus scrofa
Sus celebensis
Sus verrucosus
Sus barbatus
SSC13_REGION_11
Bos taurus
Pecari tajacu
SSC13_REGION_11
Babyrousa babyrussa
Phacochoerus africanus
Potamochoerus porcus
Porcula salvania
Sus cebifrons
Sus verrucosus
Sus barbatus
Sus celebensis
Sus scrofa
SSC13_REGION_16
SSC13_REGION_16
Bos taurus
Pecari tajacu
Babyrousa babyrussa
Potamochoerus porcus
Phacochoerus africanus
Porcula salvania
Sus cebifrons
Sus scrofa
Sus celebensis
Sus verrucosus
Sus barbatus

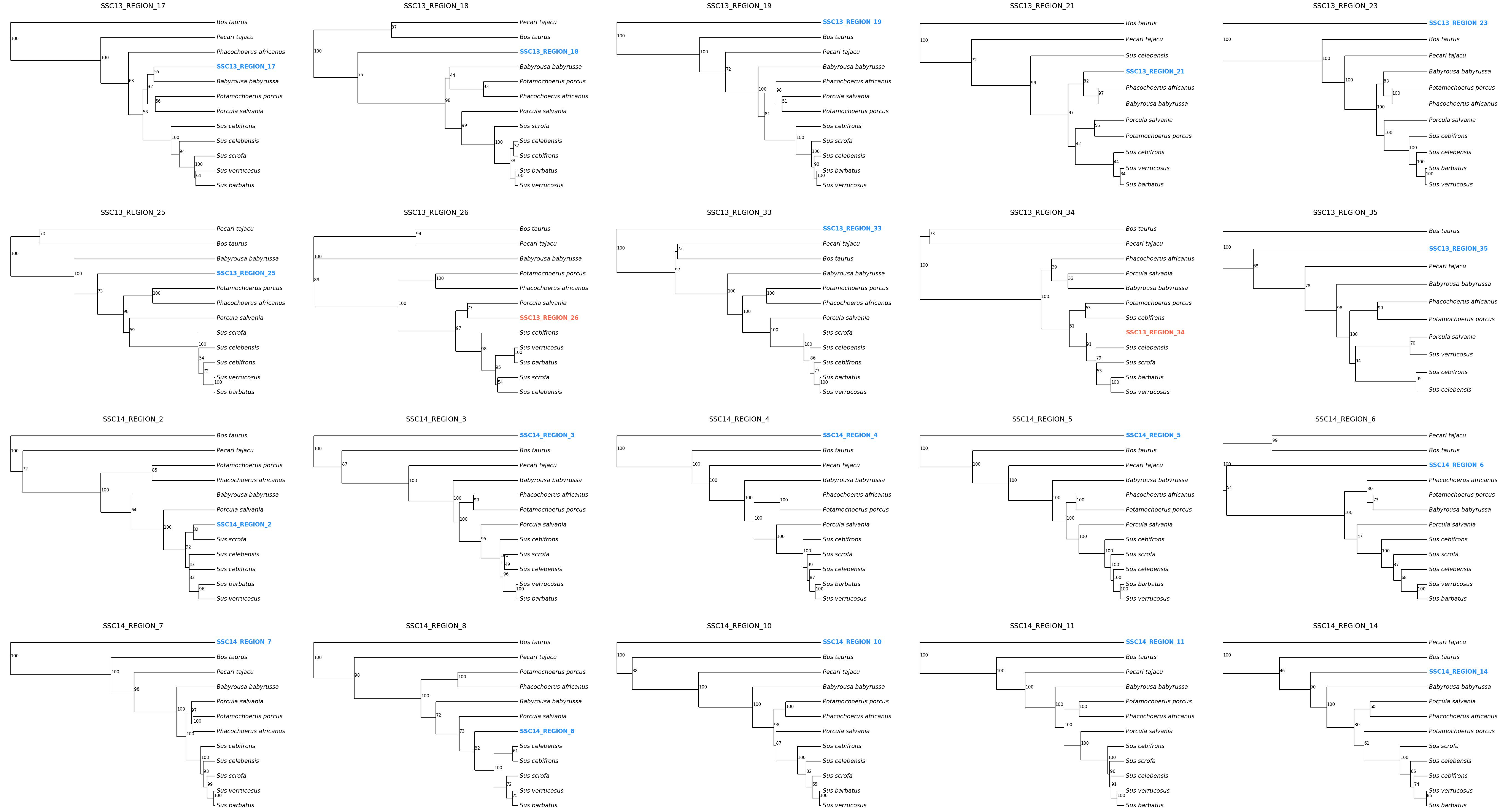
SSC13_REGION_17
Bos taurus
Pecari tajacu
Phacochoerus africanus
SSC13_REGION_17
Babyrousa babyrussa
Potamochoerus porcus
Porcula salvania
Sus cebifrons
Sus celebensis
Sus scrofa
Sus verrucosus
Sus barbatus
SSC13_REGION_18
Pecari tajacu
Bos taurus
SSC13_REGION_18
Babyrousa babyrussa
Potamochoerus porcus
Phacochoerus africanus
Porcula salvania
Sus scrofa
Sus celebensis
Sus cebifrons
Sus barbatus
Sus verrucosus
SSC13_REGION_19
SSC13_REGION_19
Bos taurus
Pecari tajacu
Babyrousa babyrussa
Phacochoerus africanus
Porcula salvania
Potamochoerus porcus
Sus cebifrons
Sus scrofa
Sus celebensis
Sus barbatus
Sus verrucosus
SSC13_REGION_21
Bos taurus
Pecari tajacu
Sus celebensis
SSC13_REGION_21
Phacochoerus africanus
Babyrousa babyrussa
Porcula salvania
Potamochoerus porcus
Sus cebifrons
Sus verrucosus
Sus barbatus
SSC13_REGION_23
SSC13_REGION_23
Bos taurus
Pecari tajacu
Babyrousa babyrussa
Potamochoerus porcus
Phacochoerus africanus
Porcula salvania
Sus cebifrons
Sus celebensis
Sus barbatus
Sus verrucosus
SSC13_REGION_25
Pecari tajacu
Bos taurus
Babyrousa babyrussa
SSC13_REGION_25
Potamochoerus porcus
Phacochoerus africanus
Porcula salvania
Sus scrofa
Sus celebensis
Sus cebifrons
Sus verrucosus
Sus barbatus
SSC13_REGION_26
Bos taurus
Pecari tajacu
Babyrousa babyrussa
Potamochoerus porcus
Phacochoerus africanus
Porcula salvania
SSC13_REGION_26
Sus cebifrons
Sus verrucosus
Sus barbatus
Sus scrofa
Sus celebensis
SSC13_REGION_33
SSC13_REGION_33
Pecari tajacu
Bos taurus
Babyrousa babyrussa
Potamochoerus porcus
Phacochoerus africanus
Porcula salvania
Sus scrofa
Sus celebensis
Sus cebifrons
Sus barbatus
Sus verrucosus
SSC13_REGION_34
Bos taurus
Pecari tajacu
Phacochoerus africanus
Porcula salvania
Babyrousa babyrussa
Potamochoerus porcus
Sus cebifrons
SSC13_REGION_34
Sus celebensis
Sus scrofa
Sus barbatus
Sus verrucosus
SSC13_REGION_35
Bos taurus
SSC13_REGION_35
Pecari tajacu
Babyrousa babyrussa
Phacochoerus africanus
Potamochoerus porcus
Porcula salvania
Sus verrucosus
Sus cebifrons
Sus celebensis
SSC14_REGION_2
Bos taurus
Pecari tajacu
Potamochoerus porcus
Phacochoerus africanus
Babyrousa babyrussa
Porcula salvania
SSC14_REGION_2
Sus scrofa
Sus celebensis
Sus cebifrons
Sus barbatus
Sus verrucosus
SSC14_REGION_3
SSC14_REGION_3
Bos taurus
Pecari tajacu
Babyrousa babyrussa
Phacochoerus africanus
Potamochoerus porcus
Porcula salvania
Sus cebifrons
Sus scrofa
Sus celebensis
Sus verrucosus
Sus barbatus
SSC14_REGION_4
SSC14_REGION_4
Bos taurus
Pecari tajacu
Babyrousa babyrussa
Phacochoerus africanus
Potamochoerus porcus
Porcula salvania
Sus cebifrons
Sus scrofa
Sus celebensis
Sus barbatus
Sus verrucosus
SSC14_REGION_5
SSC14_REGION_5
Bos taurus
Pecari tajacu
Babyrousa babyrussa
Phacochoerus africanus
Potamochoerus porcus
Porcula salvania
Sus cebifrons
Sus scrofa
Sus celebensis
Sus barbatus
Sus verrucosus
SSC14_REGION_6
Pecari tajacu
Bos taurus
SSC14_REGION_6
Phacochoerus africanus
Potamochoerus porcus
Babyrousa babyrussa
Porcula salvania
Sus cebifrons
Sus scrofa
Sus celebensis
Sus verrucosus
Sus barbatus
SSC14_REGION_7
SSC14_REGION_7
Bos taurus
Pecari tajacu
Babyrousa babyrussa
Porcula salvania
Potamochoerus porcus
Phacochoerus africanus
Sus cebifrons
Sus celebensis
Sus scrofa
Sus verrucosus
Sus barbatus
SSC14_REGION_8
Bos taurus
Pecari tajacu
Potamochoerus porcus
Phacochoerus africanus
Babyrousa babyrussa
Porcula salvania
SSC14_REGION_8
Sus celebensis
Sus cebifrons
Sus scrofa
Sus verrucosus
Sus barbatus
SSC14_REGION_10
SSC14_REGION_10
Bos taurus
Pecari tajacu
Babyrousa babyrussa
Potamochoerus porcus
Phacochoerus africanus
Porcula salvania
Sus cebifrons
Sus celebensis
Sus scrofa
Sus barbatus
Sus verrucosus
SSC14_REGION_11
SSC14_REGION_11
Bos taurus
Pecari tajacu
Babyrousa babyrussa
Potamochoerus porcus
Phacochoerus africanus
Porcula salvania
Sus cebifrons
Sus scrofa
Sus celebensis
Sus verrucosus
Sus barbatus
SSC14_REGION_14
Pecari tajacu
Bos taurus
SSC14_REGION_14
Babyrousa babyrussa
Porcula salvania
Phacochoerus africanus
Potamochoerus porcus
Sus scrofa
Sus celebensis
Sus cebifrons
Sus verrucosus
Sus barbatus

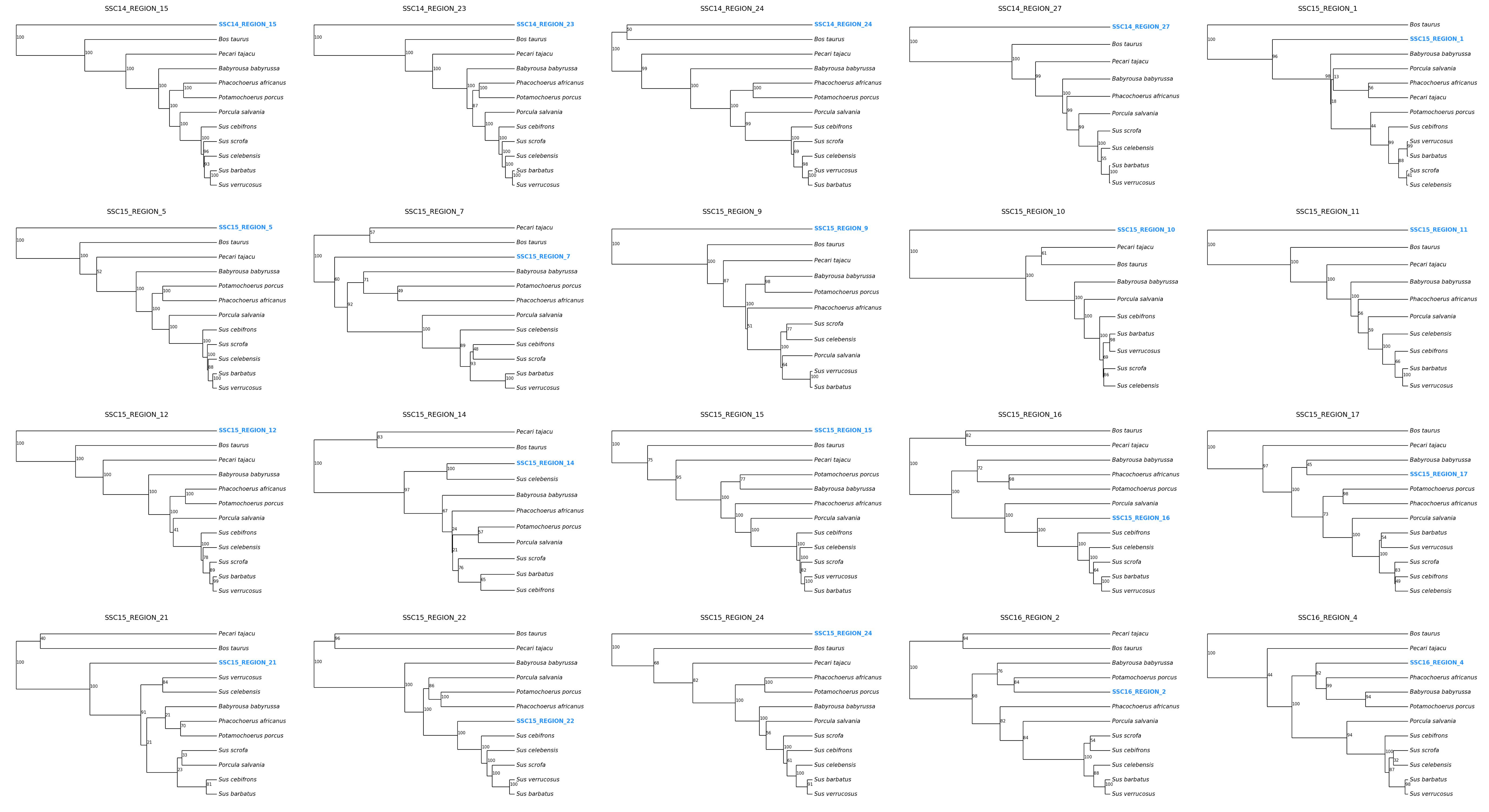

SSC14_REGION_15
SSC14_REGION_15
Bos taurus
Pecari tajacu
Babyrousa babyrussa
Phacochoerus africanus
Potamochoerus porcus
Porcula salvania
Sus cebifrons
Sus scrofa
Sus celebensis
Sus barbatus
Sus verrucosus
SSC14_REGION_23
SSC14_REGION_23
Bos taurus
Pecari tajacu
Babyrousa babyrussa
Phacochoerus africanus
Potamochoerus porcus
Porcula salvania
Sus cebifrons
Sus scrofa
Sus celebensis
Sus barbatus
Sus verrucosus
SSC14_REGION_24
SSC14_REGION_24
Bos taurus
Pecari tajacu
Babyrousa babyrussa
Phacochoerus africanus
Potamochoerus porcus
Porcula salvania
Sus cebifrons
Sus scrofa
Sus celebensis
Sus verrucosus
Sus barbatus
SSC14_REGION_27
SSC14_REGION_27
Bos taurus
Pecari tajacu
Babyrousa babyrussa
Phacochoerus africanus
Porcula salvania
Sus scrofa
Sus celebensis
Sus barbatus
Sus verrucosus
SSC15_REGION_1
Bos taurus
SSC15_REGION_1
Babyrousa babyrussa
Porcula salvania
Phacochoerus africanus
Pecari tajacu
Potamochoerus porcus
Sus cebifrons
Sus verrucosus
Sus barbatus
Sus scrofa
Sus celebensis
SSC15_REGION_5
SSC15_REGION_5
Bos taurus
Pecari tajacu
Babyrousa babyrussa
Potamochoerus porcus
Phacochoerus africanus
Porcula salvania
Sus cebifrons
Sus scrofa
Sus celebensis
Sus barbatus
Sus verrucosus
SSC15_REGION_7
Pecari tajacu
Bos taurus
SSC15_REGION_7
Babyrousa babyrussa
Potamochoerus porcus
Phacochoerus africanus
Porcula salvania
Sus celebensis
Sus cebifrons
Sus scrofa
Sus barbatus
Sus verrucosus
SSC15_REGION_9
SSC15_REGION_9
Bos taurus
Pecari tajacu
Babyrousa babyrussa
Potamochoerus porcus
Phacochoerus africanus
Sus scrofa
Sus celebensis
Porcula salvania
Sus verrucosus
Sus barbatus
SSC15_REGION_10
SSC15_REGION_10
Pecari tajacu
Bos taurus
Babyrousa babyrussa
Porcula salvania
Sus cebifrons
Sus barbatus
Sus verrucosus
Sus scrofa
Sus celebensis
SSC15_REGION_11
SSC15_REGION_11
Bos taurus
Pecari tajacu
Babyrousa babyrussa
Phacochoerus africanus
Porcula salvania
Sus celebensis
Sus cebifrons
Sus barbatus
Sus verrucosus
SSC15_REGION_12
SSC15_REGION_12
Bos taurus
Pecari tajacu
Babyrousa babyrussa
Phacochoerus africanus
Potamochoerus porcus
Porcula salvania
Sus cebifrons
Sus celebensis
Sus scrofa
Sus barbatus
Sus verrucosus
SSC15_REGION_14
Pecari tajacu
Bos taurus
SSC15_REGION_14
Sus celebensis
Babyrousa babyrussa
Phacochoerus africanus
Potamochoerus porcus
Porcula salvania
Sus scrofa
Sus barbatus
Sus cebifrons
SSC15_REGION_15
SSC15_REGION_15
Bos taurus
Pecari tajacu
Potamochoerus porcus
Babyrousa babyrussa
Phacochoerus africanus
Porcula salvania
Sus cebifrons
Sus celebensis
Sus scrofa
Sus verrucosus
Sus barbatus
SSC15_REGION_16
Bos taurus
Pecari tajacu
Babyrousa babyrussa
Phacochoerus africanus
Potamochoerus porcus
Porcula salvania
SSC15_REGION_16
Sus cebifrons
Sus celebensis
Sus scrofa
Sus barbatus
Sus verrucosus
SSC15_REGION_17
Bos taurus
Pecari tajacu
Babyrousa babyrussa
SSC15_REGION_17
Potamochoerus porcus
Phacochoerus africanus
Porcula salvania
Sus barbatus
Sus verrucosus
Sus scrofa
Sus cebifrons
Sus celebensis
SSC15_REGION_21
Pecari tajacu
Bos taurus
SSC15_REGION_21
Sus verrucosus
Sus celebensis
Babyrousa babyrussa
Phacochoerus africanus
Potamochoerus porcus
Sus scrofa
Porcula salvania
Sus cebifrons
Sus barbatus
SSC15_REGION_22
Bos taurus
Pecari tajacu
Babyrousa babyrussa
Porcula salvania
Potamochoerus porcus
Phacochoerus africanus
SSC15_REGION_22
Sus cebifrons
Sus celebensis
Sus scrofa
Sus verrucosus
Sus barbatus
SSC15_REGION_24
SSC15_REGION_24
Bos taurus
Pecari tajacu
Phacochoerus africanus
Potamochoerus porcus
Babyrousa babyrussa
Porcula salvania
Sus scrofa
Sus cebifrons
Sus celebensis
Sus barbatus
Sus verrucosus
SSC16_REGION_2
Pecari tajacu
Bos taurus
Babyrousa babyrussa
Potamochoerus porcus
SSC16_REGION_2
Phacochoerus africanus
Porcula salvania
Sus scrofa
Sus cebifrons
Sus celebensis
Sus barbatus
Sus verrucosus
SSC16_REGION_4
Bos taurus
Pecari tajacu
SSC16_REGION_4
Phacochoerus africanus
Babyrousa babyrussa
Potamochoerus porcus
Porcula salvania
Sus cebifrons
Sus scrofa
Sus celebensis
Sus barbatus
Sus verrucosus

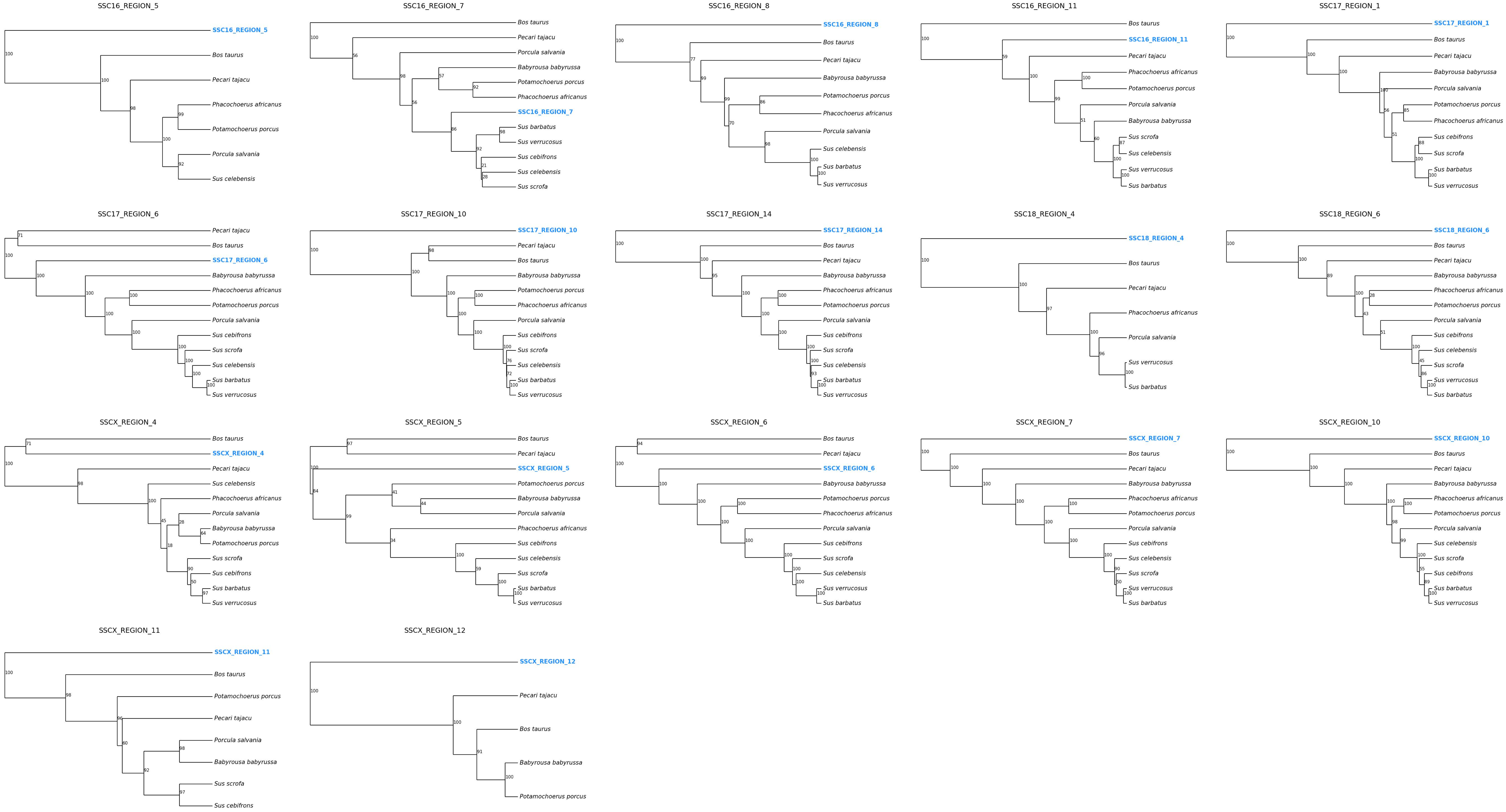
SSC16_REGION_5
SSC16_REGION_5
Bos taurus
Pecari tajacu
Phacochoerus africanus
Potamochoerus porcus
Porcula salvania
Sus celebensis
SSC16_REGION_7
Bos taurus
Pecari tajacu
Porcula salvania
Babyrousa babyrussa
Potamochoerus porcus
Phacochoerus africanus
SSC16_REGION_7
Sus barbatus
Sus verrucosus
Sus cebifrons
Sus celebensis
Sus scrofa
SSC16_REGION_8
SSC16_REGION_8
Bos taurus
Pecari tajacu
Babyrousa babyrussa
Potamochoerus porcus
Phacochoerus africanus
Porcula salvania
Sus celebensis
Sus barbatus
Sus verrucosus
SSC16_REGION_11
Bos taurus
SSC16_REGION_11
Pecari tajacu
Phacochoerus africanus
Potamochoerus porcus
Porcula salvania
Babyrousa babyrussa
Sus scrofa
Sus celebensis
Sus verrucosus
Sus barbatus
SSC17_REGION_1
SSC17_REGION_1
Bos taurus
Pecari tajacu
Babyrousa babyrussa
Porcula salvania
Potamochoerus porcus
Phacochoerus africanus
Sus cebifrons
Sus scrofa
Sus barbatus
Sus verrucosus
SSC17_REGION_6
Pecari tajacu
Bos taurus
SSC17_REGION_6
Babyrousa babyrussa
Phacochoerus africanus
Potamochoerus porcus
Porcula salvania
Sus cebifrons
Sus scrofa
Sus celebensis
Sus barbatus
Sus verrucosus
SSC17_REGION_10
SSC17_REGION_10
Pecari tajacu
Bos taurus
Babyrousa babyrussa
Potamochoerus porcus
Phacochoerus africanus
Porcula salvania
Sus cebifrons
Sus scrofa
Sus celebensis
Sus barbatus
Sus verrucosus
SSC17_REGION_14
SSC17_REGION_14
Bos taurus
Pecari tajacu
Babyrousa babyrussa
Phacochoerus africanus
Potamochoerus porcus
Porcula salvania
Sus cebifrons
Sus scrofa
Sus celebensis
Sus barbatus
Sus verrucosus
SSC18_REGION_4
SSC18_REGION_4
Bos taurus
Pecari tajacu
Phacochoerus africanus
Porcula salvania
Sus verrucosus
Sus barbatus
SSC18_REGION_6
SSC18_REGION_6
Bos taurus
Pecari tajacu
Babyrousa babyrussa
Phacochoerus africanus
Potamochoerus porcus
Porcula salvania
Sus cebifrons
Sus celebensis
Sus scrofa
Sus verrucosus
Sus barbatus
SSCX_REGION_4
Bos taurus
SSCX_REGION_4
Pecari tajacu
Sus celebensis
Phacochoerus africanus
Porcula salvania
Babyrousa babyrussa
Potamochoerus porcus
Sus scrofa
Sus cebifrons
Sus barbatus
Sus verrucosus
SSCX_REGION_5
Bos taurus
Pecari tajacu
SSCX_REGION_5
Potamochoerus porcus
Babyrousa babyrussa
Porcula salvania
Phacochoerus africanus
Sus cebifrons
Sus celebensis
Sus scrofa
Sus barbatus
Sus verrucosus
SSCX_REGION_6
Bos taurus
Pecari tajacu
SSCX_REGION_6
Babyrousa babyrussa
Potamochoerus porcus
Phacochoerus africanus
Porcula salvania
Sus cebifrons
Sus scrofa
Sus celebensis
Sus verrucosus
Sus barbatus
SSCX_REGION_7
SSCX_REGION_7
Bos taurus
Pecari tajacu
Babyrousa babyrussa
Phacochoerus africanus
Potamochoerus porcus
Porcula salvania
Sus cebifrons
Sus celebensis
Sus scrofa
Sus verrucosus
Sus barbatus
SSCX_REGION_10
SSCX_REGION_10
Bos taurus
Pecari tajacu
Babyrousa babyrussa
Phacochoerus africanus
Potamochoerus porcus
Porcula salvania
Sus celebensis
Sus scrofa
Sus cebifrons
Sus barbatus
Sus verrucosus
SSCX_REGION_11
SSCX_REGION_11
Bos taurus
Potamochoerus porcus
Pecari tajacu
Porcula salvania
Babyrousa babyrussa
Sus scrofa
Sus cebifrons
SSCX_REGION_12
SSCX_REGION_12
Pecari tajacu
Bos taurus
Babyrousa babyrussa
Potamochoerus porcus
