# Supplementary material for: A comprehensive atlas of nuclear sequences of mitochondrial origin (NUMT) inserted into the pig genome
Source: Genet Sel Evol. 2024 Sep 16;56:64. doi: 10.1186/s12711-024-00930-6 (PMC11403998; doi:10.1186/s12711-024-00930-6)
Supplement: Supplementary file 5 — Additional file 5: Table S17. Annotation of the Sscrofa11.1 genome version with NUMT regions in GFF format. The nine columns in the file are based on the standard format for a GFF file. The second column indicates the NUMT source; the third column indicates the feature type name. [file 12711_2024_930_MOESM5_ESM.docx]

Additional file 5

Supporting information

**A comprehensive atlas of mitochondrial DNA insertions in the nuclear genome of the pig**

Matteo Bolner, Samuele Bovo, Mohamad Ballan, Giuseppina Schiavo, Valeria Taurisano, Anisa Ribani, Francesca Bertolini and Luca Fontanesi

**Table of content**

**Additional file 5: Table S17. Annotation of the Sscrofa11.1 genome version with NUMT regions in GFF format.**

The nine columns in the file are based on the standard format for a GFF file. The second column indicates the NUMT source (assembly pipeline or WGS pipeline for novel NUMTs); the third column indicates the feature type name (numt region or numt region insertion breakpoints for NUMTs not present in Sscrofa11.1)

| SSC1 | assembly_numt_pipeline | numt_region | 9116246 | 9116400 | . | . | . | . |
| --- | --- | --- | --- | --- | --- | --- | --- | --- |
| SSC1 | assembly_numt_pipeline | numt_region | 15370329 | 15370522 | . | . | . | . |
| SSC1 | assembly_numt_pipeline | numt_region | 41639514 | 41639554 | . | . | . | . |
| SSC1 | assembly_numt_pipeline | numt_region | 48421321 | 48421484 | . | . | . | . |
| SSC1 | assembly_numt_pipeline | numt_region | 53053504 | 53061734 | . | . | . | . |
| SSC1 | assembly_numt_pipeline | numt_region | 57842422 | 57842516 | . | . | . | . |
| SSC1 | assembly_numt_pipeline | numt_region | 73772798 | 73774755 | . | . | . | . |
| SSC1 | assembly_numt_pipeline | numt_region | 73803401 | 73808956 | . | . | . | . |
| SSC1 | assembly_numt_pipeline | numt_region | 82390369 | 82393818 | . | . | . | . |
| SSC1 | assembly_numt_pipeline | numt_region | 89603553 | 89603741 | . | . | . | . |
| SSC1 | assembly_numt_pipeline | numt_region | 91261377 | 91261486 | . | . | . | . |
| SSC1 | assembly_numt_pipeline | numt_region | 97055913 | 97056044 | . | . | . | . |
| SSC1 | assembly_numt_pipeline | numt_region | 112627552 | 112627955 | . | . | . | . |
| SSC1 | assembly_numt_pipeline | numt_region | 122058087 | 122058189 | . | . | . | . |
| SSC1 | assembly_numt_pipeline | numt_region | 144294137 | 144294174 | . | . | . | . |
| SSC1 | assembly_numt_pipeline | numt_region | 145350770 | 145350810 | . | . | . | . |
| SSC1 | assembly_numt_pipeline | numt_region | 148384252 | 148384335 | . | . | . | . |
| SSC1 | assembly_numt_pipeline | numt_region | 151545123 | 151545192 | . | . | . | . |
| SSC1 | assembly_numt_pipeline | numt_region | 152918446 | 152918515 | . | . | . | . |
| SSC1 | assembly_numt_pipeline | numt_region | 156505302 | 156505343 | . | . | . | . |
| SSC1 | assembly_numt_pipeline | numt_region | 158549815 | 158550074 | . | . | . | . |
| SSC1 | assembly_numt_pipeline | numt_region | 169767032 | 169767236 | . | . | . | . |
| SSC1 | assembly_numt_pipeline | numt_region | 212908909 | 212908946 | . | . | . | . |
| SSC1 | assembly_numt_pipeline | numt_region | 219902528 | 219902692 | . | . | . | . |
| SSC1 | assembly_numt_pipeline | numt_region | 221758502 | 221758661 | . | . | . | . |
| SSC1 | assembly_numt_pipeline | numt_region | 222478621 | 222478697 | . | . | . | . |
| SSC1 | assembly_numt_pipeline | numt_region | 230359862 | 230359903 | . | . | . | . |
| SSC1 | assembly_numt_pipeline | numt_region | 239443509 | 239443566 | . | . | . | . |
| SSC1 | assembly_numt_pipeline | numt_region | 250448909 | 250448988 | . | . | . | . |
| SSC1 | assembly_numt_pipeline | numt_region | 259468892 | 259468931 | . | . | . | . |
| SSC1 | assembly_numt_pipeline | numt_region | 268251906 | 268251972 | . | . | . | . |
| SSC1 | assembly_numt_pipeline | numt_region | 273296367 | 273296462 | . | . | . | . |
| SSC2 | assembly_numt_pipeline | numt_region | 19403552 | 19404557 | . | . | . | . |
| SSC2 | assembly_numt_pipeline | numt_region | 23652712 | 23653409 | . | . | . | . |
| SSC2 | assembly_numt_pipeline | numt_region | 38781558 | 38781798 | . | . | . | . |
| SSC2 | assembly_numt_pipeline | numt_region | 46516849 | 46516905 | . | . | . | . |
| SSC2 | assembly_numt_pipeline | numt_region | 56023512 | 56024492 | . | . | . | . |
| SSC2 | assembly_numt_pipeline | numt_region | 67385848 | 67385920 | . | . | . | . |
| SSC2 | assembly_numt_pipeline | numt_region | 82248552 | 82271972 | . | . | . | . |
| SSC2 | assembly_numt_pipeline | numt_region | 82362305 | 82362651 | . | . | . | . |
| SSC2 | assembly_numt_pipeline | numt_region | 84718061 | 84718249 | . | . | . | . |
| SSC2 | assembly_numt_pipeline | numt_region | 93844373 | 93849155 | . | . | . | . |
| SSC2 | assembly_numt_pipeline | numt_region | 105301968 | 105302029 | . | . | . | . |
| SSC2 | assembly_numt_pipeline | numt_region | 109524181 | 109524255 | . | . | . | . |
| SSC2 | assembly_numt_pipeline | numt_region | 115563169 | 115574329 | . | . | . | . |
| SSC2 | assembly_numt_pipeline | numt_region | 117707745 | 117707801 | . | . | . | . |
| SSC2 | assembly_numt_pipeline | numt_region | 131565600 | 131567016 | . | . | . | . |
| SSC2 | assembly_numt_pipeline | numt_region | 133812981 | 133813107 | . | . | . | . |
| SSC2 | assembly_numt_pipeline | numt_region_insertion_breakpoints | 149841871 | 149841882 | . | . | . | . |
| SSC2 | assembly_numt_pipeline | numt_region | 151909815 | 151921322 | . | . | . | . |
| SSC3 | assembly_numt_pipeline | numt_region | 21597 | 44900 | . | . | . | . |
| SSC3 | assembly_numt_pipeline | numt_region | 18128440 | 18128482 | . | . | . | . |
| SSC3 | assembly_numt_pipeline | numt_region | 28987573 | 28987657 | . | . | . | . |
| SSC3 | assembly_numt_pipeline | numt_region | 34560818 | 34560946 | . | . | . | . |
| SSC3 | assembly_numt_pipeline | numt_region | 40460053 | 40460152 | . | . | . | . |
| SSC3 | assembly_numt_pipeline | numt_region | 43151499 | 43169883 | . | . | . | . |
| SSC3 | assembly_numt_pipeline | numt_region | 43734553 | 43734611 | . | . | . | . |
| SSC3 | assembly_numt_pipeline | numt_region | 44071627 | 44071825 | . | . | . | . |
| SSC3 | assembly_numt_pipeline | numt_region | 57062894 | 57063249 | . | . | . | . |
| SSC3 | assembly_numt_pipeline | numt_region | 67736786 | 67737409 | . | . | . | . |
| SSC3 | assembly_numt_pipeline | numt_region | 78374784 | 78383703 | . | . | . | . |
| SSC3 | assembly_numt_pipeline | numt_region | 96623125 | 96623164 | . | . | . | . |
| SSC3 | assembly_numt_pipeline | numt_region | 100324002 | 100324185 | . | . | . | . |
| SSC3 | assembly_numt_pipeline | numt_region | 102883337 | 102895312 | . | . | . | . |
| SSC3 | assembly_numt_pipeline | numt_region | 110754830 | 110754875 | . | . | . | . |
| SSC3 | assembly_numt_pipeline | numt_region | 120898148 | 120898323 | . | . | . | . |
| SSC3 | assembly_numt_pipeline | numt_region | 123499171 | 123499306 | . | . | . | . |
| SSC4 | assembly_numt_pipeline | numt_region_insertion_breakpoints | 16031905 | 16031907 | . | . | . | . |
| SSC4 | assembly_numt_pipeline | numt_region | 25799135 | 25799482 | . | . | . | . |
| SSC4 | assembly_numt_pipeline | numt_region | 29940345 | 29940409 | . | . | . | . |
| SSC4 | assembly_numt_pipeline | numt_region | 39630581 | 39630747 | . | . | . | . |
| SSC4 | assembly_numt_pipeline | numt_region | 53952873 | 53954297 | . | . | . | . |
| SSC4 | assembly_numt_pipeline | numt_region | 57934985 | 57935224 | . | . | . | . |
| SSC4 | assembly_numt_pipeline | numt_region | 66811796 | 66811948 | . | . | . | . |
| SSC4 | assembly_numt_pipeline | numt_region | 68194819 | 68194922 | . | . | . | . |
| SSC4 | assembly_numt_pipeline | numt_region | 78043891 | 78044021 | . | . | . | . |
| SSC4 | assembly_numt_pipeline | numt_region | 81460964 | 81461100 | . | . | . | . |
| SSC4 | assembly_numt_pipeline | numt_region | 89935499 | 89935893 | . | . | . | . |
| SSC4 | assembly_numt_pipeline | numt_region | 93743166 | 93743200 | . | . | . | . |
| SSC4 | assembly_numt_pipeline | numt_region | 107040213 | 107040502 | . | . | . | . |
| SSC4 | assembly_numt_pipeline | numt_region | 108441076 | 108441249 | . | . | . | . |
| SSC4 | assembly_numt_pipeline | numt_region | 114982660 | 114982878 | . | . | . | . |
| SSC4 | assembly_numt_pipeline | numt_region | 127921847 | 127922367 | . | . | . | . |
| SSC4 | assembly_numt_pipeline | numt_region | 130866364 | 130866679 | . | . | . | . |
| SSC4 | assembly_numt_pipeline | numt_region | 130906186 | 130909826 | . | . | . | . |
| SSC5 | assembly_numt_pipeline | numt_region_insertion_breakpoints | 23289731 | 23289744 | . | . | . | . |
| SSC5 | assembly_numt_pipeline | numt_region | 24433186 | 24433277 | . | . | . | . |
| SSC5 | assembly_numt_pipeline | numt_region | 29314575 | 29314639 | . | . | . | . |
| SSC5 | assembly_numt_pipeline | numt_region | 38195486 | 38195547 | . | . | . | . |
| SSC5 | assembly_numt_pipeline | numt_region | 41779883 | 41779933 | . | . | . | . |
| SSC5 | assembly_numt_pipeline | numt_region | 48601351 | 48605401 | . | . | . | . |
| SSC5 | assembly_numt_pipeline | numt_region | 50183047 | 50183230 | . | . | . | . |
| SSC5 | assembly_numt_pipeline | numt_region | 52316852 | 52316927 | . | . | . | . |
| SSC5 | assembly_numt_pipeline | numt_region | 57080249 | 57080326 | . | . | . | . |
| SSC5 | assembly_numt_pipeline | numt_region | 57116951 | 57117128 | . | . | . | . |
| SSC5 | assembly_numt_pipeline | numt_region | 61211070 | 61211254 | . | . | . | . |
| SSC5 | assembly_numt_pipeline | numt_region | 61440561 | 61440622 | . | . | . | . |
| SSC5 | assembly_numt_pipeline | numt_region | 65471084 | 65471396 | . | . | . | . |
| SSC5 | assembly_numt_pipeline | numt_region | 67080447 | 67080512 | . | . | . | . |
| SSC5 | assembly_numt_pipeline | numt_region | 74793770 | 74793991 | . | . | . | . |
| SSC5 | assembly_numt_pipeline | numt_region | 81199821 | 81199893 | . | . | . | . |
| SSC6 | assembly_numt_pipeline | numt_region | 339982 | 340224 | . | . | . | . |
| SSC6 | assembly_numt_pipeline | numt_region | 8607412 | 8607488 | . | . | . | . |
| SSC6 | assembly_numt_pipeline | numt_region | 8666730 | 8666806 | . | . | . | . |
| SSC6 | assembly_numt_pipeline | numt_region | 15269114 | 15269180 | . | . | . | . |
| SSC6 | assembly_numt_pipeline | numt_region | 16530986 | 16531051 | . | . | . | . |
| SSC6 | assembly_numt_pipeline | numt_region | 18358888 | 18370127 | . | . | . | . |
| SSC6 | assembly_numt_pipeline | numt_region | 29251200 | 29262491 | . | . | . | . |
| SSC6 | assembly_numt_pipeline | numt_region | 38899892 | 38903943 | . | . | . | . |
| SSC6 | assembly_numt_pipeline | numt_region | 38960245 | 38960495 | . | . | . | . |
| SSC6 | assembly_numt_pipeline | numt_region | 41765754 | 41766029 | . | . | . | . |
| SSC6 | assembly_numt_pipeline | numt_region | 43684389 | 43685114 | . | . | . | . |
| SSC6 | assembly_numt_pipeline | numt_region | 46650106 | 46653314 | . | . | . | . |
| SSC6 | assembly_numt_pipeline | numt_region | 89831911 | 89832105 | . | . | . | . |
| SSC6 | assembly_numt_pipeline | numt_region | 90887348 | 90887443 | . | . | . | . |
| SSC6 | assembly_numt_pipeline | numt_region_insertion_breakpoints | 121262246 | 121262247 | . | . | . | . |
| SSC6 | assembly_numt_pipeline | numt_region | 126256801 | 126256925 | . | . | . | . |
| SSC6 | assembly_numt_pipeline | numt_region | 127736436 | 127744670 | . | . | . | . |
| SSC6 | assembly_numt_pipeline | numt_region | 128729240 | 128734121 | . | . | . | . |
| SSC6 | assembly_numt_pipeline | numt_region | 146806242 | 146806317 | . | . | . | . |
| SSC6 | assembly_numt_pipeline | numt_region | 149060358 | 149060533 | . | . | . | . |
| SSC6 | assembly_numt_pipeline | numt_region_insertion_breakpoints | 152264857 | 152264857 | . | . | . | . |
| SSC6 | assembly_numt_pipeline | numt_region | 156788217 | 156788402 | . | . | . | . |
| SSC6 | assembly_numt_pipeline | numt_region | 159141139 | 159141322 | . | . | . | . |
| SSC6 | assembly_numt_pipeline | numt_region | 170424971 | 170425036 | . | . | . | . |
| SSC6 | assembly_numt_pipeline | numt_region | 170787236 | 170811984 | . | . | . | . |
| SSC7 | assembly_numt_pipeline | numt_region | 1170807 | 1171030 | . | . | . | . |
| SSC7 | assembly_numt_pipeline | numt_region | 10867840 | 10868018 | . | . | . | . |
| SSC7 | assembly_numt_pipeline | numt_region | 26024833 | 26024899 | . | . | . | . |
| SSC7 | assembly_numt_pipeline | numt_region | 30704258 | 30704600 | . | . | . | . |
| SSC7 | assembly_numt_pipeline | numt_region | 37124165 | 37124369 | . | . | . | . |
| SSC7 | assembly_numt_pipeline | numt_region | 47762848 | 47762935 | . | . | . | . |
| SSC7 | assembly_numt_pipeline | numt_region | 50704264 | 50704332 | . | . | . | . |
| SSC7 | assembly_numt_pipeline | numt_region | 54791091 | 54791268 | . | . | . | . |
| SSC7 | assembly_numt_pipeline | numt_region | 55816576 | 55817134 | . | . | . | . |
| SSC7 | assembly_numt_pipeline | numt_region | 55908574 | 55911476 | . | . | . | . |
| SSC7 | assembly_numt_pipeline | numt_region | 56047723 | 56054267 | . | . | . | . |
| SSC7 | assembly_numt_pipeline | numt_region | 77004740 | 77004830 | . | . | . | . |
| SSC7 | assembly_numt_pipeline | numt_region | 77293448 | 77293538 | . | . | . | . |
| SSC7 | assembly_numt_pipeline | numt_region | 82469844 | 82470040 | . | . | . | . |
| SSC7 | assembly_numt_pipeline | numt_region | 98470344 | 98470588 | . | . | . | . |
| SSC7 | assembly_numt_pipeline | numt_region | 111194748 | 111194804 | . | . | . | . |
| SSC7 | assembly_numt_pipeline | numt_region | 111692008 | 111692088 | . | . | . | . |
| SSC8 | assembly_numt_pipeline | numt_region | 12487559 | 12487837 | . | . | . | . |
| SSC8 | assembly_numt_pipeline | numt_region | 16915154 | 16915400 | . | . | . | . |
| SSC8 | assembly_numt_pipeline | numt_region | 22940699 | 22943055 | . | . | . | . |
| SSC8 | assembly_numt_pipeline | numt_region | 32418849 | 32420386 | . | . | . | . |
| SSC8 | assembly_numt_pipeline | numt_region | 51917704 | 51917874 | . | . | . | . |
| SSC8 | assembly_numt_pipeline | numt_region | 54507743 | 54508045 | . | . | . | . |
| SSC8 | assembly_numt_pipeline | numt_region | 72489470 | 72489684 | . | . | . | . |
| SSC8 | assembly_numt_pipeline | numt_region | 88926067 | 88926236 | . | . | . | . |
| SSC8 | assembly_numt_pipeline | numt_region | 99895120 | 99898621 | . | . | . | . |
| SSC8 | assembly_numt_pipeline | numt_region | 101144744 | 101144827 | . | . | . | . |
| SSC8 | assembly_numt_pipeline | numt_region | 105336846 | 105337081 | . | . | . | . |
| SSC8 | assembly_numt_pipeline | numt_region | 118586672 | 118588072 | . | . | . | . |
| SSC8 | assembly_numt_pipeline | numt_region | 127598796 | 127598858 | . | . | . | . |
| SSC9 | assembly_numt_pipeline | numt_region_insertion_breakpoints | 943575 | 943577 | . | . | . | . |
| SSC9 | assembly_numt_pipeline | numt_region | 3235657 | 3235770 | . | . | . | . |
| SSC9 | assembly_numt_pipeline | numt_region | 4700024 | 4702189 | . | . | . | . |
| SSC9 | assembly_numt_pipeline | numt_region | 11394627 | 11395146 | . | . | . | . |
| SSC9 | assembly_numt_pipeline | numt_region | 11540252 | 11540395 | . | . | . | . |
| SSC9 | assembly_numt_pipeline | numt_region | 11712245 | 11712530 | . | . | . | . |
| SSC9 | assembly_numt_pipeline | numt_region | 27326815 | 27326890 | . | . | . | . |
| SSC9 | assembly_numt_pipeline | numt_region | 31715430 | 31715629 | . | . | . | . |
| SSC9 | assembly_numt_pipeline | numt_region | 33290069 | 33291708 | . | . | . | . |
| SSC9 | assembly_numt_pipeline | numt_region | 54035417 | 54035673 | . | . | . | . |
| SSC9 | assembly_numt_pipeline | numt_region | 57271946 | 57272142 | . | . | . | . |
| SSC9 | assembly_numt_pipeline | numt_region | 58506854 | 58506959 | . | . | . | . |
| SSC9 | assembly_numt_pipeline | numt_region | 63747708 | 63752957 | . | . | . | . |
| SSC9 | assembly_numt_pipeline | numt_region | 68174351 | 68174413 | . | . | . | . |
| SSC9 | assembly_numt_pipeline | numt_region | 76359827 | 76360913 | . | . | . | . |
| SSC9 | assembly_numt_pipeline | numt_region_insertion_breakpoints | 88374378 | 88374387 | . | . | . | . |
| SSC9 | assembly_numt_pipeline | numt_region | 91652846 | 91659901 | . | . | . | . |
| SSC9 | assembly_numt_pipeline | numt_region | 118664850 | 118665498 | . | . | . | . |
| SSC9 | assembly_numt_pipeline | numt_region | 123939780 | 123939945 | . | . | . | . |
| SSC9 | assembly_numt_pipeline | numt_region | 126897285 | 126897361 | . | . | . | . |
| SSC9 | assembly_numt_pipeline | numt_region | 132647625 | 132647686 | . | . | . | . |
| SSC9 | assembly_numt_pipeline | numt_region | 139481007 | 139481336 | . | . | . | . |
| SSC10 | assembly_numt_pipeline | numt_region_insertion_breakpoints | 4338880 | 4338881 | . | . | . | . |
| SSC10 | assembly_numt_pipeline | numt_region | 15462597 | 15462763 | . | . | . | . |
| SSC10 | assembly_numt_pipeline | numt_region | 31676524 | 31676600 | . | . | . | . |
| SSC10 | assembly_numt_pipeline | numt_region | 34706921 | 34714307 | . | . | . | . |
| SSC10 | assembly_numt_pipeline | numt_region_insertion_breakpoints | 35223286 | 35223289 | . | . | . | . |
| SSC10 | assembly_numt_pipeline | numt_region | 43875234 | 43875375 | . | . | . | . |
| SSC10 | assembly_numt_pipeline | numt_region | 48894499 | 48894565 | . | . | . | . |
| SSC10 | assembly_numt_pipeline | numt_region | 54531568 | 54531633 | . | . | . | . |
| SSC10 | assembly_numt_pipeline | numt_region | 57342200 | 57342319 | . | . | . | . |
| SSC10 | assembly_numt_pipeline | numt_region | 59664744 | 59664776 | . | . | . | . |
| SSC10 | assembly_numt_pipeline | numt_region | 69342315 | 69358152 | . | . | . | . |
| SSC11 | assembly_numt_pipeline | numt_region | 5470212 | 5470384 | . | . | . | . |
| SSC11 | assembly_numt_pipeline | numt_region | 16621157 | 16621348 | . | . | . | . |
| SSC11 | assembly_numt_pipeline | numt_region | 29441761 | 29442185 | . | . | . | . |
| SSC11 | assembly_numt_pipeline | numt_region | 30076885 | 30077086 | . | . | . | . |
| SSC11 | assembly_numt_pipeline | numt_region | 33665367 | 33665835 | . | . | . | . |
| SSC11 | assembly_numt_pipeline | numt_region | 34048705 | 34048797 | . | . | . | . |
| SSC11 | assembly_numt_pipeline | numt_region | 36778843 | 36779083 | . | . | . | . |
| SSC11 | assembly_numt_pipeline | numt_region | 43225149 | 43225297 | . | . | . | . |
| SSC11 | assembly_numt_pipeline | numt_region | 43655958 | 43656103 | . | . | . | . |
| SSC12 | assembly_numt_pipeline | numt_region | 27510 | 45653 | . | . | . | . |
| SSC12 | assembly_numt_pipeline | numt_region | 29651304 | 29651911 | . | . | . | . |
| SSC12 | assembly_numt_pipeline | numt_region | 30682149 | 30682329 | . | . | . | . |
| SSC12 | assembly_numt_pipeline | numt_region_insertion_breakpoints | 46266888 | 46266888 | . | . | . | . |
| SSC12 | assembly_numt_pipeline | numt_region | 51726250 | 51726392 | . | . | . | . |
| SSC12 | assembly_numt_pipeline | numt_region | 56002455 | 56002573 | . | . | . | . |
| SSC12 | assembly_numt_pipeline | numt_region | 61563908 | 61575296 | . | . | . | . |
| SSC13 | assembly_numt_pipeline | numt_region | 2572580 | 2572644 | . | . | . | . |
| SSC13 | assembly_numt_pipeline | numt_region | 3731614 | 3731778 | . | . | . | . |
| SSC13 | assembly_numt_pipeline | numt_region | 6599959 | 6601185 | . | . | . | . |
| SSC13 | assembly_numt_pipeline | numt_region | 18433234 | 18456126 | . | . | . | . |
| SSC13 | assembly_numt_pipeline | numt_region | 30112905 | 30112945 | . | . | . | . |
| SSC13 | assembly_numt_pipeline | numt_region | 34378339 | 34378479 | . | . | . | . |
| SSC13 | assembly_numt_pipeline | numt_region | 35118944 | 35119060 | . | . | . | . |
| SSC13 | assembly_numt_pipeline | numt_region | 36921850 | 36922087 | . | . | . | . |
| SSC13 | assembly_numt_pipeline | numt_region | 49157355 | 49157418 | . | . | . | . |
| SSC13 | assembly_numt_pipeline | numt_region | 53345118 | 53345230 | . | . | . | . |
| SSC13 | assembly_numt_pipeline | numt_region | 60860508 | 60860580 | . | . | . | . |
| SSC13 | assembly_numt_pipeline | numt_region | 63255892 | 63264761 | . | . | . | . |
| SSC13 | assembly_numt_pipeline | numt_region | 64066404 | 64066658 | . | . | . | . |
| SSC13 | assembly_numt_pipeline | numt_region | 73916170 | 73916326 | . | . | . | . |
| SSC13 | assembly_numt_pipeline | numt_region | 82358298 | 82371131 | . | . | . | . |
| SSC13 | assembly_numt_pipeline | numt_region | 88037046 | 88037165 | . | . | . | . |
| SSC13 | assembly_numt_pipeline | numt_region | 88084171 | 88084364 | . | . | . | . |
| SSC13 | assembly_numt_pipeline | numt_region | 92795157 | 92795199 | . | . | . | . |
| SSC13 | assembly_numt_pipeline | numt_region | 95359337 | 95362652 | . | . | . | . |
| SSC13 | assembly_numt_pipeline | numt_region | 101348510 | 101348600 | . | . | . | . |
| SSC13 | assembly_numt_pipeline | numt_region | 103593988 | 103594542 | . | . | . | . |
| SSC13 | assembly_numt_pipeline | numt_region | 109304325 | 109304527 | . | . | . | . |
| SSC13 | assembly_numt_pipeline | numt_region | 130188499 | 130188566 | . | . | . | . |
| SSC13 | assembly_numt_pipeline | numt_region | 138990007 | 138990086 | . | . | . | . |
| SSC13 | assembly_numt_pipeline | numt_region | 143254773 | 143254882 | . | . | . | . |
| SSC13 | assembly_numt_pipeline | numt_region | 146572142 | 146572487 | . | . | . | . |
| SSC13 | assembly_numt_pipeline | numt_region | 174631057 | 174631233 | . | . | . | . |
| SSC13 | assembly_numt_pipeline | numt_region_insertion_breakpoints | 181947914 | 181947933 | . | . | . | . |
| SSC13 | assembly_numt_pipeline | numt_region | 186470778 | 186470844 | . | . | . | . |
| SSC13 | assembly_numt_pipeline | numt_region | 188199818 | 188199862 | . | . | . | . |
| SSC13 | assembly_numt_pipeline | numt_region | 188225599 | 188225658 | . | . | . | . |
| SSC14 | assembly_numt_pipeline | numt_region | 19741502 | 19741540 | . | . | . | . |
| SSC14 | assembly_numt_pipeline | numt_region | 21808470 | 21808667 | . | . | . | . |
| SSC14 | assembly_numt_pipeline | numt_region | 22157475 | 22166962 | . | . | . | . |
| SSC14 | assembly_numt_pipeline | numt_region | 22348648 | 22361340 | . | . | . | . |
| SSC14 | assembly_numt_pipeline | numt_region | 34087302 | 34099447 | . | . | . | . |
| SSC14 | assembly_numt_pipeline | numt_region | 40641899 | 40642556 | . | . | . | . |
| SSC14 | assembly_numt_pipeline | numt_region | 51687118 | 51704459 | . | . | . | . |
| SSC14 | assembly_numt_pipeline | numt_region | 54876723 | 54876979 | . | . | . | . |
| SSC14 | assembly_numt_pipeline | numt_region | 55147607 | 55147654 | . | . | . | . |
| SSC14 | assembly_numt_pipeline | numt_region | 57471534 | 57471872 | . | . | . | . |
| SSC14 | assembly_numt_pipeline | numt_region | 60744248 | 60775504 | . | . | . | . |
| SSC14 | assembly_numt_pipeline | numt_region | 70688061 | 70688137 | . | . | . | . |
| SSC14 | assembly_numt_pipeline | numt_region | 77599822 | 77599924 | . | . | . | . |
| SSC14 | assembly_numt_pipeline | numt_region | 84301010 | 84301190 | . | . | . | . |
| SSC14 | assembly_numt_pipeline | numt_region | 88191053 | 88203931 | . | . | . | . |
| SSC14 | assembly_numt_pipeline | numt_region | 96062135 | 96062188 | . | . | . | . |
| SSC14 | assembly_numt_pipeline | numt_region | 98469615 | 98469813 | . | . | . | . |
| SSC14 | assembly_numt_pipeline | numt_region | 103682083 | 103682151 | . | . | . | . |
| SSC14 | assembly_numt_pipeline | numt_region | 103831736 | 103831800 | . | . | . | . |
| SSC14 | assembly_numt_pipeline | numt_region | 105834170 | 105834241 | . | . | . | . |
| SSC14 | assembly_numt_pipeline | numt_region | 106523889 | 106524007 | . | . | . | . |
| SSC14 | assembly_numt_pipeline | numt_region | 121657792 | 121684932 | . | . | . | . |
| SSC14 | assembly_numt_pipeline | numt_region | 122883293 | 122883647 | . | . | . | . |
| SSC14 | assembly_numt_pipeline | numt_region_insertion_breakpoints | 135468126 | 135468132 | . | . | . | . |
| SSC14 | assembly_numt_pipeline | numt_region | 141319836 | 141325780 | . | . | . | . |
| SSC15 | assembly_numt_pipeline | numt_region | 5992514 | 5992805 | . | . | . | . |
| SSC15 | assembly_numt_pipeline | numt_region | 9525849 | 9525918 | . | . | . | . |
| SSC15 | assembly_numt_pipeline | numt_region | 15238203 | 15238257 | . | . | . | . |
| SSC15 | assembly_numt_pipeline | numt_region | 21482984 | 21483035 | . | . | . | . |
| SSC15 | assembly_numt_pipeline | numt_region | 24939176 | 24951607 | . | . | . | . |
| SSC15 | assembly_numt_pipeline | numt_region | 31900128 | 31900212 | . | . | . | . |
| SSC15 | assembly_numt_pipeline | numt_region | 36911902 | 36912141 | . | . | . | . |
| SSC15 | assembly_numt_pipeline | numt_region | 47564717 | 47564827 | . | . | . | . |
| SSC15 | assembly_numt_pipeline | numt_region | 56883834 | 56884041 | . | . | . | . |
| SSC15 | assembly_numt_pipeline | numt_region | 58752248 | 58752490 | . | . | . | . |
| SSC15 | assembly_numt_pipeline | numt_region | 59930384 | 59930726 | . | . | . | . |
| SSC15 | assembly_numt_pipeline | numt_region | 73759091 | 73759773 | . | . | . | . |
| SSC15 | assembly_numt_pipeline | numt_region | 76522505 | 76522643 | . | . | . | . |
| SSC15 | assembly_numt_pipeline | numt_region | 78478459 | 78478609 | . | . | . | . |
| SSC15 | assembly_numt_pipeline | numt_region | 80125075 | 80125605 | . | . | . | . |
| SSC15 | assembly_numt_pipeline | numt_region | 90309357 | 90310192 | . | . | . | . |
| SSC15 | assembly_numt_pipeline | numt_region | 99876288 | 99876464 | . | . | . | . |
| SSC15 | assembly_numt_pipeline | numt_region | 113871270 | 113871405 | . | . | . | . |
| SSC15 | assembly_numt_pipeline | numt_region | 115843721 | 115843768 | . | . | . | . |
| SSC15 | assembly_numt_pipeline | numt_region | 117468558 | 117468640 | . | . | . | . |
| SSC15 | assembly_numt_pipeline | numt_region | 121728589 | 121728817 | . | . | . | . |
| SSC15 | assembly_numt_pipeline | numt_region | 126761413 | 126762898 | . | . | . | . |
| SSC15 | assembly_numt_pipeline | numt_region | 132810794 | 132810866 | . | . | . | . |
| SSC15 | assembly_numt_pipeline | numt_region | 133107704 | 133108539 | . | . | . | . |
| SSC15 | assembly_numt_pipeline | numt_region | 138241100 | 138241148 | . | . | . | . |
| SSC16 | assembly_numt_pipeline | numt_region | 7226028 | 7226299 | . | . | . | . |
| SSC16 | assembly_numt_pipeline | numt_region | 11335808 | 11336006 | . | . | . | . |
| SSC16 | assembly_numt_pipeline | numt_region | 11521622 | 11521881 | . | . | . | . |
| SSC16 | assembly_numt_pipeline | numt_region | 37934962 | 37935189 | . | . | . | . |
| SSC16 | assembly_numt_pipeline | numt_region | 38314203 | 38314434 | . | . | . | . |
| SSC16 | assembly_numt_pipeline | numt_region | 54124476 | 54124671 | . | . | . | . |
| SSC16 | assembly_numt_pipeline | numt_region_insertion_breakpoints | 63009645 | 63009651 | . | . | . | . |
| SSC16 | assembly_numt_pipeline | numt_region | 63200500 | 63200630 | . | . | . | . |
| SSC16 | assembly_numt_pipeline | numt_region | 73831858 | 73831936 | . | . | . | . |
| SSC16 | assembly_numt_pipeline | numt_region | 77228859 | 77228918 | . | . | . | . |
| SSC17 | assembly_numt_pipeline | numt_region | 373375 | 373738 | . | . | . | . |
| SSC17 | assembly_numt_pipeline | numt_region | 3230345 | 3230404 | . | . | . | . |
| SSC17 | assembly_numt_pipeline | numt_region | 4839732 | 4839847 | . | . | . | . |
| SSC17 | assembly_numt_pipeline | numt_region | 11261181 | 11261288 | . | . | . | . |
| SSC17 | assembly_numt_pipeline | numt_region | 14227602 | 14233979 | . | . | . | . |
| SSC17 | assembly_numt_pipeline | numt_region | 19235662 | 19235781 | . | . | . | . |
| SSC17 | assembly_numt_pipeline | numt_region | 30074486 | 30074542 | . | . | . | . |
| SSC17 | assembly_numt_pipeline | numt_region | 31281724 | 31297092 | . | . | . | . |
| SSC17 | assembly_numt_pipeline | numt_region | 36650620 | 36650656 | . | . | . | . |
| SSC17 | assembly_numt_pipeline | numt_region | 49421797 | 49421917 | . | . | . | . |
| SSC17 | assembly_numt_pipeline | numt_region | 51947486 | 51947529 | . | . | . | . |
| SSC17 | assembly_numt_pipeline | numt_region | 63097682 | 63116188 | . | . | . | . |
| SSC18 | assembly_numt_pipeline | numt_region | 4305593 | 4305651 | . | . | . | . |
| SSC18 | assembly_numt_pipeline | numt_region | 14833095 | 14833208 | . | . | . | . |
| SSC18 | assembly_numt_pipeline | numt_region | 19402494 | 19402576 | . | . | . | . |
| SSC18 | assembly_numt_pipeline | numt_region | 27644348 | 27644657 | . | . | . | . |
| SSC18 | assembly_numt_pipeline | numt_region | 29854902 | 29855039 | . | . | . | . |
| SSC18 | assembly_numt_pipeline | numt_region | 33667469 | 33668587 | . | . | . | . |
| SSC18 | assembly_numt_pipeline | numt_region | 37434521 | 37434593 | . | . | . | . |
| SSC18 | assembly_numt_pipeline | numt_region | 55601137 | 55601277 | . | . | . | . |
| SSCX | assembly_numt_pipeline | numt_region | 5048053 | 5048127 | . | . | . | . |
| SSCX | assembly_numt_pipeline | numt_region | 6199316 | 6199381 | . | . | . | . |
| SSCX | assembly_numt_pipeline | numt_region | 10110581 | 10110669 | . | . | . | . |
| SSCX | assembly_numt_pipeline | numt_region | 13324152 | 13325081 | . | . | . | . |
| SSCX | assembly_numt_pipeline | numt_region | 15135618 | 15135851 | . | . | . | . |
| SSCX | assembly_numt_pipeline | numt_region | 22061759 | 22074446 | . | . | . | . |
| SSCX | assembly_numt_pipeline | numt_region | 28472452 | 28473578 | . | . | . | . |
| SSCX | assembly_numt_pipeline | numt_region | 45543880 | 45543984 | . | . | . | . |
| SSCX | assembly_numt_pipeline | numt_region | 60608172 | 60608214 | . | . | . | . |
| SSCX | assembly_numt_pipeline | numt_region | 71824292 | 71831135 | . | . | . | . |
| SSCX | assembly_numt_pipeline | numt_region | 72211696 | 72211860 | . | . | . | . |
| SSCX | assembly_numt_pipeline | numt_region | 82426850 | 82427076 | . | . | . | . |
| SSCX | assembly_numt_pipeline | numt_region | 91603293 | 91603329 | . | . | . | . |
| SSCX | assembly_numt_pipeline | numt_region | 111402642 | 111402709 | . | . | . | . |
| SSCX | assembly_numt_pipeline | numt_region | 116377250 | 116377321 | . | . | . | . |
| SSCY | assembly_numt_pipeline | numt_region | 2930682 | 2930771 | . | . | . | . |
| SSCY | assembly_numt_pipeline | numt_region | 4622617 | 4622682 | . | . | . | . |
| NW_018084826.1 | assembly_numt_pipeline | numt_region | 124606 | 126069 | . | . | . | . |
| NW_018084861.1 | assembly_numt_pipeline | numt_region | 12827 | 45423 | . | . | . | . |
| NW_018084868.1 | assembly_numt_pipeline | numt_region | 11554 | 22719 | . | . | . | . |
| NW_018084889.1 | assembly_numt_pipeline | numt_region | 10724 | 28897 | . | . | . | . |
| NW_018084908.1 | assembly_numt_pipeline | numt_region | 66695 | 74424 | . | . | . | . |
| NW_018084924.1 | assembly_numt_pipeline | numt_region | 13234 | 13356 | . | . | . | . |
| NW_018085004.1 | assembly_numt_pipeline | numt_region | 1185427 | 1185484 | . | . | . | . |
| NW_018085094.1 | assembly_numt_pipeline | numt_region | 93485 | 93622 | . | . | . | . |
| NW_018085094.1 | assembly_numt_pipeline | numt_region | 447433 | 447739 | . | . | . | . |
| NW_018085141.1 | assembly_numt_pipeline | numt_region | 35028 | 53351 | . | . | . | . |
| NW_018085176.1 | assembly_numt_pipeline | numt_region | 24767 | 42904 | . | . | . | . |
| NW_018085200.1 | assembly_numt_pipeline | numt_region | 1021907 | 1021966 | . | . | . | . |
| NW_018085231.1 | assembly_numt_pipeline | numt_region | 42708 | 42823 | . | . | . | . |
| NW_018085254.1 | assembly_numt_pipeline | numt_region | 40733 | 52114 | . | . | . | . |
| NW_018085299.1 | assembly_numt_pipeline | numt_region | 32397 | 32487 | . | . | . | . |
| SSC1 | wgs_numt_pipeline | numt_region_insertion_breakpoints | 39572388 | 39572450 | . | . | . | . |
| SSC1 | wgs_numt_pipeline | numt_region_insertion_breakpoints | 57972715 | 57972811 | . | . | . | . |
| SSC1 | wgs_numt_pipeline | numt_region_insertion_breakpoints | 68876321 | 68876410 | . | . | . | . |
| SSC1 | wgs_numt_pipeline | numt_region_insertion_breakpoints | 92704562 | 92704661 | . | . | . | . |
| SSC1 | wgs_numt_pipeline | numt_region_insertion_breakpoints | 156314048 | 156314175 | . | . | . | . |
| SSC1 | wgs_numt_pipeline | numt_region_insertion_breakpoints | 204816326 | 204816407 | . | . | . | . |
| SSC1 | wgs_numt_pipeline | numt_region_insertion_breakpoints | 226134840 | 226134918 | . | . | . | . |
| SSC1 | wgs_numt_pipeline | numt_region_insertion_breakpoints | 228145697 | 228145804 | . | . | . | . |
| SSC2 | wgs_numt_pipeline | numt_region_insertion_breakpoints | 52215335 | 52215447 | . | . | . | . |
| SSC2 | wgs_numt_pipeline | numt_region_insertion_breakpoints | 143024387 | 143024446 | . | . | . | . |
| SSC3 | wgs_numt_pipeline | numt_region_insertion_breakpoints | 30018166 | 30018272 | . | . | . | . |
| SSC3 | wgs_numt_pipeline | numt_region_insertion_breakpoints | 41834747 | 41834862 | . | . | . | . |
| SSC3 | wgs_numt_pipeline | numt_region_insertion_breakpoints | 64738370 | 64738477 | . | . | . | . |
| SSC3 | wgs_numt_pipeline | numt_region_insertion_breakpoints | 89955750 | 89955825 | . | . | . | . |
| SSC4 | wgs_numt_pipeline | numt_region_insertion_breakpoints | 11167530 | 11167626 | . | . | . | . |
| SSC4 | wgs_numt_pipeline | numt_region_insertion_breakpoints | 28097681 | 28097800 | . | . | . | . |
| SSC4 | wgs_numt_pipeline | numt_region_insertion_breakpoints | 129631831 | 129631937 | . | . | . | . |
| SSC5 | wgs_numt_pipeline | numt_region_insertion_breakpoints | 15720083 | 15720150 | . | . | . | . |
| SSC5 | wgs_numt_pipeline | numt_region_insertion_breakpoints | 25919957 | 25920060 | . | . | . | . |
| SSC5 | wgs_numt_pipeline | numt_region_insertion_breakpoints | 75804895 | 75805048 | . | . | . | . |
| SSC6 | wgs_numt_pipeline | numt_region_insertion_breakpoints | 6587550 | 6587620 | . | . | . | . |
| SSC6 | wgs_numt_pipeline | numt_region_insertion_breakpoints | 14006516 | 14006572 | . | . | . | . |
| SSC6 | wgs_numt_pipeline | numt_region_insertion_breakpoints | 20570823 | 20570946 | . | . | . | . |
| SSC6 | wgs_numt_pipeline | numt_region_insertion_breakpoints | 33718882 | 33718950 | . | . | . | . |
| SSC6 | wgs_numt_pipeline | numt_region_insertion_breakpoints | 51865566 | 51865635 | . | . | . | . |
| SSC6 | wgs_numt_pipeline | numt_region_insertion_breakpoints | 122877223 | 122877301 | . | . | . | . |
| SSC6 | wgs_numt_pipeline | numt_region_insertion_breakpoints | 154189059 | 154189167 | . | . | . | . |
| SSC6 | wgs_numt_pipeline | numt_region_insertion_breakpoints | 154418128 | 154418215 | . | . | . | . |
| SSC6 | wgs_numt_pipeline | numt_region_insertion_breakpoints | 161687433 | 161687490 | . | . | . | . |
| SSC7 | wgs_numt_pipeline | numt_region_insertion_breakpoints | 101752313 | 101752364 | . | . | . | . |
| SSC8 | wgs_numt_pipeline | numt_region_insertion_breakpoints | 16799830 | 16799892 | . | . | . | . |
| SSC8 | wgs_numt_pipeline | numt_region_insertion_breakpoints | 61867571 | 61867645 | . | . | . | . |
| SSC8 | wgs_numt_pipeline | numt_region_insertion_breakpoints | 80294335 | 80294408 | . | . | . | . |
| SSC8 | wgs_numt_pipeline | numt_region_insertion_breakpoints | 99737278 | 99737360 | . | . | . | . |
| SSC8 | wgs_numt_pipeline | numt_region_insertion_breakpoints | 109762102 | 109762184 | . | . | . | . |
| SSC9 | wgs_numt_pipeline | numt_region_insertion_breakpoints | 3919319 | 3919456 | . | . | . | . |
| SSC9 | wgs_numt_pipeline | numt_region_insertion_breakpoints | 17455187 | 17455270 | . | . | . | . |
| SSC9 | wgs_numt_pipeline | numt_region_insertion_breakpoints | 71580848 | 71580961 | . | . | . | . |
| SSC9 | wgs_numt_pipeline | numt_region_insertion_breakpoints | 83479115 | 83479221 | . | . | . | . |
| SSC9 | wgs_numt_pipeline | numt_region_insertion_breakpoints | 125919000 | 125919094 | . | . | . | . |
| SSC10 | wgs_numt_pipeline | numt_region_insertion_breakpoints | 777231 | 777324 | . | . | . | . |
| SSC10 | wgs_numt_pipeline | numt_region_insertion_breakpoints | 9719101 | 9719185 | . | . | . | . |
| SSC10 | wgs_numt_pipeline | numt_region_insertion_breakpoints | 20857112 | 20857182 | . | . | . | . |
| SSC10 | wgs_numt_pipeline | numt_region_insertion_breakpoints | 31027267 | 31027347 | . | . | . | . |
| SSC11 | wgs_numt_pipeline | numt_region_insertion_breakpoints | 40148001 | 40148104 | . | . | . | . |
| SSC11 | wgs_numt_pipeline | numt_region_insertion_breakpoints | 58201230 | 58201325 | . | . | . | . |
| SSC11 | wgs_numt_pipeline | numt_region_insertion_breakpoints | 60349989 | 60350043 | . | . | . | . |
| SSC11 | wgs_numt_pipeline | numt_region_insertion_breakpoints | 61166916 | 61167008 | . | . | . | . |
| SSC11 | wgs_numt_pipeline | numt_region_insertion_breakpoints | 66249992 | 66250125 | . | . | . | . |
| SSC11 | wgs_numt_pipeline | numt_region_insertion_breakpoints | 73847043 | 73847123 | . | . | . | . |
| SSC12 | wgs_numt_pipeline | numt_region_insertion_breakpoints | 28728030 | 28728102 | . | . | . | . |
| SSC13 | wgs_numt_pipeline | numt_region_insertion_breakpoints | 2750675 | 2750736 | . | . | . | . |
| SSC13 | wgs_numt_pipeline | numt_region_insertion_breakpoints | 9074691 | 9074770 | . | . | . | . |
| SSC13 | wgs_numt_pipeline | numt_region_insertion_breakpoints | 32296347 | 32296462 | . | . | . | . |
| SSC13 | wgs_numt_pipeline | numt_region_insertion_breakpoints | 52153208 | 52153285 | . | . | . | . |
| SSC13 | wgs_numt_pipeline | numt_region_insertion_breakpoints | 110305664 | 110305731 | . | . | . | . |
| SSC13 | wgs_numt_pipeline | numt_region_insertion_breakpoints | 130756555 | 130756630 | . | . | . | . |
| SSC13 | wgs_numt_pipeline | numt_region_insertion_breakpoints | 135129711 | 135129765 | . | . | . | . |
| SSC13 | wgs_numt_pipeline | numt_region_insertion_breakpoints | 167401690 | 167401916 | . | . | . | . |
| SSC14 | wgs_numt_pipeline | numt_region_insertion_breakpoints | 99267164 | 99267246 | . | . | . | . |
| SSC14 | wgs_numt_pipeline | numt_region_insertion_breakpoints | 135848690 | 135848752 | . | . | . | . |
| SSC15 | wgs_numt_pipeline | numt_region_insertion_breakpoints | 135212315 | 135212418 | . | . | . | . |
| SSC16 | wgs_numt_pipeline | numt_region_insertion_breakpoints | 2937735 | 2937810 | . | . | . | . |
| SSC16 | wgs_numt_pipeline | numt_region_insertion_breakpoints | 7273240 | 7273329 | . | . | . | . |
| SSC16 | wgs_numt_pipeline | numt_region_insertion_breakpoints | 20253369 | 20253439 | . | . | . | . |
| SSC16 | wgs_numt_pipeline | numt_region_insertion_breakpoints | 39903144 | 39903231 | . | . | . | . |
| SSC16 | wgs_numt_pipeline | numt_region_insertion_breakpoints | 52280446 | 52280524 | . | . | . | . |
| SSC16 | wgs_numt_pipeline | numt_region_insertion_breakpoints | 59018885 | 59018962 | . | . | . | . |
| SSC17 | wgs_numt_pipeline | numt_region_insertion_breakpoints | 379534 | 379674 | . | . | . | . |
| SSC17 | wgs_numt_pipeline | numt_region_insertion_breakpoints | 16684277 | 16684336 | . | . | . | . |
